# Early Glycemic Variability from Routine Glucose Measurements, Diabetes Status, and In-Hospital Mortality in Sepsis: A 14-Year Retrospective Cohort Study

**DOI:** 10.3390/jcm15218649

**Published:** 2026-11-06

**Authors:** Florentina Mușat, Octavian Andronic, Alexandra Bolocan, Alina Mihaela Dobre, Daniel Ion, Dan Nicolae Păduraru

**Affiliations:** 1 Faculty of Medicine, Carol Davila University of Medicine and Pharmacy, 050474 Bucharest, Romania; florentina.musat@drd.umfcd.ro (F.M.); alexandra.bolocan@umfcd.ro (A.B.); alina.dobre@drd.umfcd.ro (A.M.D.); daniel.ion@umfcd.ro (D.I.); dan.paduraru@umfcd.ro (D.N.P.); 2 Innovation and eHealth Center, Carol Davila University of Medicine and Pharmacy, 010451 Bucharest, Romania; 3 C.F.2 Clinical Hospital, 011464 Bucharest, Romania; 4 General Surgery Department, University Emergency Hospital of Bucharest, 050098 Bucharest, Romania

**Keywords:** sepsis, diabetes mellitus, glycemic variability, coefficient of variation, intermittent glucose monitoring, hypoglycemia, hospital mortality, effect modification, prognosis

## Abstract

**Background/Objectives**: The prognostic meaning of early glycemic variability in sepsis may depend on whether the patient has diabetes. We examined the association of pre-existing diabetes with in-hospital mortality and tested whether diabetes modifies the prognostic association of early glycemic variability estimated from routine glucose measurements. **Methods**: This study uses a single-center retrospective cohort of 10,832 adults with sepsis identified from discharge documentation at a Romanian tertiary hospital (2011–2024). Early glycemic variability was the coefficient of variation (CV) of glucose within 24 h of the first measurement, computable in 6450 patients (59.5%). Logistic models using diabetes-by-glycemic-parameter interactions were complemented by stratified, landmark and weighted analyses and by standardized absolute risks. **Results**: In-hospital mortality was 53.6%. Diabetes was associated with lower adjusted odds of death (odds ratio [OR] of 0.822; 95% confidence interval [CI] of 0.751–0.899), corresponding to a standardized mortality of 50.5% vs. 54.8% (risk difference, −4.3 percentage points); the difference was smaller among patients alive 24 h after the first measurement (−2.8 percentage points). Per 0.10 increase in CV-24 h, the OR for death was 1.137 (1.102–1.173) without and 1.034 (0.994–1.076) with diabetes (interaction *p* < 0.001); diabetes also modified the associations of mean glucose and hyperglycemia. The CV interaction persisted after weighting and among 24 h survivors but was attenuated when deaths within 48–72 h were excluded; CV added little to the discrimination. **Conclusions**: In this observational cohort, the same degree of early glycemic variability carried different prognostic meaning, mainly for short-term mortality, in patients with and without diabetes. Diabetes was associated with modestly lower observed mortality, which should not be read as a protective effect. These are prognostic associations, not independent effects, and they do not define glucose targets.

## 1. Introduction

Sepsis remains one of the leading causes of in-hospital death. In a pooled analysis of population-level studies, 26.7% of patients with hospital-treated sepsis, and 41.9% of those treated in intensive care, died before discharge [1]. Prognosis varies widely with host characteristics and with the source of infection: in a multicenter cohort of 7974 patients with septic shock, crude hospital mortality ranged from 21% to 85% across sources [2]. Risk stratification is particularly difficult outside intensive care units, where structured severity scores are inconsistently documented. Simple markers that are measured early and almost universally are therefore attractive as adjuncts, and blood glucose is one of the few analytes obtained in nearly every septic patient within hours of presentation and repeated as part of routine care.

Beyond its level, the early behavior of glucose carries prognostic information. Admission hyperglycemia, hypoglycemia and short-term glycemic variability have each been associated with higher mortality in sepsis [3,4,5]. Variability captures the amplitude of glucose excursions rather than their level. In this study, early glycemic variability means the dispersion of the glucose values obtained during the first 24–72 h of routine care, expressed as the coefficient of variation; unlike the standard deviation, the coefficient of variation does not increase simply because glucose is higher. Because routine sampling is sparse, this measure is not equivalent to variability from continuous or high-frequency monitoring. Its meaning may also depend on the patient’s chronic metabolic state. In a patient with previously normal glucose regulation, high early variability is an acute departure from physiological set points and plausibly reflects the intensity of the stress response. In a patient with diabetes, similar variability may largely reflect pre-existing metabolic instability and glucose-lowering treatment. If so, the same numerical value would carry different prognostic meaning in the two groups, and diabetes would act both as a possible risk factor and as a modifier of the prognostic value of acute dysglycemia, the term used here for the early glucose disturbances of a group acute illness, i.e., hyperglycemia, hypoglycemia, an abnormal glucose level and glycemic variability.

Diabetes is therefore central to the question. It is a chronic disorder of persistent hyperglycemia with widespread alterations in immune and vascular function, and patients with diabetes have long been recognized as susceptible to infection, which is now attributed to impaired innate and adaptive immunity, endothelial dysfunction and metabolic derangement [6]. Because sepsis is a dysregulated host response to infection, leading to organ dysfunction [7], diabetes might plausibly influence both the incidence and the course of sepsis.

Biological arguments point in both directions. Diabetes impairs neutrophil function and phagocytosis, alters cytokine production, causes endothelial dysfunction and amplifies inflammatory responses, all of which may impair pathogen clearance and promote organ injury [8,9]. Clinically, patients with diabetes and sepsis have shown higher rates of urinary tract and fungal infection, higher inflammatory markers, higher Acute Physiology and Chronic Health Evaluation (APACHE) II scores and higher mortality [10]. Diabetes has not consistently been associated with a higher incidence of acute kidney injury in severe sepsis, but it has been associated with persistent renal dysfunction once injury has occurred [11]. On the other hand, several mechanisms could produce a neutral or inverse association. Chronic exposure to hyperglycemia might blunt the harm of acute glucose disturbance [12,13], although direct evidence in human sepsis is lacking. Patients with known diabetes are usually under closer glucose surveillance and more active glycemic management in hospital, whereas patients without diabetes who develop stress hyperglycemia (the hyperglycemia of acute illness in a patient without known diabetes) may be monitored less intensively despite a higher risk profile. Part of any inverse association may reflect treatment rather than disease: preadmission metformin use has been associated with lower mortality in septic patients with diabetes [14]. Several of these mechanisms act through the glycemic response itself, so that chronic diabetes status and acute glycemic behavior cannot be interpreted independently of each other.

The published evidence is unresolved. Two recent meta-analyses of partly overlapping literature reached opposite conclusions, one finding no excess risk and the other a higher risk [3,15]; in the first, admission hyperglycemia rather than diabetes was associated with mortality [3]. A few studies have examined the two dimensions jointly. In critically ill patients, glycemic variability was associated with mortality in patients without diabetes but not in those with diabetes [16]; in septic intensive care patients, the association between early glycemia and mortality differed according to insulin-treated diabetes [17]; and in a large critical care dataset of sepsis-associated acute kidney injury, the association between variability and mortality was absent in diabetic subgroups [18]. These signals point in the same direction, but all three studies were conducted in intensive care, variability was mostly assessed over the entire stay or with cumulative metrics [5], and diabetes was examined as a selected subgroup [17] or in subgroup analyses of cohorts assembled for other purposes [18] rather than as a formally tested effect modifier. Whether early glycemic variability derived from the sparse sampling of routine care carries the same prognostic meaning in patients with and without diabetes has not, to our knowledge, been formally tested in an unselected sepsis cohort, and data from Eastern Europe are scarce.

We therefore examined diabetes status and early glycemic variability together in a 14-year cohort of consecutive adults hospitalized with sepsis. The primary objective was the association between pre-existing diabetes and in-hospital mortality. The principal hypothesis was that early glycemic variability carries different prognostic information according to diabetes status, tested as the interaction between pre-existing diabetes and the coefficient of variation of glucose in the first 24 h. Secondary objectives were to describe the early glycemic profile by diabetes status; to estimate the independent associations of early glycemic parameters with in-hospital mortality; to test, with interaction and stratified analyses, whether these associations differed by diabetes status; to examine their consistency across 48 h and 72 h windows; and to explore the shape of the association between early glycemic variability and mortality.

## 2. Materials and Methods

### 2.1. Study Design and Setting

We conducted a retrospective observational cohort study of adults hospitalized with sepsis at the University Emergency Hospital of Bucharest, a tertiary university emergency hospital in Romania, between 1 January 2011 and 31 December 2024, using electronic health record (EHR) data. Extracted variables were demographic and clinical characteristics, comorbidities, documented infection foci, in-hospital interventions, discharge status, and all glucose values with their timestamps. There was no written hospital-wide glucose-management protocol during the study period; glucose monitoring and treatment were at the discretion of the treating physicians. Medication, nutrition and glucose-management decisions are not captured in the coded record and were not available. The study is reported according to the Strengthening the Reporting of Observational Studies in Epidemiology (STROBE) statement [19]. Because the onset of sepsis could not be reconstructed, admission of the index hospitalization defined time zero for cohort entry, covariate assessment and outcome ascertainment; the glycemic exposure windows were anchored to the first glucose measurement of the index presentation (Section 2.5).

### 2.2. Participants and Sepsis Ascertainment

**Figure 1:**
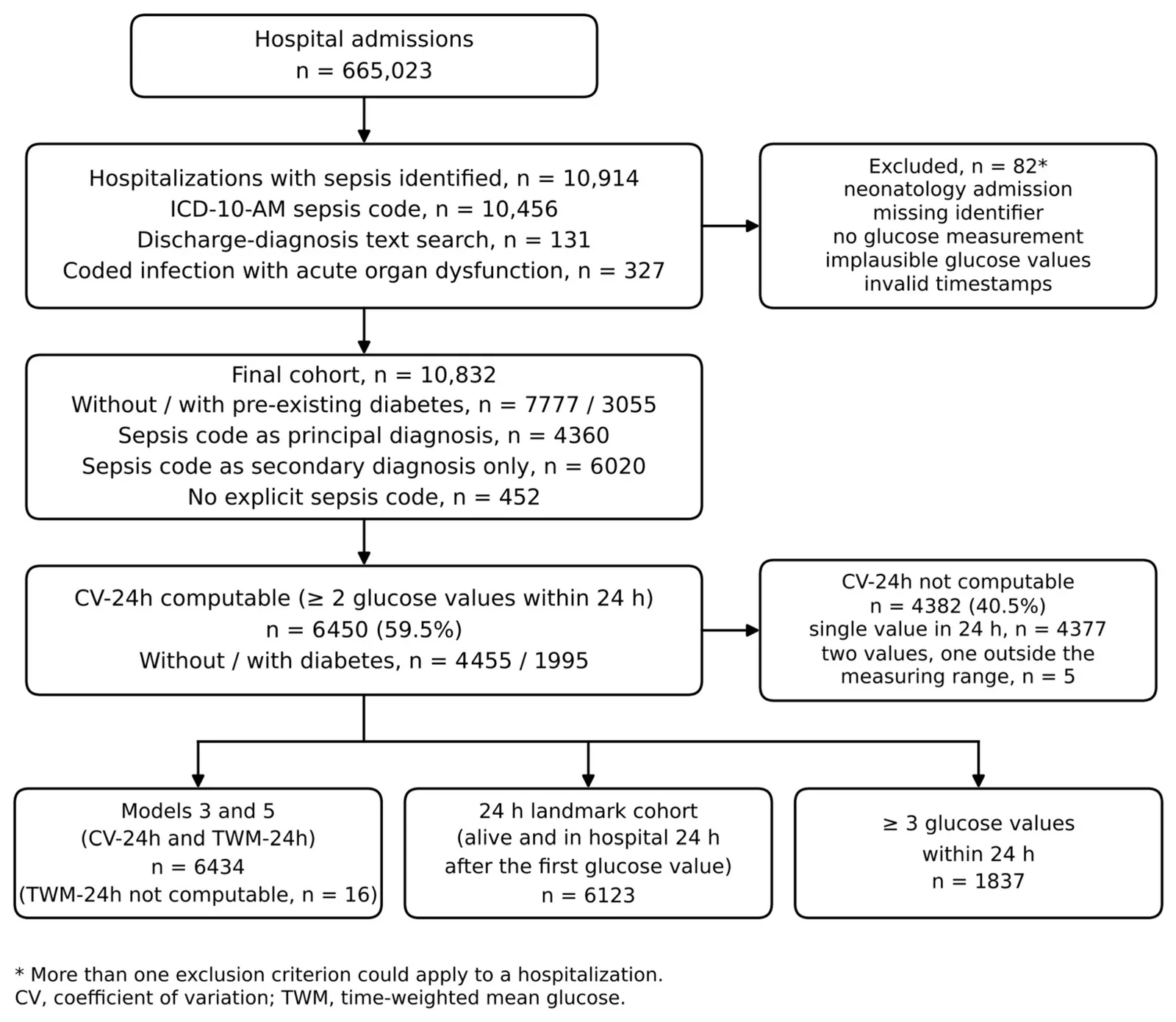
Flowchart of cohort selection, sepsis identification, diabetes status and analytic populations. Counts in the sepsis identification box refer to hospitalizations before exclusions. The asterisk indicates that more than one exclusion criterion could apply to a given patient. All analytic denominators and the number of deaths in each analysis are reconciled in Table S36. CV, coefficient of variation.

All hospital admissions during the study period (n = 665,023) were screened in three steps (Figure 1). Diagnoses are coded at discharge in Romanian hospitals using the International Statistical Classification of Diseases and Related Health Problems, Tenth Revision, Australian Modification (ICD-10-AM). First, admissions were screened for ICD-10-AM codes compatible with sepsis as principal or secondary discharge diagnoses: A02.1, A26.7, A32.7, A40.0, A40.1, A40.2, A40.8, A40.9, A41.0, A41.1, A41.2, A41.3, A41.4, A41.50, A41.51, A41.52, A41.58, A41.8, A41.9, A42.7, B37.7, T81.42 and O88.3 (frequencies in Table S1). The code for septic shock (R57.2) was not used in the hospital record during the study period, so septic shock was identifiable only from the free-text discharge diagnosis. Second, discharge diagnosis text was searched for “sepsis”, “septic” and “septic shock” using a case-insensitive substring search, without stemming, spelling correction, whitespace normalization or alternative-language terms. Third, in hospitalizations without a sepsis code or text label, sepsis was assigned only when the record documented both a coded infection and a coded acute organ dysfunction (respiratory, cardiovascular or shock, renal, hepatic, neurological or coagulation). This third route is an administrative approximation of the Sepsis-3 criteria [20], not a prospective clinical assessment. Because complete Sequential Organ Failure Assessment (SOFA) components were not uniformly available, organ dysfunction was identified from coded diagnoses rather than from physiological measurements.

Eligible participants were aged 18 years or older with sepsis documented during the index hospitalization according to this algorithm. Exclusion criteria were neonatology admissions, missing patient identifier, absence of glucose measurements during hospitalization, implausible glucose values (such as negative results), and inconsistent or non-chronological timestamps that could not be corrected. The algorithm identified 10,914 hospitalizations with sepsis (10,456 by ICD-10-AM codes, 131 by text search and 327 by coded infection with organ dysfunction); after exclusion of 82, the final cohort comprised 10,832 patients (Figure 1). In the final cohort, sepsis was identified by a sepsis code as principal diagnosis (n = 4360); by a sepsis code as secondary diagnosis only (n = 6020); or without an explicit sepsis code via text search but with coded infection with organ dysfunction (n = 452). A coded acute organ dysfunction, the closest available approximation of the organ-dysfunction component of Sepsis-3 (organ systems and codes in Section 2.4, including septic shock documented in the discharge text), was present in 6755 patients (62.4%) and absent in 4077 (37.6%) (Table 1). The ascertainment subgroups of Table S28 use organ dysfunction identified from diagnosis codes alone: 6140 of the 10,380 patients with an explicit sepsis code (59.2%) had such a code. The principal analyses were repeated in these subgroups (Section 2.9.7). No patient had more than one hospitalization with sepsis during the study period; each patient, therefore, contributed a single observation.

Because the study period spanned changes in sepsis definitions and coding practice, case ascertainment relied on the contemporaneous clinician-assigned diagnosis and on the administrative documentation available at the time rather than on retrospective reclassification under a single definition. Calendar year was therefore included in all adjusted analyses, and the principal analyses were repeated within calendar periods (Section 2.9.7).

### 2.3. Outcomes

The outcome was in-hospital mortality, defined as death before hospital discharge. Post-discharge vital status was not available.

### 2.4. Exposures, Covariates, and Effect Modifier

Pre-existing diabetes mellitus was identified from the ICD-10-AM diagnosis codes recorded in the EHR: patients with at least one code in categories E10–E14 among the principal or secondary discharge diagnoses were classified as having pre-existing diabetes. Codes for hyperglycemia without a diagnosis of diabetes (R73.x) were not counted as diabetes. The diabetes codes observed in the cohort are listed in Table S2. Type of diabetes was recorded from the code category (E10, type 1; E11, type 2; E13 and E14, other or unspecified). Insulin treatment could not be identified because the code for long-term insulin use (Z79.4) was not recorded in the cohort, and no medication or disease-duration data were available; glycated hemoglobin was not routinely measured and is not available for the cohort. Diabetes status in this study, therefore, means documented diabetes, not an objectively established chronic glycemic state.

The primary exposure domain was early glycemic status, assessed from glucose measurements within 24 h of the first measurement of the index presentation (Section 2.5). Pre-existing diabetes was treated both as an exposure of interest and as an effect modifier in interaction and stratified analyses.

**Figure 2:**
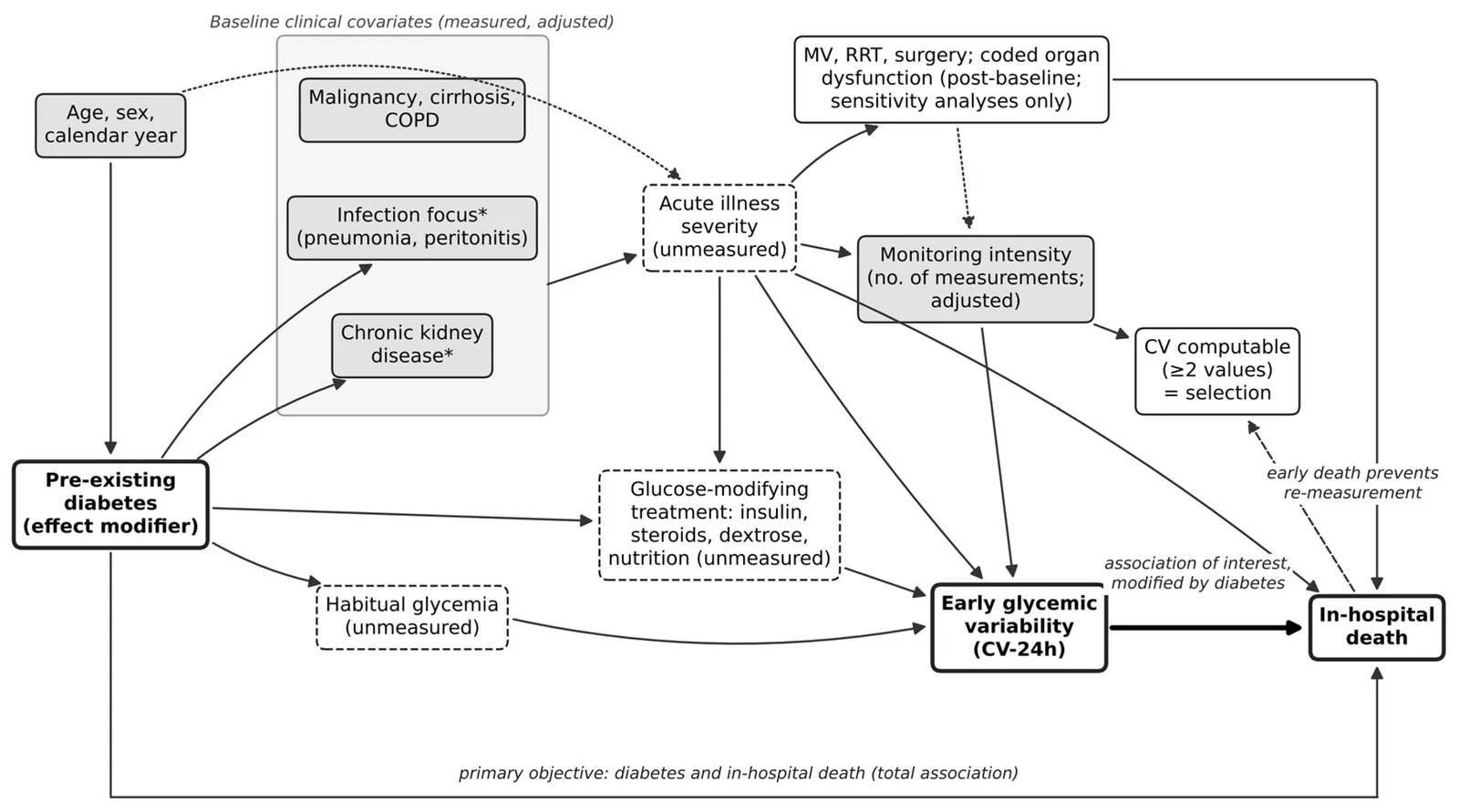
Conceptual causal model (directed acyclic graph) illustrating the analyses. Gray boxes indicate measured variables included in the models; dashed boxes, unmeasured variables; white boxes with solid outline, post-baseline variables and the selection imposed by the requirement of at least two glucose measurements for CV. Bold boxes indicate pre-existing diabetes (exposure for the primary objective and effect modifier for the principal hypothesis), early glycemic variability and in-hospital death. Chronic kidney disease and infection focus (*) may mediate part of the association between diabetes and death; estimates were unchanged when they were omitted (Table S34). Age, sex, calendar year and the baseline clinical covariates also act directly on in-hospital death; these arrows are omitted for clarity. The dashed arrow from death to the selection node indicates that patients who die before a second glucose measurement cannot enter the CV analyses. COPD, chronic obstructive pulmonary disease; CV, coefficient of variation; MV, mechanical ventilation; RRT, renal replacement therapy.

Baseline covariates were age, sex, year of admission, malignancy, chronic kidney disease (CKD), cirrhosis, chronic obstructive pulmonary disease (COPD), and two documented infection foci, i.e., pneumonia and peritonitis. Covariates were selected on the basis of a conceptual causal model (Figure 2). Age, sex and calendar year were treated as confounders; malignancy, cirrhosis and COPD as prognostic factors not plausibly caused by diabetes; and CKD and the infection focus as covariates that may partly mediate the association between diabetes and death. Acute illness severity was the principal unmeasured confounder of the association between glycemic variability and death; the number of glucose measurements was included as a determinant of how variability was measured. Clostridioides difficile infection was recorded as a descriptive variable and was not included in the adjusted models. Mechanical ventilation, renal replacement therapy and surgical intervention were recorded only as binary in-hospital variables without timing and were therefore entered only in sensitivity analyses. The number of organ systems with a coded acute dysfunction was derived from the discharge diagnoses: respiratory (J96.0, J96.9, J80); cardiovascular (shock, R57.x, or septic shock documented in the discharge diagnosis text); renal (N17.x); coagulation (D65, D69.5, D69.6); hepatic (K72.0, K72.9, K76.7); neurological (R40.2, G93.4, F05.x); and metabolic acidosis (E87.2); cardiac arrest (I46.x) was not counted. Because these codes carry no timing and may reflect dysfunction arising after the exposure window, this variable was used only in sensitivity analyses (Section 2.9.7).

### 2.5. Glucose Data Extraction and Time Windows

All blood glucose measurements recorded during hospitalization were extracted with their timestamps and ordered chronologically for each patient. All values were laboratory measurements; point-of-care capillary values are not stored in the EHR and were not available. Early glycemic exposure was assessed in 24, 48 and 72 h windows starting from the first glucose measurement of the index presentation. In 9380 patients (86.6%), this measurement was obtained before the administrative admission timestamp, mostly in the emergency department. In 121 patients (1.1%), it preceded admission by more than 24 h so that the 24 h window fell entirely before the index hospitalization; these patients were retained in the primary analysis and excluded in a sensitivity analysis. The 24 h window was the primary window; the 48 h and 72 h windows were used in supplementary and sensitivity analyses. Throughout the paper, “24 h” refers to the 24 h after the first glucose measurement, not to the first 24 h of hospitalization. Glucose results reported by the laboratory as outside the analyzer’s measuring range (31 results in 30 patients, for example, “<5 mg/dL” [0.3 mmol/L] or “>800 mg/dL” [44.4 mmol/L]) could not be quantified; they were counted in the number of measurements but treated as missing for the other glycemic metrics and were quantified at half the lower limit or at the upper limit in a sensitivity analysis.

This anchor was chosen because patients with sepsis are evaluated in the emergency department before formal admission, where glucose is part of the standard biochemistry panel obtained from all patients, irrespective of presumed severity; a window beginning at the administrative admission time would discard these measurements. The first glucose measurement does not represent the biological onset of sepsis or of the stress response, and some measurements may precede the clinical recognition of sepsis. The interval between admission and the first glucose measurement was therefore described and compared between survivors and non-survivors and between patients with and without diabetes, and the principal models were repeated with windows anchored to hospital admission (Section 2.9.7).

### 2.6. Glycemic Variables

Within each window, we derived the first glucose value; the minimum, maximum, mean and median glucose values; and the number of glucose measurements. Glycemic variability was characterized by the standard deviation, the coefficient of variation (CV; standard deviation divided by mean glucose) and the glycemic range (maximum minus minimum). Because the standard deviation is undefined for a single value, CV could be calculated only in patients with at least two measurements within the window. CV was expressed as a proportion and, in regression models, scaled so that odds ratios refer to a 0.10 increase (10 percentage points). Glucose was sampled at clinical discretion, with a median of two measurements in the first 24 h. With two values, CV is a normalized difference between two observations rather than a description of temporal glucose instability, and it is sensitive to the interval between the two measurements; CV, in this study, is therefore the early glycemic variability estimated from routine intermittent measurements; it does not capture the excursions between samples and is not equivalent to variability from continuous or high-frequency monitoring. “Early glycemic variability” and “CV” are used in this sense throughout. Glycemic events were defined with clinically relevant thresholds: hypoglycemia as at least one value < 3.9 mmol/L (70 mg/dL), severe hypoglycemia as at least one value < 3.0 mmol/L (54 mg/dL), and hyperglycemia as at least one value > 10.0 mmol/L (180 mg/dL). Glucose values were converted from mg/dL to mmol/L by dividing by 18.0 and are reported in mmol/L throughout the main text. Odds ratios for time-weighted mean glucose are expressed per 1 mmol/L in the main text and per 1 mg/dL, as fitted, in the Supplementary Materials.

Time-weighted mean glucose (TWM) was calculated to account for unequal spacing between measurements: each value was weighted by the interval until the next measurement, the last value was carried forward to the end of the window, and intervals were truncated at the window boundary (derivation in Supplementary Methods S1).

### 2.7. Bias and Temporal Assumptions

Foreseeable sources of bias were addressed by common temporal anchors (Section 2.1 and Section 2.5), by confining in-hospital variables with uncertain timing to sensitivity analyses (Section 2.4), and by including the number of glucose measurements in every model with glycemic variables because variability metrics depend on measurement intensity. Missing CV was treated as informative (Section 2.9.5).

### 2.8. Study Size

No formal sample size calculation was performed because all eligible consecutive sepsis admissions in the study period were analyzed. The final cohort included 10,832 patients, 3055 with pre-existing diabetes and 7777 without documented diabetes, and 5803 deaths. Differences in analytic sample size across models reflect variable availability, mainly for CV-based analyses; the number of patients and deaths in each principal model is given in Table S36.

### 2.9. Statistical Analysis

The analyses were organized in four levels. The primary analyses were the association of pre-existing diabetes with in-hospital mortality (Model 2) and the diabetes-by-CV-24 h interaction in the primary 24 h model, which corresponds to the principal hypothesis. Secondary analyses comprised the diabetes-stratified CV associations; the independent associations of each glycemic parameter; the interactions of diabetes with TWM, hypoglycemia and hyperglycemia; and the replication of all models in the 48 h and 72 h windows; these were regarded as supportive. Sensitivity analyses assessed the robustness of the principal findings (Section 2.9.5 and Section 2.9.7). The spline models and the contrast subgroup of patients coded for sepsis without organ dysfunction were exploratory. No statistical analysis plan was registered before the analyses; this hierarchy follows the hypothesis stated in the Introduction.

#### 2.9.1. Descriptive Analyses

Continuous variables are summarized using the median and interquartile range (IQR), and categorical variables as shown using counts and percentages. Baseline and glycemic characteristics were compared between patients with and without diabetes and between survivors and non-survivors using the Mann–Whitney U test and the Chi-square test. All tests were two-sided; the two primary tests were evaluated at a significance level of 0.05 without adjustment. Because chance findings matter most among the interaction tests, the family of twelve interaction tests (four glycemic parameters by diabetes in each of three windows) was additionally adjusted using Holm’s method and a Benjamini–Hochberg false-discovery-rate control. No adjustment was applied to the other secondary, sensitivity or exploratory analyses, which were interpreted according to the consistency and direction of the estimates rather than using individual p values. Analyses were performed with jamovi version 2.7.13 (The jamovi project, Sydney, Australia; https://www.jamovi.org accessed on 23 April 2025) and Python 3.13 (statsmodels, SciPy, scikit-learn).

#### 2.9.2. Multivariable Models

Binary logistic regression was used to estimate odds ratios (ORs) with 95% confidence intervals (CIs) for in-hospital mortality. Five sequential models evaluated pre-existing diabetes: Model 1, unadjusted; Model 2, the primary adjusted model, including the baseline covariates (Section 2.4); Model 3, Model 2 plus the early glycemic variables of the first 24 h (hypoglycemia < 3.9 mmol/L, TWM-24 h and CV-24 h) and the number of glucose measurements; and Models 4 and 5, sensitivity versions of Models 2 and 3, additionally including mechanical ventilation, renal replacement therapy and surgical intervention. Age and calendar year were entered as continuous variables, assuming a linear relationship with the log-odds of death, with sex, comorbidities, infection foci and in-hospital interventions as binary variables.

To limit multicollinearity among correlated glycemic markers, separate models evaluated TWM, CV, hypoglycemia < 3.9 mmol/L, and hyperglycemia > 10.0 mmol/L individually, each adjusted for diabetes status, the baseline covariates, and the number of glucose measurements within the corresponding window.

Within the causal framework of Figure 2, Models 1 and 2 estimates the association of diabetes with death and are the basis of the primary conclusions. Model 3 conditions on early glycemic variables that are downstream of diabetes and therefore, estimates an association conditional on the early glycemic response rather than a total effect. Models 4 and 5 condition on post-baseline variables and are subject to over-adjustment. A minimal model, adjusted only for age, sex and calendar year, and additional models with intermediate adjustment sets were fitted as sensitivity analyses (Table S34).

#### 2.9.3. Effect Modification, Stratified Analyses, and Absolute Measures

Effect modification by pre-existing diabetes was assessed on the multiplicative scale by adding a diabetes-by-parameter interaction term to separate adjusted models for TWM, CV, hypoglycemia < 3.9 mmol/L, and hyperglycemia > 10.0 mmol/L, each including the baseline covariates and the number of glucose measurements; the interaction OR is the ratio of the parameter’s OR in patients with diabetes to that in patients without diabetes. The same models were also fitted separately in patients with and without diabetes (Supplementary Methods S2).

Because in-hospital mortality exceeded 50%, odds ratios overstate relative risks. Absolute measures were therefore estimated by g-computation with bootstrap confidence intervals (500 resamples): for diabetes, the standardized mortality under diabetes and under no diabetes from Model 2, with the corresponding risk difference and risk ratio, in the full cohort and among patients alive 24 h and 48 h after the first glucose measurement, was employed; for CV-24 h, standardized mortality, marginal risk differences and risk ratios across CV-24 h values from the primary interaction model, with effect modification on the additive scale as the difference in risk differences between strata, was used. Risk ratios were also estimated using modified Poisson regression with robust standard errors (Supplementary Methods S2).

#### 2.9.4. Assessment of Non-Linearity

Non-linearity of the association between CV-24 h and in-hospital mortality was explored separately by diabetes status, with restricted cubic splines adjusted for the covariates of the primary interaction model (four knots at the 5th, 35th, 65th and 95th percentiles; tests and sensitivity analyses results are shown in Supplementary Methods S2).

#### 2.9.5. Informative Observation and Survivor Bias

Because CV requires at least two measurements within the window, its availability is a post-baseline condition that depends on the patient surviving long enough to be re-measured and on monitoring intensity. CV analyses are therefore conditional on survival to a second measurement; they are not complete-case analyses under a missing-at-random assumption. This informative observation was examined by (i) mortality according to the number of glucose measurements within 24 h; (ii) the timing of the second measurement; (iii) the availability of CV-24 h among early deaths, later deaths and survivors; (iv) landmark analyses; (v) restriction to patients with at least three measurements; and (vi) inverse-probability-of-observation weighting (Supplementary Methods S2). Each landmark analysis was restricted to patients alive and hospitalized at 24, 48 or 72 h after the first glucose measurement and estimates the association in patients who have survived to that time; in the strict landmarks at 48 h and 72 h, the exposure window was completely observed before the landmark. Because weighting on measured characteristics cannot correct selection through survival itself, the landmark analyses were regarded as the principal robustness test of the effect-modification finding.

#### 2.9.6. Missing Data

Each model used the patients with complete data for its variables. Missingness was minimal, except in regards to CV. In the primary 24 h analysis, CV-24 h could be computed in 6450 patients (59.5%) and was unavailable in 4382 (40.5%), of whom 4377 had a single measurement within 24 h; TWM-24 h was unavailable in 30 patients because of a glucose result outside the measuring range. Models including both CV-24 h and TWM-24 h (Models 3 and 5) were therefore fitted in 6434 patients. The characteristics of patients with and without available CV-24 h were compared, and CV availability was handled as described in Section 2.9.5.

#### 2.9.7. Sensitivity Analyses

Besides the models with in-hospital interventions (Section 2.9.2), all major glycemic analyses were replicated in the 48 h and 72 h windows. Further sensitivity analyses of the principal CV models comprised (Supplementary Methods S2): (i) exclusion of the 121 patients whose first glucose measurement preceded admission by more than 24 h; (ii) restriction by the number of measurements within each window; (iii) glycemic metrics recomputed in windows anchored to the administrative admission timestamp; (iv) restriction according to the sepsis ascertainment route, coded organ dysfunction and septic shock, with patients coded for sepsis without organ dysfunction as a contrast group; (v) additional adjustment for the number of organ systems with coded acute dysfunction; (vi) stratification by mechanical ventilation as a proxy for intensive care unit (ICU) treatment because ICU admission was not reliably recorded; (vii) stratification by calendar period (2011–2015, 2016–2019 and 2020–2024); (viii) checks of measurement timing and of extreme glucose values; and (ix) quantification of the glucose results reported outside the measurement range (Section 2.5).

#### 2.9.8. Incremental Predictive Performance

Whether CV-24 h improved the prediction of in-hospital mortality beyond the Model 2 covariates and the number of glucose measurements was assessed separately in patients with and without diabetes using the likelihood-ratio test, discrimination (optimism-corrected C-statistic), calibration, the Brier score and reclassification indices (Supplementary Methods S2).

### 2.10. Ethical Considerations

The study was approved by the Ethics Committee of the Carol Davila University of Medicine and Pharmacy (approval No. 6098, 21 March 2025) and by the Ethics Committee of the University Emergency Hospital of Bucharest (approval No. 36964, 17 June 2022). Given the retrospective design and the use of routinely collected, de-identified data, the requirement for informed consent was waived. The study was conducted in accordance with the Declaration of Helsinki [21].

## 3. Results

### 3.1. Cohort Characteristics and Documented Infection Foci

The cohort comprised 10,832 patients with sepsis, 7777 without documented diabetes and 3055 with pre-existing diabetes (Table 1). Age, sex, cirrhosis, Clostridioides difficile infection, renal replacement therapy and the number of organ systems with coded acute dysfunction were similar between groups. Patients with diabetes had higher first glucose values, a longer hospital stay, more mechanical ventilation, and more CKD, COPD and coded acute kidney injury; malignancy, peritonitis, pneumonia, coded shock and surgical intervention were less frequent. In-hospital mortality was 51.0% with and 54.6% without diabetes (*p* < 0.001). Among the 3055 patients with diabetes, 2961 (96.9%) carried a type 2 code, 121 (4.0%) a type 1 code, and 92 (3.0%) a code for other or unspecified diabetes, some carrying more than one; 2750 (90.0%) carried at least one code for a diabetes complication. Codes for hyperglycemia without diabetes (R73.x) were recorded in 33 patients, 29 of whom had no diabetes code and were classified as not having diabetes.

The first glucose measurement preceded the administrative admission timestamp in 9380 patients (86.6%). Across the cohort, a median of 3.8 h was obtained before admission (IQR 6.7 to 1.7 h before) and fell between 24 h before and 24 h after admission in 97.5% of patients. The interval differed little between non-survivors and survivors (median 4.1 vs. 3.6 h before admission; *p* < 0.001) or between patients with and without diabetes (4.1 vs. 3.7 h; *p* < 0.001) (Table S27).

Sepsis coding was predominantly non-specific with respect to the causative organism. Of 10,698 sepsis-related ICD-10-AM codes, 6534 denoted other specified sepsis and 3127 unspecified sepsis; 1037 (9.7%) identified an organism, most often *Escherichia coli* (n = 248) and *Staphylococcus aureus* (n = 178), with Gram-negative organisms predominating (Table S1). Clinically documented foci were more informative, i.e., pneumonia in 2176 patients (20.1%), peritonitis in 742 (6.9%) and C. difficile infection in 620 (5.7%).

In-hospital mortality was 53.6% (5803/10,832). Non-survivors were older and more often had codes for mechanical ventilation, malignancy, CKD, COPD and pneumonia; surgical intervention, peritonitis and diabetes (26.8% vs. 29.8%; *p* < 0.001) were more frequent among survivors (Table 2).

### 3.2. Early Glycemic Profile According to Diabetes Status

Early glycemic profiles differed by diabetes status in all windows (Table 3; Tables S3 and S4). In the 24 h window, patients with diabetes exhibited higher first, mean, minimum, maximum and time-weighted mean glucose levels and higher variability (median CV-24 h 0.229 vs. 0.193); hyperglycemia > 10.0 mmol/L was more frequent (57.2% vs. 19.6%), hypoglycemia < 3.9 mmol/L was slightly less frequent (8.6% vs. 10.5%), and the number of results of severe hypoglycemia < 3.0 mmol/L did not differ. The same pattern held at 48 h (median CV 0.264 vs. 0.217) and 72 h (0.281 vs. 0.231). The number of measurements increased with longer windows in both groups, and the proportion of patients with missing CV decreased from 24 to 48 and 72 h. The distribution of the number of measurements within 24 h by diabetes status is given in Table S23: one measurement in 42.7% of patients without and 34.6% of patients with diabetes, two in 41.2% and 46.2%, three in 13.1% and 14.8%, and four or more in 3.0% and 4.4%.

### 3.3. Pre-Existing Diabetes and In-Hospital Mortality

Diabetes was associated with lower odds of in-hospital death in the unadjusted model (OR, 0.866; 95% CI, 0.796–0.942) and after adjustment for baseline covariates (Model 2: OR, 0.822; 95% CI, 0.751–0.899; *p* < 0.001) (Table 4; full output in Table S5). The estimate was similar with adjustment for age, sex and calendar year only (OR, 0.813; 95% CI, 0.746–0.887) and with intermediate adjustment sets (Table S34), and it was further from unity in the sensitivity model including in-hospital interventions (Model 4), which is subject to over-adjustment. In the 6434 patients with CV-24 h and TWM-24 h, diabetes remained associated with lower odds of death after additional adjustment for early glycemic parameters (Models 3 and 5, OR 0.69 and 0.68). Models 3 and 5 were estimated in a smaller sample that differed from the full cohort (Section 3.4) and conditioned on variables that may lie between diabetes and death, so their estimates are not directly comparable with those for Models 2 and 4. The same pattern was observed in the 48 h and 72 h windows (Tables S6 and S7).

On the absolute scale, the association was modest. Standardized in-hospital mortality from Model 2 was 54.8% (95% CI 53.8–56.0) without and 50.5% (48.9–52.3) with diabetes, with a risk difference of −4.3 percentage points (95% CI −6.3 to −2.3) and a risk ratio of 0.921 (0.887–0.958); modified Poisson regression gave a risk ratio of 0.935 (0.900–0.973) (Table S40).

Diabetes was recorded less often in patients who died within 24 h of admission (22.3%) than in those who died later (25.6–30.7%) or survived (29.8%), and these early deaths carried fewer coded secondary diagnoses (median 8 vs. 9–11) (Table S30). The lower adjusted odds of death in patients with diabetes persisted after exclusion of deaths within 24 h of admission (OR, 0.899; 95% CI, 0.816–0.990) and among patients alive 24 h and 48 h after the first glucose measurement (OR, 0.879; 95% CI, 0.799–0.966; and OR, 0.900; 95% CI, 0.814–0.994), but the absolute difference narrowed: the standardized risk difference was −2.8 percentage points (95% CI −4.9 to −0.6) at 24 h and −2.3 (−4.5 to −0.1) at 48 h, with modified Poisson risk ratios of 0.956 (0.914–1.000) and 0.963 (0.916–1.013) (Table S40). After exclusion of deaths within 48 h of admission, the odds ratio was 0.921 (95% CI 0.833–1.018). Additional adjustment for the number of coded secondary diagnoses moved the full-cohort estimate further from unity (OR, 0.721; 95% CI, 0.658–0.791; Table S30).

### 3.4. Early Glycemic Parameters and In-Hospital Mortality

**Figure 3:**
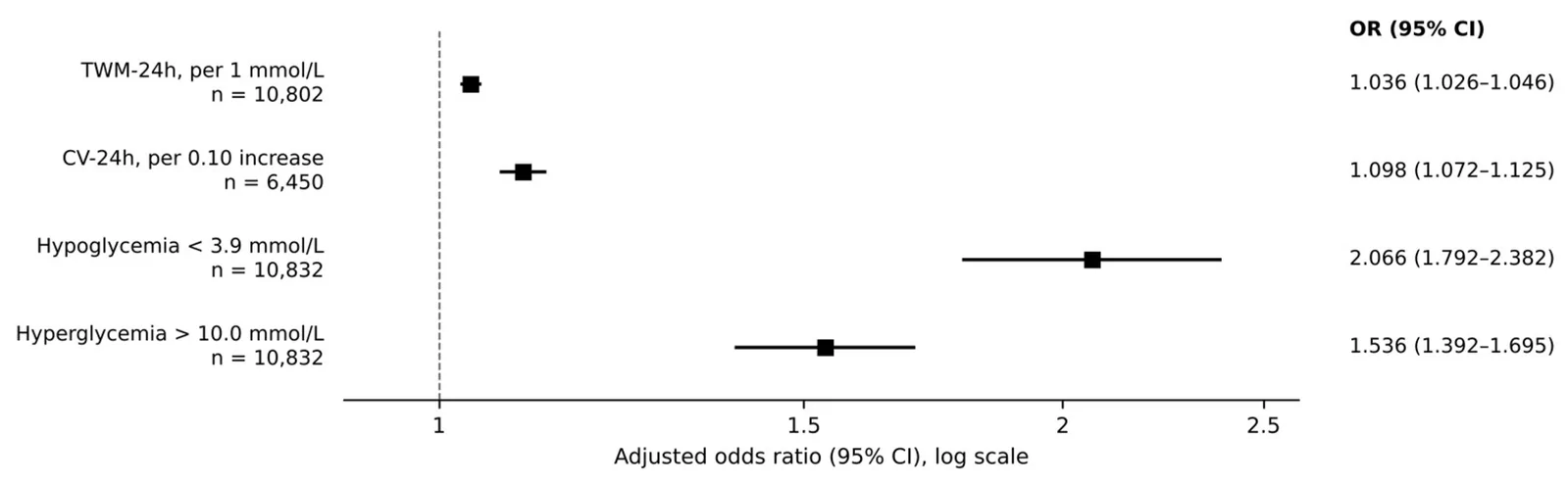
Independent associations between early glycemic parameters and in-hospital mortality. Points are adjusted odds ratios, and bars show 95% confidence intervals for TWM-24 h, CV-24 h, hypoglycemia < 3.9 mmol/L (70 mg/dL) and hyperglycemia > 10.0 mmol/L (180 mg/dL), from separate logistic models adjusted for diabetes status, calendar year, age, sex, malignancy, pneumonia, cirrhosis, chronic kidney disease, chronic obstructive pulmonary disease, peritonitis and number of glucose measurements within 24 h. Odds ratios are per 1 mmol/L for TWM-24 h and per 0.10 increase for CV-24 h (per 1 mg/dL in Table S8); the x-axis is logarithmic. The four metrics are on different scales, so their odds ratios are not comparable in magnitude. CV, coefficient of variation; TWM, time-weighted mean glucose.

In separate adjusted models, each early glycemic parameter of the 24 h window was independently associated with in-hospital mortality (Figure 3; Table S8): OR 1.098 (95% CI 1.072–1.125) per 0.10 increase in CV-24 h, 2.066 (1.792–2.382) for hypoglycemia < 3.9 mmol/L, 1.536 (1.392–1.695) for hyperglycemia > 10.0 mmol/L, and 1.036 (1.026–1.046) per 1 mmol/L increase in TWM-24 h. Because these metrics are on different scales, their ORs are not comparable in magnitude. Patients with available CV-24 h differed from those without: they more often had diabetes (30.9% vs. 24.2%), required mechanical ventilation (28.4% vs. 11.2%), and had more glucose measurements, whereas crude mortality was similar (54.0% vs. 53.0%) (Table S9). This similarity conceals strong selection by early survival (Section 3.7). Diabetes remained associated with lower mortality in each of these models; the same pattern held in the 24 h sensitivity analyses and in the 48 h and 72 h windows (Tables S10–S14).

### 3.5. Effect Modification by Pre-Existing Diabetes

Pre-existing diabetes modified the associations of TWM-24 h (interaction OR per 1 mmol/L, 0.963; 95% CI, 0.943–0.984; *p* < 0.001), CV-24 h (interaction OR per 0.10 increase, 0.912; 95% CI, 0.868–0.959; *p* < 0.001) and hyperglycemia (OR, 0.722; 95% CI, 0.594–0.878; *p* = 0.001); the interaction for hypoglycemia was not statistically significant (OR, 0.766; 95% CI, 0.557–1.052; *p* = 0.099) (Table 5, Panel A). In each case, the association with death was weaker in patients with diabetes. The three significant interactions remained significant after Holm adjustment across the twelve interaction tests (adjusted *p* = 0.005, 0.003 and 0.009; Table S33). After additional adjustment for in-hospital interventions, the CV interaction remained significant (OR, 0.936; 95% CI, 0.887–0.987; *p* = 0.015), whereas those for TWM (*p* = 0.053) and hyperglycemia (*p* = 0.059) were borderline (Table S15). In the 48 h and 72 h windows, the interactions for CV (OR, 0.930; 95% CI, 0.887–0.975; and OR, 0.929; 95% CI, 0.885–0.975; Holm-adjusted *p* = 0.019 for both) and TWM (Holm-adjusted *p* = 0.003 and 0.001) remained significant after multiplicity adjustment; that for hyperglycemia was nominally significant (*p* = 0.019 and 0.016; Holm-adjusted *p* = 0.078 for both), and that for hypoglycemia was not (*p* = 0.425 and 0.478) (Tables S16 and S18). In the corresponding sensitivity models, the CV interaction was attenuated (*p* = 0.059 and 0.074), and that for TWM remained nominally significant (*p* = 0.031 and 0.019) (Tables S17 and S19).

In the stratified analyses (Table 5, Panel B), each 0.10 increase in CV-24 h was associated with higher odds of death in patients without diabetes (OR, 1.137; 95% CI, 1.102–1.173), whereas in patients with diabetes, the association was weaker and not statistically significant (OR, 1.034; 95% CI, 0.994–1.076; *p* = 0.099), with a confidence interval that does not exclude a modest association; the sensitivity models gave similar estimates. At 48 h and 72 h, the pattern was the same (without diabetes OR, 1.131; 95% CI, 1.100–1.164, and OR, 1.145; 95% CI, 1.114–1.177; with diabetes OR, 1.043; 95% CI, 1.003–1.085; *p* = 0.033, and OR, 1.053; 95% CI, 1.011–1.097; *p* = 0.012) (Tables S20 and S21); in the longer windows, the association in patients with diabetes was small but statistically significant.

On the absolute scale, standardized in-hospital mortality rose from 51.3% to 62.5% across CV-24 h values of 0.10 to 0.50 in patients without diabetes and from 49.2% to 52.5% in patients with diabetes (Table 5, Panel C). Each 0.10 increase in CV-24 h corresponded to a marginal risk difference of 2.79 percentage points (95% CI 2.09–3.49) in patients without diabetes and 0.82 (−0.13 to 1.77) in patients with diabetes, a difference between strata of 1.97 percentage points (95% CI 0.83–3.08), indicating effect modification on the additive as well as the multiplicative scale; modified Poisson regression gave concordant results (ratio of risk ratios, 0.970; 95% CI, 0.952–0.989; *p* = 0.002) (Table S31).

### 3.6. Shape of the Association Between Early Glycemic Variability and Mortality

**Figure 4:**
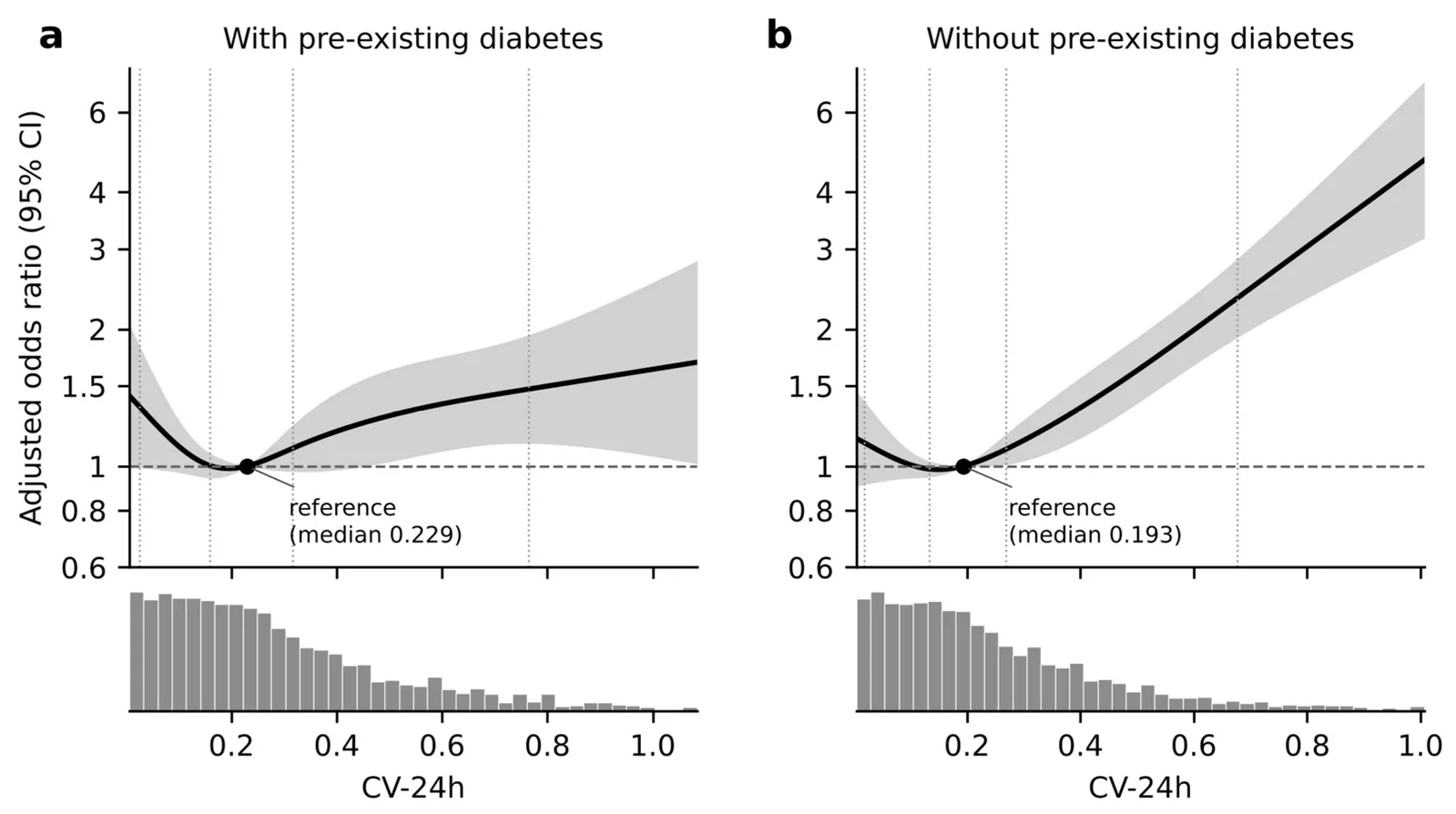
Exploratory restricted cubic spline analysis of the association between early glycemic variability (CV-24 h) and in-hospital mortality according to diabetes status. (**a**) Patients with pre-existing diabetes. (**b**) Patients without pre-existing diabetes. Curves show adjusted odds ratios relative to the stratum-specific median CV-24 h (0.229 with and 0.193 without diabetes), with 95% confidence bands, from logistic models with four knots at the 5th, 35th, 65th and 95th percentiles of the stratum-specific distribution (0.025, 0.158, 0.316 and 0.764 with diabetes; 0.018, 0.133, 0.269 and 0.677 without diabetes), adjusted for calendar year, age, sex, malignancy, pneumonia, cirrhosis, chronic kidney disease, chronic obstructive pulmonary disease, peritonitis and number of glucose measurements within 24 h. The distribution of CV-24 h is shown below the x-axis, and the range is truncated at the 1st and 99th percentiles. CV, coefficient of variation.

In exploratory spline analyses (Figure 4, Table S32), the association between CV-24 h and mortality was non-linear in patients without diabetes (likelihood-ratio test *p* = 0.0003 with four knots; *p* = 0.0001 and 0.0004 with three and five knots). In patients with diabetes, the evidence of non-linearity was borderline (*p* = 0.052 with four knots; 0.063 and 0.049 with three and five knots), and the overall association was significant (*p* = 0.034); the shape differed between strata (*p* < 0.001). In patients without diabetes, the odds of death relative to the median CV-24 h (0.193) did not differ below the median (OR, 1.093; 95% CI, 0.920–1.297 at the 10th percentile) and rose above it (OR, 1.342; 95% CI, 1.151–1.565 at 0.40; OR, 2.338; 95% CI, 1.915–2.855 at the 95th percentile), with wide confidence intervals at the upper extreme. The non-linearity was confined to patients with exactly two measurements (*p* < 0.001) and absent in those with three or more (*p* = 0.393 and 0.701 with three and four knots), whereas the linear association was similar in both groups (OR per 0.10 increase, 1.132; 95% CI, 1.091–1.173, and OR, 1.154; 95% CI, 1.086–1.226). Among patients without diabetes with two measurements, hypoglycemia was present in 43.2% of those with CV-24 h above 0.45 compared with 9.2% of those with CV-24 h of 0.05–0.45. The curvature therefore reflects the properties of two-point CV estimates rather than a threshold in the underlying relationship.

### 3.7. Informative Observation and Robustness of the Effect-Modification Finding

In-hospital mortality did not vary materially with the number of glucose measurements in the 24 h window (53.0%, 53.8%, 55.1% and 51.8% with one, two, three and four or more measurements; adjusted OR per additional measurement, 1.038; 95% CI, 0.988–1.091; Table S23). A second glucose measurement was obtained at any time in 9229 patients (85.2%), a median of 17.0 h (IQR 10.3–30.1) after the first; 7.9%, 27.0%, 45.6% and 59.5% of the cohort had a second value within 6, 12, 18 and 24 h (Table S24). Availability of CV-24 h depended strongly on early survival: among the 1450 patients who died within 24 h of admission (25.0% of all deaths), CV-24 h was computable in 32.1%, and 66.9% never had a second measurement, whereas CV-24 h was available in 75.9% of patients who died at 24–48 h, 70.9% of those who died at 2–7 days, 66.5% of those who died later, and 59.0% of survivors (Table S24). In total, 1289 patients (11.9%) left the hospital, 1216 by death, less than 24 h after the first glucose measurement.

The diabetes-by-CV interaction was robust to inverse-probability-of-observation weighting (OR per 0.10 increase: 0.913 and 0.918 with the two propensity models) (Table 6). The landmark analyses progressively removed the earliest deaths from the 6450 patients with CV-24 h (3481 deaths): 327 patients (310 deaths) had left the hospital before 24 h, 911 (844 deaths) before 48 h and 1267 (1136 deaths) before 72 h (Table S25). Among patients alive at 24 h, the interaction persisted (OR, 0.938; 95% CI, 0.891–0.988; *p* = 0.016), and CV-24 h remained associated with death in patients without diabetes (OR 1.092) but not in those with diabetes (OR 1.023); the interaction also persisted when combined with weighting (OR 0.940) but was attenuated and no longer statistically significant after additional adjustment for in-hospital interventions (OR, 0.963; 95% CI, 0.911–1.018; *p* = 0.180). Among patients alive at 48 h or 72 h, the interaction was attenuated and not statistically significant (OR 0.974–0.987); the association in patients without diabetes weakened (OR 1.042–1.055) but remained significant, and estimates in patients with diabetes were similar (OR 1.017–1.028) (Table 6, Table S25). Exclusion of the 121 patients whose first glucose measurement preceded admission by more than 24 h did not alter the estimates (interaction OR 0.911). The association between early glycemic variability and death in patients without diabetes was therefore concentrated in deaths within the first days of hospitalization; among patients who survived those days, the two strata differed little.

In patients without diabetes, CV remained associated with death in every stratum defined by a minimum of three, four or five measurements within 24, 48 or 72 h (OR per 0.10 increase, 1.092–1.232), and the association did not depend on the number of measurements (CV-by-count interaction *p* = 0.389) (Table S26). In patients with diabetes, the estimates ranged from 0.986 to 1.063 and were statistically significant only with at least three measurements within 72 h (OR, 1.056; 95% CI, 1.004–1.111). The interaction remained below 1 in every stratum (OR 0.823–0.955) but was less precisely estimated: it was statistically significant with at least three measurements within 48 h (OR, 0.915; 95% CI, 0.858–0.976) and not in the other strata, including at least three measurements within 24 h (OR, 0.911; 95% CI, 0.825–1.006; *p* = 0.065).

With windows anchored to the admission timestamp, the interaction was reproduced from admission to 24 h after (OR, 0.918; 95% CI, 0.847–0.995; *p* = 0.037) and from 24 h before to 24 h after admission (OR, 0.920; 95% CI, 0.877–0.966; *p* < 0.001), but not from admission to 48 h after (OR, 0.957; 95% CI, 0.902–1.014; *p* = 0.137) (Table S27). Because the strict admission-anchored window discards emergency-department measurements, 28.9% of patients had no glucose value, and only 22.2% had a computable CV within 24 h of admission.

The interaction was reproduced in patients with any explicit sepsis code (OR, 0.911; 95% CI, 0.865–0.959; n = 10,380), with a sepsis code as principal diagnosis (OR, 0.896; 95% CI, 0.830–0.967; n = 4360), with a sepsis code and coded organ dysfunction (OR, 0.899; 95% CI, 0.843–0.959; n = 6140; mortality 69.1%) and with septic shock documented in the discharge diagnosis (OR, 0.809; 95% CI, 0.684–0.957; n = 1154; mortality 73.1%) (Table S28). In the subgroup with a sepsis code and organ dysfunction identified from diagnosis codes alone (subgroup C of Table S28), the primary findings were otherwise unchanged: diabetes OR, 0.828 (95% CI, 0.731–0.937) in the Model 2 covariate set, CV-24 h OR, 1.148 (1.102–1.195) without and 1.028 (0.977–1.082) with diabetes. In patients coded for sepsis without coded organ dysfunction (n = 4240; mortality 30.7%), CV-24 h was not associated with death in either stratum (OR, 1.048; 95% CI, 0.982–1.119 without and OR, 0.985; 95% CI 0.903–1.076 with diabetes). In-hospital mortality was 54.7%, 52.5% and 57.7% in patients identified by a principal sepsis code, by a secondary sepsis code only and without an explicit sepsis code.

Coded acute organ dysfunction was strongly related to mortality (27.6%, 59.5%, 77.3% and 85.9% with 0, 1, 2 and ≥3 affected systems) and to CV-24 h (median 0.169 to 0.238 in patients without and 0.212 to 0.265 in patients with diabetes) (Table S29). After adjustment for the number of affected systems, the association in patients without diabetes was attenuated but persisted (OR per 0.10 increase, 1.095; 95% CI, 1.059–1.132), the estimate in patients with diabetes was close to unity (OR, 1.006; 95% CI, 0.963–1.050), and the interaction persisted (OR, 0.924; 95% CI, 0.876–0.975; with in-hospital interventions also added, OR, 0.944; 95% CI, 0.893–0.999). The interaction was present in mechanically ventilated patients (OR, 0.877; 95% CI, 0.778–0.988) and of similar direction but not statistically significant in non-ventilated patients (OR, 0.947; 95% CI, 0.892–1.004), in whom CV-24 h was also associated with death in patients with diabetes (OR, 1.051; 95% CI, 1.002–1.103), with no evidence of heterogeneity between strata (*p* = 0.27; Table S37). Across calendar periods, the proportion of patients with sepsis as the principal diagnosis ranged from 29.3% to 49.2%, and that with coded organ dysfunction rose from 52.4% to 69.3%, whereas mortality changed little (54.5%, 54.1% and 52.8%); CV-24 h was associated with mortality in patients without diabetes in every period (OR 1.100–1.168), and the interaction did not differ between periods (*p* = 0.420; Table S38).

The estimates were not sensitive to irregular sampling: adjustment for the time span of the measurements or for the interval between the first two values, or restriction to patients whose second value was obtained at least 6 h after the first, left the interaction unchanged (OR 0.914–0.917) (Table S39). Adjustment for hypoglycemia and hyperglycemia, or exclusion of patients with any value below 3.0 mmol/L (54 mg/dL) or above 22.2 mmol/L (400 mg/dL), attenuated the association in patients without diabetes (OR 1.064 and 1.083), which nonetheless remained significant, as did the interaction (OR 0.930 in both analyses). After exclusion of all patients with a value below 3.9 mmol/L (70 mg/dL), CV-24 h remained associated with death in patients without diabetes (OR, 1.072; 95% CI, 1.030–1.116), whereas the interaction was not statistically significant (OR, 0.952; 95% CI, 0.893–1.015); among patients whose values never exceeded 10.0 mmol/L (180 mg/dL), the association in patients without diabetes was unchanged (OR, 1.127; 95% CI, 1.078–1.178; interaction OR, 0.921; 95% CI, 0.847–1.001). Quantifying the 31 glucose results reported outside the measuring range did not change the estimates (interaction OR, 0.912; 95% CI, 0.868–0.958).

### 3.8. Incremental Predictive Performance of the Data

In patients without diabetes, adding CV-24 h to the base model improved model fit (likelihood-ratio: χ^2^ = 69.75, *p* < 0.001) and reduced the Brier score from 0.2238 to 0.2203, but increased the C-statistic only from 0.672 to 0.685 (difference of 0.013; 95% CI, 0.008–0.019; optimism-corrected, 0.669 to 0.682). When the base model included coded organ dysfunction, the increase was 0.001 (0.823 to 0.825; 95% CI, 0.000–0.003), although the likelihood-ratio test remained significant (*p* < 0.001). Calibration was adequate with and without CV (optimism-corrected slope of 0.980 in both models). In patients with diabetes, CV-24 h did not materially improve fit or discrimination (likelihood-ratio: *p* = 0.098; C-statistic: 0.651 to 0.653) (Table S35).

## 4. Discussion

In a 14-year cohort of 10,832 consecutive adults hospitalized with sepsis, the prognostic weight of early glycemic variability differed according to pre-existing diabetes. Three findings emerge. First, patients with diabetes had a different early glycemic profile, with higher glucose exposure and greater variability. Second, diabetes was associated with modestly lower observed mortality in crude and adjusted analyses, i.e., about four percentage points in the full cohort and lower among patients surviving the first day; we do not interpret this as a protective effect. Third, early glycemic disturbances, including higher time-weighted mean glucose, hypoglycemia, and hyperglycemia, as well as greater variability, were each associated with in-hospital mortality, but their prognostic meaning was not uniform: diabetes attenuated the associations of mean glucose, hyperglycemia and variability, whereas the interaction for hypoglycemia was not statistically significant. The association between CV-24 h and mortality was consistent in patients without diabetes and weaker in those with diabetes across the 24, 48 and 72 h windows, and in patients without diabetes, it was concentrated in deaths within the first days of hospitalization. The study also contributes data from a large Eastern European cohort, a setting under-represented in a literature dominated by North American databases and Asian and Western European cohorts.

### 4.1. Differential Prognostic Meaning of Early Glycemic Variability

These results support the hypothesis that the prognostic meaning of early glycemic variability depends on diabetes status and extend the intensive care signals summarized in the Introduction [16,17,18] to a hospital-wide sepsis cohort. They agree with a large intensive care cohort in which the association between the glucose coefficient of variation and mortality was not observed in patients with diabetes and increased with the severity of sepsis [22], as well as with studies restricted to patients without diabetes, in which early variability was associated with mortality [23] in one small prospective cohort independently of APACHE II scores [24]. The effect modification was present on the multiplicative and the additive scale, with a moderate absolute gradient (about 2.8 vs. 0.8 percentage points of standardized mortality per 0.10 increase in CV-24 h). Measurement intensity and recorded severity are unlikely to explain the effect modification, which persisted after inverse-probability-of-observation weighting in the 48 h and 72 h windows among patients alive 24 h after the first measurement and across ascertainment definitions; coded organ dysfunction was distributed almost identically in the two groups, and the CV distributions overlapped widely.

The landmark analyses, however, define the scope of the finding. When deaths within the first 48–72 h were excluded, the association in patients without diabetes weakened, and the interaction was no longer significant. The excess risk is therefore concentrated in early deaths and plausibly reflects, in part, illness severity at the time of measurement. The interaction should not be read as a stable prognostic relationship that applies throughout the hospital stay; it describes short-term risk in patients measured at presentation. In patients with diabetes, the association was weaker rather than absent, i.e., it was not statistically significant at 24 h but small and significant at 48 h and 72 h, and the confidence intervals remain compatible with a modest adverse association. The spline analyses suggested a difference in shape between strata, but the curvature was confined to patients with two measurements and is exploratory.

The data do not identify a mechanism, and the associations are prognostic, not evidence of a direct effect of glycemic variability on outcome. Severe sepsis itself produces hepatic dysfunction, impaired gluconeogenesis, insulin resistance, altered nutritional intake, catecholamine release and organ failure, each of which can increase glycemic variability; consistently, variability increased with the number of organ systems with coded dysfunction, and adjustment for that count attenuated the association without abolishing it. Early variability may also reflect treatment rather than disease: insulin, corticosteroids, dextrose-containing infusions and nutrition alter glucose and are prescribed according to clinical condition, and none of these could be examined. Patients with known diabetes are usually monitored and treated more actively, so that part of their early variability is likely iatrogenic, arising from insulin titration; monitoring intensity itself did not account for the findings. Within these limits, two hypotheses merit prospective testing. First, the prognostic weight of a glucose value may depend on its departure from habitual glycemia, the principle underlying the stress hyperglycemia ratio [12]: high variability departs sharply from normal regulation in a patient without diabetes but lies closer to the usual range in a patient with diabetes. Second, repeated exposure to glycemic variability might induce an adaptation that limits the injury associated with acute glycemic disturbance [13]; this is speculative, rests on an analogy with ischemic preconditioning, and could not be examined here because habitual glycemia was not measured.

The design addresses the three limitations of earlier work, as outlined in the Introduction. Exposure was restricted to the first 24–72 h after the first glucose value rather than reflected over the entire intensive care stay and was adjusted for measurement intensity and, in sensitivity analyses, for major in-hospital interventions; the signal was already detectable on the first day. Effect modification was tested formally as an interaction derived from the study hypothesis rather than inferred from selected subgroups. The cohort was not restricted to intensive care. In line with trajectory-based approaches [25], these results indicate that prognostic information lies in early glycemic variability, even when estimated from the sparse measurements of routine care, and not only in glucose level; whether the finer dynamics captured by continuous monitoring carry additional information cannot be inferred from these data.

### 4.2. Hypoglycemia, Hyperglycemia, and Mean Glucose

Early hypoglycemia was associated with higher odds of in-hospital death in all models and windows (OR of 2.07 at 24 h and 1.66–1.74 at 48 h and 72 h), in line with a meta-analysis reporting higher in-hospital and short-term mortality (pooled OR, 1.40; 95% CI, 1.18–1.65) in septic patients with hypoglycemia [4]. The larger estimate in our cohort may reflect the exposure definition, since we captured any hypoglycemic value during the first 24 h rather than at admission glucose alone. In sepsis, hypoglycemia may result from hepatic dysfunction, depleted glycogen stores and impaired gluconeogenesis, or from insulin therapy, and may therefore, mark advanced disease or treatment intensity rather than contribute to death; without medication data, these cannot be distinguished. The interaction with diabetes did not reach significance in any window (*p* = 0.099 at 24 h), but its direction, a weaker association in patients with diabetes, agrees with the subgroup finding of the same meta-analysis [4]. Our data neither confirm nor exclude a diabetes-specific difference for hypoglycemia.

Diabetes did modify the associations of glucose level. The interactions with TWM and hyperglycemia were significant at 24 h after correction for multiple testing, as well as at 48 h and 72 h, although both were attenuated after adjustment for in-hospital interventions. Hyperglycemia and higher mean glucose therefore carried less prognostic weight in patients with diabetes, consistent with the stress hyperglycemia ratio [12] and with an emergency-department cohort in which admission hyperglycemia was associated with higher mortality in patients without diabetes than in those with diabetes at the same glucose level [26]. Effect modification by diabetes thus extended to both the level and the variability of early glucose. The variability interaction, as the effect most extensively tested, was consistent in direction across the sensitivity analyses of Section 3.7, although less precisely estimated in the smaller subgroups.

### 4.3. Pre-Existing Diabetes and Sepsis Mortality

Diabetes was not associated with excess in-hospital mortality, in line with studies reporting no excess risk in sepsis [27,28,29], whereas each early glycemic disturbance revealed such an association. In a recent single-center study, patients with diabetes had higher crude mortality (25.8% vs. 15.2%) [10], but the prognostic model was restricted to the diabetic subgroup. The opposite conclusions of the two recent meta-analyses [3,15] are best understood through their design (Table S22). The 2022 review pooled 21 studies of sepsis of varying severity, with in-hospital and later mortality endpoints and moderate-to-high heterogeneity; diabetes was associated with acute renal failure but not with death. The 2026 review pooled 13 intensive care studies with very high heterogeneity and evidence of publication bias, and its excess risk was confined to the in-hospital endpoint. The discrepancy most likely reflects differences in study selection, populations and endpoints rather than a biological contradiction, and supports the view that the diabetes–mortality relationship in sepsis is heterogeneous and context-dependent.

After adjustment, diabetes was associated with lower observed odds of in-hospital death, and the association persisted among patients surviving the first day. On the risk scale, however, the association was small, i.e., about four percentage points of standardized mortality in the full cohort, less than three among patients alive at 24 h, and no longer statistically significant with modified Poisson regression among those alive at 48 h. This should not be interpreted as a protective effect of diabetes. A similar adjusted odds ratio (0.83) was reported in an emergency-department sepsis cohort, in which patients with diabetes also had lower severity scores [26]. Several non-causal explanations are possible. Clinicians may have a lower threshold for admitting patients with diabetes, who would then enter the cohort earlier or at a less severe stage; patients with known diabetes are under closer glucose surveillance; and preadmission glucose-lowering treatment, including metformin [14], may contribute to this result. Differential coding was also considered: diabetes was recorded less often in patients who died within 24 h of admission, but excluding these patients only partly attenuated the association, and adjustment for the number of coded diagnoses strengthened it so that incomplete coding of early deaths does not explain it. Residual confounding by severity not captured in coded organ dysfunction cannot be excluded, and none of these explanations could be examined directly.

### 4.4. Casemix, Infection Foci, and Generalizability

In-hospital mortality in our cohort (53.6%) is roughly twice the pooled estimate for hospital-treated sepsis and closer to figures reported for intensive care populations and septic shock, in which a crude hospital mortality of 52% has been described [1,2]. Three features of the setting are relevant. The hospital is a tertiary emergency referral center receiving transfers of patients with established organ failure; a quarter of non-survivors died within one day of admission, 62% of the cohort had a coded acute organ dysfunction (Table 1), and mortality was 69.1% in patients with a sepsis code and organ dysfunction observed from diagnosis codes alone compared with 30.7% in those without (Table S28). Ascertainment depended on discharge coding, which is less sensitive than clinical criteria and preferentially captures more severe, better-documented episodes [30,31]; such selection enriches the cohort for fatal cases. Mortality also varies widely with the source of infection [2]: pneumonia was twice as frequent among non-survivors as among survivors, whereas peritonitis was more frequent among survivors, plausibly reflecting the availability of source control for abdominal sepsis at a hospital with a large surgical caseload. These considerations limit extrapolation of the absolute mortality rate. They do not undermine the internal comparisons, which were reproduced in every subgroup defined by explicit or stringent sepsis coding (Table S28, subgroups A–D); they do suggest that the prognostic signal of early glycemic variability belongs mainly to sepsis with organ dysfunction, since the association was weak and not statistically significant in patients coded for sepsis without organ dysfunction.

As a single-center study, the findings may apply to comparable tertiary acute care settings, but differences in glucose monitoring, treatment protocols and casemix may limit extrapolation. In settings with tighter glycemic control or continuous glucose monitoring, both the distribution of the coefficient of variation and its relationship with outcome may differ from those observed with the intermittent, clinically driven sampling available here.

### 4.5. Clinical Implications and Future Directions

Early glycemic variability was independently associated with mortality in patients without diabetes, but it improved discrimination only marginally beyond baseline clinical variables and negligibly once coded organ dysfunction was considered. Association, independent prognostic information, improvement in discrimination and clinical utility are different things, and only the first two were shown here. A role for CV-24 h in risk stratification has not been demonstrated; its absolute magnitude, a few percentage points of mortality per 0.10 increase, indicates a modest signal, and it should be regarded as hypothesis-generating until validated against established severity scores such as SOFA in prospective cohorts. Its relevance lies in interpretation: the same degree of early glycemic variability carried different prognostic meaning in patients with and without diabetes, which argues against treating acute dysglycemia as a uniform risk marker.

For anesthesia and critical care, where septic patients are managed with vasopressors, mechanical ventilation, corticosteroids, nutrition and insulin, two points follow. Stress hyperglycemia and marked glycemic variability in a patient without known diabetes are signals of severity that may more significantly warrant clinical reassessment than would the same values in a patient with chronic diabetes, in whom they partly reflect habitual glycemia and treatment. Serial glucose measurement remains justified in all septic patients because hypoglycemia and hyperglycemia were each associated with mortality, regardless of diabetes status. This study does not establish glucose targets, does not show that reducing glycemic variability improves survival, and does not support changing insulin or nutrition practice. Future studies should test whether early variability adds discrimination beyond that provided by severity scores, characterize habitual glycemia at admission to identify undiagnosed diabetes, record insulin treatment and diabetes duration so that effect modification can be examined by habitual glycemic state rather than by diagnostic label, and validate the findings in other healthcare systems through standardized or continuous glucose monitoring.

### 4.6. Strengths and Limitations

The strengths of this study include the large cohort of consecutive admissions over 14 years; the three exposure windows; the formal, hypothesis-driven testing of effect modification with concordant interaction and stratified analyses; the explicit handling of measurement intensity and of in-hospital interventions with uncertain timing; and the sensitivity analyses addressing informative observation, case ascertainment and coded severity.

Several limitations apply. First, no structured severity score (SOFA, APACHE II), lactate, vasopressor requirement, mean arterial pressure, baseline renal function or oxygenation parameters were available, and the retrospective design precludes causal inference. Residual confounding by illness severity is the principal alternative explanation for the associations reported because severe physiological disturbance can produce both glycemic variability and death. Adjustment for coded acute organ dysfunction, a coarse discharge-based variable without timing or gradation, attenuated the association between variability and death in patients without diabetes while not abolishing it (Table S29); whether early variability is a causal mediator or a marker of unmeasured severity cannot be determined, and the conclusions are confined to observational associations. Second, no treatment data were available (e.g., those regarding glucose-lowering drugs, corticosteroids, dextrose-containing infusions, nutrition, glucose-control practice, microbiological results, antimicrobial therapy, and timing of source control); thus, early variability may partly reflect clinical management rather than disease biology, and differences between patients with and without diabetes may partly reflect differences in management; organism-specific coding was present for fewer than 10% of sepsis codes, precluding pathogen-specific analyses. Third, glucose sampling was driven by clinical practice: CV-24 h could be computed in 6450 patients (59.5%) from a median of two measurements (IQR 2-3). With two values, the coefficient of variation is a normalized difference between two observations rather than a measure of temporal instability, and part of its prognostic information in patients without diabetes was carried by values in the hypoglycemic and hyperglycemic ranges (Table S39); the estimates were, however, similar with three or more measurements and not sensitive to the interval between measurements (Tables S26 and S39). The first glucose measurement is not a biological time zero; it preceded the admission timestamp in 86.6% of patients, and the interaction was reproduced with admission-anchored 24 h windows but not with the admission-anchored 48 h window (Table S27). Fourth, CV can be computed only in patients who survive to a second measurement: one quarter of all deaths occurred within 24 h of admission, and two thirds of these patients never had a second measurement. The CV analyses are therefore conditional on survival to re-measurement and are not directly comparable with full-cohort estimates (Tables S9 and S24). The effect modification was robust to weighting and to exclusion of deaths within 24 h but attenuated when deaths within 48-72 h were excluded, and it was less precisely estimated in some subgroups and with certain specifications (three or more measurements, non-ventilated patients, 24 h landmark cohort with adjustment for in-hospital interventions), for which its direction was consistent but confidence intervals included 1; therefore, residual informative observation cannot be excluded. Fifth, sepsis was ascertained from discharge documentation, which does not correspond to prospectively applied Sepsis-3 criteria and does not allow sepsis onset to be timed relative to the glucose measurements; 37.6% of the cohort had no coded organ dysfunction, although the findings were consistent across ascertainment definitions (Table S28). Sixth, pre-existing diabetes was ascertained from ICD-10-AM codes without chart-review validation, and glycated hemoglobin was not available. The diabetes group was almost entirely type 2 (96.9%), but insulin treatment, disease duration and glycemic control could not be identified, and the group without diabetes includes patients with undiagnosed diabetes; in the Romanian adult population, about one in five cases of diabetes is undiagnosed [32]. The effect modification reported here is therefore defined by documented diabetes status rather than by habitual glycemic state; non-differential misclassification of undiagnosed diabetes would be expected to attenuate rather than create the contrast. Diabetes was coded less often among patients who died within 24 h of admission, but the lower adjusted odds of death in patients with diabetes persisted after their exclusion (Section 4.3). Seventh, the diabetes stratum was smaller, limiting the power to detect a modest association, and, apart from the family of interaction tests, no adjustment for multiple comparisons was applied, so secondary findings should be regarded as supportive and exploratory findings as hypothesis-generating. Finally, this was a single-center study based on data collected for clinical rather than research purposes, so unmeasured confounding and variation in documentation practice over 14 years cannot be excluded, although calendar year was included in all adjusted models, and the findings were similar across calendar periods (Table S38); post-discharge vital status was not available, so mortality at fixed time points could not be estimated.

## 5. Conclusions

Early glycemic variability, estimated from routine intermittent measurements, was associated with in-hospital mortality mainly in septic patients without pre-existing diabetes, with a weaker association in those with diabetes. This effect modification relates predominantly to early mortality: in patients without diabetes the association was concentrated in deaths within the first days of hospitalization, and it was not consistently maintained in landmark analyses restricted to patients surviving the first 48–72 h. Diabetes also modified the prognostic meaning of early hyperglycemia and mean glucose levels, but not, detectably, of hypoglycemia. Pre-existing diabetes was associated with modestly lower observed mortality, about four percentage points lower, including among patients surviving the first day; this should not be interpreted as a protective effect. Early dysglycemia should be interpreted in context rather than as a uniform risk marker: marked early variability in a patient without diabetes was a stronger short-term prognostic signal than was comparable variability in a patient with chronic diabetes. These are observational prognostic associations. Whether they reflect a direct effect of glycemic variability or the severity of the underlying illness, its treatment, and its monitoring cannot be resolved with these data; early variability improved prediction only marginally beyond clinical variables, and no glucose target or monitoring strategy follows from these results. Whether glycemic management should be individualized according to chronic metabolic background requires prospective evaluation in cohorts with complete severity, microbiological and treatment data.

## Figures and Tables

**Table 1:** Characteristics of the sepsis cohort according to pre-existing diabetes status.

Characteristic	Total (n = 10,832)	No Diabetes (n = 7777)	Diabetes (n = 3055)	*p*
Age, years	72 (61–81)	72 (59–82)	71 (64–79)	0.913
Male sex	5464 (50.4%)	3933 (50.6%)	1531 (50.1%)	0.668
*Documented infection focus*				
Pneumonia	2176 (20.1%)	1606 (20.7%)	570 (18.7%)	0.020
Peritonitis	742 (6.9%)	629 (8.1%)	113 (3.7%)	<0.001
*Clostridioides difficile* infection	620 (5.7%)	442 (5.7%)	178 (5.8%)	0.773
*Comorbidities*				
Malignancy	1866 (17.2%)	1442 (18.5%)	424 (13.9%)	<0.001
Chronic kidney disease	1638 (15.1%)	986 (12.7%)	652 (21.3%)	<0.001
Cirrhosis	434 (4.0%)	303 (3.9%)	131 (4.3%)	0.349
COPD	626 (5.8%)	426 (5.5%)	200 (6.5%)	0.032
*Presentation*				
First glucose, mmol/L	6.9 (5.6-9.6)	6.4 (5.3–8.2)	9.9 (6.9–14.8)	<0.001
*Coded acute organ dysfunction*				
Organ systems affected: 0	4077 (37.6%)	2906 (37.4%)	1171 (38.3%)	0.829
Organ systems affected: 1	3604 (33.3%)	2597 (33.4%)	1007 (33.0%)	
Organ systems affected: 2	2005 (18.5%)	1446 (18.6%)	559 (18.3%)	
Organ systems affected: ≥3	1146 (10.6%)	828 (10.6%)	318 (10.4%)	
Shock (coded or septic shock in discharge diagnosis)	1585 (14.6%)	1180 (15.2%)	405 (13.3%)	0.011
Acute respiratory failure	4008 (37.0%)	2897 (37.3%)	1111 (36.4%)	0.391
Acute kidney injury	2773 (25.6%)	1949 (25.1%)	824 (27.0%)	0.040
*Hospital course (post-baseline)*				
Surgical intervention	2675 (24.7%)	1967 (25.3%)	708 (23.2%)	0.021
Mechanical ventilation	2319 (21.4%)	1595 (20.5%)	724 (23.7%)	<0.001
Renal replacement therapy	682 (6.3%)	475 (6.1%)	207 (6.8%)	0.198
Length of stay, days	8 (3–15)	8 (3–15)	9 (4–16)	<0.001
In-hospital mortality	5803 (53.6%)	4245 (54.6%)	1558 (51.0%)	<0.001

Data are median (interquartile range) or *n* (%). *p* values refer to comparisons between patients with and without diabetes; continuous variables were compared using the Mann–Whitney U test and categorical variables via the Chi-square test. Documented infection foci are those recorded in the discharge record and are not mutually exclusive. First glucose is the first glucose value associated with the index presentation. Coded acute organ dysfunction was derived from discharge diagnoses (codes in Section 2.4) and may include dysfunction arising after admission; shock includes septic shock documented in the discharge diagnosis. Glycated hemoglobin was not routinely measured and is not available for the cohort; diabetes treatment and initial lactate were not recorded; ICU admission was not reliably recorded in the administrative dataset. Length of stay is a post-baseline variable truncated by death. COPD, chronic obstructive pulmonary disease; ICU, intensive care unit.

**Table 2:** Patient characteristics, in-hospital interventions, and early glycemic metrics according to in-hospital survival status.

Characteristic	Total (n = 10,832)	Survivors (n = 5029)	Non-Survivors (n = 5803)	*p*
Age, years	72 (61–81)	68 (54–78)	75 (66–83)	<0.001
Male sex	5464 (50.4%)	2706 (53.8%)	2758 (47.5%)	<0.001
Diabetes	3055 (28.2%)	1497 (29.8%)	1558 (26.8%)	<0.001
*Documented infection focus*				
Pneumonia	2176 (20.1%)	642 (12.8%)	1534 (26.4%)	<0.001
Peritonitis	742 (6.9%)	452 (9.0%)	290 (5.0%)	<0.001
*Clostridioides difficile* infection	620 (5.7%)	288 (5.7%)	332 (5.7%)	0.990
*Comorbidities*				
Malignancy	1866 (17.2%)	739 (14.7%)	1127 (19.4%)	<0.001
Chronic kidney disease	1638 (15.1%)	626 (12.4%)	1012 (17.4%)	<0.001
Cirrhosis	434 (4.0%)	182 (3.6%)	252 (4.3%)	0.055
COPD	626 (5.8%)	253 (5.0%)	373 (6.4%)	0.002
*Early glycemic metrics, first 24 h*				
First glucose, mmol/L	6.9 (5.6–9.6)	6.7 (5.5–9.1)	7.2 (5.6–9.9)	<0.001
Number of measurements	2 (1–2)	2 (1–2)	2 (1–2)	0.306
Mean glucose, mmol/L	7.0 (5.6–9.5)	6.7 (5.5–9.0)	7.3 (5.7–10.0)	<0.001
TWM-24 h, mmol/L (available in 10,802)	7.1 (5.6–9.6)	6.8 (5.5–9.1)	7.4 (5.8–10.1)	<0.001
CV-24 h (available in 6450, 59.5%)	0.203 (0.100–0.357)	0.190 (0.098–0.329)	0.216 (0.102–0.388)	<0.001
Minimum glucose, mmol/L	6.2 (4.9–8.3)	6.1 (4.9–7.8)	6.4 (4.9–8.7)	<0.001
Maximum glucose, mmol/L	7.7 (6.0–10.9)	7.3 (5.8–10.3)	8.1 (6.2–11.6)	<0.001
Hypoglycemia < 3.9 mmol/L	1083 (10.0%)	361 (7.2%)	722 (12.4%)	<0.001
Hypoglycemia < 3.0 mmol/L	505 (4.7%)	123 (2.4%)	382 (6.6%)	<0.001
Hyperglycemia > 10.0 mmol/L	3268 (30.2%)	1318 (26.2%)	1950 (33.6%)	<0.001
*Hospital course (post-baseline)*				
Surgical intervention	2675 (24.7%)	1719 (34.2%)	956 (16.5%)	<0.001
Mechanical ventilation	2319 (21.4%)	461 (9.2%)	1858 (32.0%)	<0.001
Renal replacement therapy	682 (6.3%)	316 (6.3%)	366 (6.3%)	0.960
Length of stay, days	8 (3–15)	11 (6–17)	4 (1–12)	<0.001

Data are median (interquartile range) or *n* (%). *p* values refer to comparisons between survivors and non-survivors; continuous variables were compared using the Mann–Whitney U test and categorical variables via the Chi-square test. First glucose is the first glucose value associated with the index presentation. Hypoglycemia and hyperglycemia indicate at least one value below or above the specified threshold during the first 24 h. Length of stay is shortened by early death and is not comparable between survivors and non-survivors; it is shown for descriptive purposes and was not used in any model. To convert glucose to mg/dL, multiply by 18.0. COPD, chronic obstructive pulmonary disease; CV, coefficient of variation; TWM, time-weighted mean glucose.

**Table 3:** Early glycemic profile within 24 h of the first glucose measurement according to pre-existing diabetes status.

Variable	Diabetes (*n* = 3055)	No Diabetes (*n* = 7777)	*p*
First glucose, mmol/L	9.9 (6.9–14.8)	6.4 (5.3–8.2)	<0.001
TWM-24 h, mmol/L (available in 10,802)	9.8 (7.1–14.2)	6.5 (5.4–8.2)	<0.001
CV-24 h (available in 6450)	0.229 (0.113–0.396)	0.193 (0.096–0.338)	<0.001
Number of measurements, 24 h	2 (1–2)	2 (1–2)	<0.001
Mean glucose, mmol/L	9.8 (7.1–14.1)	6.5 (5.4–8.2)	<0.001
Minimum glucose, mmol/L	8.3 (5.9–12.1)	5.8 (4.8–7.2)	<0.001
Maximum glucose, mmol/L	11.0 (7.8–16.3)	7.1 (5.7–9.2)	<0.001
Hypoglycemia < 3.9 mmol/L (70 mg/dL)	264 (8.6%)	819 (10.5%)	0.003
Hypoglycemia < 3.0 mmol/L (54 mg/dL)	135 (4.4%)	370 (4.8%)	0.452
Hyperglycemia > 10.0 mmol/L (180 mg/dL)	1746 (57.2%)	1522 (19.6%)	<0.001

Data are median (interquartile range) or *n* (%). *p* values refer to comparisons between patients with and without diabetes; continuous variables were compared using the Mann–Whitney U test and categorical variables via the Chi-square test. To convert glucose to mg/dL, multiply by 18.0. CV, coefficient of variation; TWM, time-weighted mean glucose.

**Table 4:** Association between pre-existing diabetes and in-hospital mortality across sequential logistic regression models.

Model	Covariate Adjustment	N	OR for Diabetes (95% CI)	*p*
Model 1	Unadjusted	10,832	0.866 (0.796–0.942)	<0.001
Model 2	Year of admission, age, sex, malignancy, pneumonia, cirrhosis, CKD, COPD, peritonitis	10,832	0.822 (0.751–0.899)	<0.001
Model 3	Model 2 + hypoglycemia < 3.9 mmol/L, TWM-24 h, CV-24 h, number of glucose measurements	6434	0.691 (0.610–0.784)	<0.001
Model 4 (sensitivity)	Model 2 + mechanical ventilation, renal replacement therapy, surgical intervention	10,832	0.776 (0.705–0.854)	<0.001
Model 5 (sensitivity)	Model 4 + hypoglycemia < 3.9 mmol/L, TWM-24 h, CV-24 h, number of glucose measurements	6434	0.677 (0.591–0.776)	<0.001

Odds ratios with 95% confidence intervals were estimated with binary logistic regression, with in-hospital mortality as the dependent variable. *N* indicates the number of patients in each model; differences reflect complete-case analysis and missingness in glycemic variables, primarily CV-24 h (Table S36). Model 3 conditions on early glycemic variables that may lie on the pathway between diabetes and death; Models 4 and 5 condition on post-baseline variables and are sensitivity analyses subject to over-adjustment (Figure 2). CI, confidence interval; CKD, chronic kidney disease; COPD, chronic obstructive pulmonary disease; CV, coefficient of variation; OR, odds ratio; TWM, time-weighted mean glucose.

**Table 5:** Effect modification by pre-existing diabetes. Panel A: interaction between diabetes and each early glycemic parameter (24 h, primary models). Panel B: diabetes-stratified association between early glycemic variability and in-hospital mortality. Panel C: standardized predicted in-hospital mortality according to CV-24 h.

**Panel A.** Interaction models						
**Model**	**Variable**	**N**	**OR (95% CI)**	***p***	***p*** **for Interaction**	***p*** **for Interaction, Holm**
A	TWM-24 h (per 1 mmol/L)	10,802	1.062 (1.044–1.081)	<0.001		
	Diabetes		0.973 (0.793–1.194)	0.793		
	TWM-24 h × diabetes		0.963 (0.943–0.984)		<0.001	0.005
B	CV-24 h (per 0.10)	6450	1.136 (1.101–1.171)	<0.001		
	Diabetes		1.006 (0.845–1.198)	0.944		
	CV-24 h × diabetes		0.912 (0.868–0.959)		<0.001	0.003
C	Hypoglycemia < 3.9 mmol/L	10,832	2.218 (1.878–2.619)	<0.001		
	Diabetes		0.850 (0.774–0.934)	<0.001		
	Hypoglycemia × diabetes		0.766 (0.557–1.052)		0.099	0.298
D	Hyperglycemia > 10.0 mmol/L	10,832	1.755 (1.544–1.995)	<0.001		
	Diabetes		0.801 (0.706–0.908)	<0.001		
	Hyperglycemia × diabetes		0.722 (0.594–0.878)		0.001	0.009
**Panel B.** Diabetes-stratified models						
**Cohort**		**Model**	***N***	**Adjusted OR (95% CI) per 0.10 Increase in CV-24 h**		***p***
No diabetes		Primary	4455	1.137 (1.102–1.173)		<0.001
No diabetes		Sensitivity	4455	1.116 (1.080–1.154)		<0.001
Diabetes		Primary	1995	1.034 (0.994–1.076)		0.099
Diabetes		Sensitivity	1995	1.040 (0.996–1.086)		0.077
**Panel C.** Standardized predicted in-hospital mortality						
**CV-24 h**		**No Diabetes, % (95% CI)**		**Diabetes, % (95% CI)**		
0.10		51.3 (49.6–53.0)		49.2 (46.3–52.0)		
0.20		54.2 (52.7–55.7)		50.0 (47.6–52.3)		
0.30		57.0 (55.5–58.4)		50.8 (48.7–52.8)		
0.40		59.8 (58.1–61.5)		51.7 (49.3–53.9)		
0.50		62.5 (60.3–64.8)		52.5 (49.5–55.3)		
Marginal risk difference per 0.10 increase		2.79 pp (2.09 to 3.49)		0.82 pp (−0.13 to 1.77)		
Difference between strata, percentage points		1.97 pp (0.83 to 3.08)				

Primary models were adjusted for calendar year, sex, age, malignancy, pneumonia, cirrhosis, chronic kidney disease, chronic obstructive pulmonary disease, peritonitis, and number of glucose measurements within the first 24 h; sensitivity models were additionally adjusted for mechanical ventilation, renal replacement therapy, and surgical intervention. Odds ratios for TWM-24 h are expressed per 1 mmol/L increment (equivalent to 18 mg/dL), computed from the unrounded fitted per-mg/dL coefficient. Odds ratios for CV-24 h refer to a 0.10 increase (10 percentage points); an OR > 1 indicates higher odds of in-hospital death with increasing early glycemic variability. In Panel A, the OR for each glycemic parameter refers to patients without diabetes; multiplying it by the interaction OR approximates the corresponding OR in patients with diabetes (e.g., CV-24 h: 1.136 × 0.912 ≈ 1.04, consistent with the stratified estimate in Panel B); Holm adjustment was applied across twelve interaction tests (four parameters, three windows; Table S33). Panel C: standardized estimates by g-computation from the primary interaction model, with 95% CIs from 500 bootstrap resamples. In Panels B and C, *N* is lower than the full cohort because the coefficient of variation requires at least two glucose measurements within the window (n = 6450). Hospital length of stay was not included because it is a post-baseline variable. CI, confidence interval; CV, coefficient of variation; OR, odds ratio; pp, percentage points; TWM, time-weighted mean glucose.

**Table 6:** Robustness of the diabetes-by-CV interaction to informative observation: inverse-probability-of-observation weighting, restriction to denser sampling and landmark restriction.

Analysis	N (No Diabetes/Diabetes)	Mortality	Interaction OR per 0.10 (95% CI)	*p*	CV OR per 0.10, No Diabetes (95% CI)	CV OR per 0.10, Diabetes (95% CI)
Primary 24 h model (Table 5)	6450 (4455/1995)	54.0%	0.912 (0.868–0.959)	<0.001	1.137 (1.102–1.173)	1.034 (0.994–1.076)
IPW, propensity from baseline covariates and first glucose	6450 (4455/1995)	54.0%	0.913 (0.868–0.960)	<0.001	1.140 (1.106–1.176)	1.037 (0.997–1.080)
IPW, propensity additionally including MV, RRT, surgery	6450 (4455/1995)	54.0%	0.918 (0.873–0.965)	<0.001	1.137 (1.103–1.173)	1.039 (0.998–1.082)
≥3 glucose measurements within 24 h	1837 (1251/586)	54.4%	0.911 (0.825–1.006)	0.065	1.154 (1.086–1.226)	1.044 (0.964–1.131)
Landmark: alive at 24 h after first measurement, CV-24 h	6123 (4197/1926)	51.8%	0.938 (0.891–0.988)	0.016	1.092 (1.056–1.128)	1.023 (0.982–1.066)
Landmark 24 h, additionally adjusted for MV, RRT, surgery	6123 (4197/1926)	51.8%	0.963 (0.911–1.018)	0.180	1.075 (1.038–1.113)	1.031 (0.986–1.078)
Landmark 24 h combined with IPW (propensity including MV, RRT, surgery, re-estimated in the landmark cohort)	6123 (4197/1926)	51.8%	0.940 (0.893–0.991)	0.021	1.096 (1.061–1.133)	1.028 (0.986–1.071)
Landmark: alive at 48 h, CV-24 h	5539 (3769/1770)	47.6%	0.974 (0.922–1.030)	0.352	1.049 (1.012–1.088)	1.022 (0.979–1.067)
Landmark: alive at 72 h, CV-24 h	5183 (3529/1654)	45.2%	0.975 (0.920–1.034)	0.398	1.042 (1.004–1.083)	1.017 (0.972–1.064)
Landmark: alive at 48 h, CV-48 h	7010 (4812/2198)	47.0%	0.987 (0.936–1.040)	0.629	1.046 (1.012–1.081)	1.028 (0.985–1.073)
Landmark: alive at 72 h, CV-72 h	7079 (4874/2205)	44.3%	0.974 (0.920–1.030)	0.354	1.055 (1.021–1.090)	1.024 (0.976–1.073)
Exclusion of patients with first glucose >24 h before admission	6402 (4419/1983)	53.9%	0.911 (0.867–0.958)	<0.001	1.138 (1.103–1.174)	1.034 (0.994–1.076)

All models were adjusted for calendar year, age, sex, malignancy, pneumonia, cirrhosis, chronic kidney disease, chronic obstructive pulmonary disease, peritonitis, and number of glucose measurements within the corresponding window. Landmark cohorts comprise patients alive and hospitalized at the stated time after the first glucose measurement. IPW, inverse-probability-of-observation weighting with stabilized weights (range of 0.63–1.77 and 0.61–2.24 for the two propensity models) and robust standard errors; the propensity model estimated the probability of CV-24 h being available from diabetes status, first glucose value and the baseline covariates. CV odds ratios refer to a 0.10 increase; the interaction OR is the ratio of the CV odds ratio in patients with diabetes to that in patients without diabetes. Further detail on the landmark cohorts and on the minimum number of measurements is given in Tables S25 and S26. MV, mechanical ventilation; RRT, renal replacement therapy. Of the 6450 patients with CV-24 h (3481 deaths), 327 (310 deaths) left the hospital before 24 h, 911 (844 deaths) before 48 h and 1267 (1136 deaths) before 72 h after the first glucose measurement.

## Data Availability

The datasets analyzed during the current study are not publicly available because they are derived from the electronic health records of the University Emergency Hospital of Bucharest and contain potentially identifying clinical information. De-identified data are available from the corresponding author on reasonable request, subject to approval by the relevant institutional and ethics committees. The analysis code is available from the corresponding author on reasonable request.

## Supplementary Materials

The following supporting information can be downloaded at: https://www.mdpi.com/article/10.3390/jcm15218649/s1, Supplementary Methods S1: Derivation of time-weighted mean glucose; Supplementary Methods S2: Specification of the statistical analyses; Table S1: Distribution of sepsis-related ICD-10-AM codes; Table S2: ICD-10-AM codes for diabetes mellitus identified in the cohort; Tables S3 and S4: Early glycemic profile according to diabetes status at 48 h and 72 h; Table S5: Full output of Models 2 and 3; Tables S6 and S7: Association between pre-existing diabetes and in-hospital mortality at 48 h and 72 h; Table S8: Independent association of early glycemic parameters with in-hospital mortality at 24 h; Table S9: Comparison of patients with and without available CV-24 h; Tables S10–S14: Independent association of early glycemic parameters with in-hospital mortality, sensitivity analyses at 24 h and primary and sensitivity analyses at 48 h and 72 h; Tables S15–S19: Interaction analyses at 24 h (sensitivity), 48 h, and 72 h; Tables S20 and S21: Diabetes-stratified association between early glycemic variability and in-hospital mortality at 48 h and 72 h; Table S22: Comparison of the 2022 and 2026 meta-analyses on diabetes and mortality in sepsis; Table S23: In-hospital mortality according to the number of glucose measurements within 24 h; Table S24: Timing of the second glucose measurement and availability of CV-24 h according to diabetes status and timing of death; Table S25: Diabetes-stratified association between early glycemic variability and in-hospital mortality in landmark cohorts; Table S26: Sensitivity of the principal CV estimates to the minimum number of glucose measurements; Table S27: Timing of the first glucose measurement relative to admission and admission-anchored exposure windows; Table S28: Sensitivity of the principal findings to sepsis ascertainment and mortality by identification route; Table S29: Coded acute organ dysfunction and adjustment for it; Table S30: Diabetes and in-hospital mortality in landmark cohorts and comorbidity coding according to timing of death; Table S31: Absolute measures of the association between early glycemic variability and in-hospital mortality and observed mortality by quartile of CV-24 h; Table S32: Exploratory restricted cubic spline analysis; Table S33: Adjustment for multiple testing across the interaction tests; Table S34: Diabetes and CV estimates under adjustment sets derived from the causal model; Table S35: Incremental predictive performance of CV-24 h; Table S36: Reconciliation of analytic denominators and number of deaths per model; Table S37: Principal CV estimates stratified by mechanical ventilation; Table S38: Cohort characteristics and principal CV estimates by calendar period; Table S39: Sensitivity of the principal CV estimates to extreme values and sampling intervals; Table S40: Standardized in-hospital mortality, risk difference and risk ratio for pre-existing diabetes in the full cohort and in landmark cohorts. Odds ratios for CV are reported per 0.10 increase throughout.

## Abbreviations

The following abbreviations are used in this manuscript: APACHE, Acute Physiology and Chronic Health Evaluation; CI, confidence interval; CKD, chronic kidney disease; COPD, chronic obstructive pulmonary disease; CV, coefficient of variation; EHR, electronic health record; ICD-10-AM, International Statistical Classification of Diseases and Related Health Problems, Tenth Revision, Australian Modification; ICU, intensive care unit; IPW, inverse-probability-of-observation weighting; IQR, interquartile range; MV, mechanical ventilation; OR, odds ratio; RRT, renal replacement therapy; SOFA, Sequential Organ Failure Assessment; STROBE, Strengthening the Reporting of Observational Studies in Epidemiology; TWM, time-weighted mean glucose.
