# Supplementary material for: Early Glycemic Variability from Routine Glucose Measurements, Diabetes Status, and In-Hospital Mortality in Sepsis: A 14-Year Retrospective Cohort Study
Source: J Clin Med. 2026 Nov 6;15(21):8649. doi: 10.3390/jcm15218649 (PMC13764494; doi:10.3390/jcm15218649)
Supplement: Supplementary file 1 [file jcm-15-08649-s001.zip › JCM_Supplementary_Materials_R2.pdf]

Supplementary Materials

Early Glucose Variability from Routine Measurements, Diabetes Status, and In-Hospital Mortality in Sepsis: A 14-Year Retrospective Cohort Study

Unless otherwise stated, descriptive glucose values are reported in mmol/L (to convert to mg/dL, multiply by 18.0). Odds ratios for the coefficient of variation (CV), expressed as a proportion, are reported per 0.10 increase in CV throughout, including interaction terms. In regression tables, odds ratios for time-weighted mean glucose (TWM) are reported per 1 mg/dL, as fitted; the corresponding main-text estimates were rescaled to per 1 mmol/L. Pre-existing diabetes was defined as any ICD-10-AM code E10-E14 among the discharge diagnoses (Table S2). Diagnoses were coded using the International Statistical Classification of Diseases and Related Health Problems, Tenth Revision, Australian Modification (ICD-10-AM). Glycemic thresholds: hypoglycemia < 3.9 mmol/L (70 mg/dL); severe hypoglycemia < 3.0 mmol/L (54 mg/dL); hyperglycemia > 10.0 mmol/L (180 mg/dL). CKD, chronic kidney disease; COPD, chronic obstructive pulmonary disease; CI, confidence interval; CV, coefficient of variation; OR, odds ratio.

Supplementary Methods

S1. Derivation of Time-Weighted Mean Glucose

For each patient, glucose measurements were ordered chronologically, and the first available value (time t_1) defined the start of the observation window $[t_1, t_1 + W]$, where W = 24, 48, or 72 h. For the k measurements $g_1, \ldots, g_k$ recorded within the window at times $t_1 \leq t_2 \leq \ldots \leq t_k$, each value was weighted by the interval until the next measurement from the same patient, $\Delta t_i = t_{i+1} - t_i$ for $i < k$. The last measurement within the window was carried forward to the end of the window, $\Delta t_k = (t_1 + W) - t_k$, and intervals extending beyond the window were truncated at the window boundary, so that measurements recorded after the end of the window received a weight of zero. TWM was calculated as:

$$\mathrm{TWM} = \Sigma\ (g_i \times \Delta t_i) / \Sigma\ \Delta t_i, \quad i = 1, \ldots, k$$

that is, the sum of glucose values multiplied by their corresponding intervals, divided by the total weighted time observed. For patients with a single measurement within the window, TWM equals that measurement.

S2. Specification of the Statistical Analyses

Effect modification (main text, Section 2.9.3). Effect modification by pre-existing diabetes was assessed by entering interaction terms between diabetes status and each glycemic parameter into adjusted logistic regression models, with separate interaction models fitted for TWM, CV, hypoglycemia < 3.9 mmol/L, and hyperglycemia > 10.0 mmol/L, each adjusted for the baseline covariates (age, sex, calendar year, malignancy, pneumonia, cirrhosis, CKD, COPD and peritonitis; main text, Section 2.4) and the number of glucose measurements. Interaction was assessed on the multiplicative scale: the OR for the glycemic parameter in each interaction model refers to patients without diabetes, and the interaction OR is the ratio of the corresponding OR in patients with diabetes to that in patients without diabetes (for CV, both per 0.10 increase), so that their product approximates the OR in patients with diabetes. Stratified models, fitted separately in patients with and without pre-existing diabetes, used the same covariates; sensitivity versions additionally included mechanical ventilation, renal replacement therapy and surgical intervention.

Absolute-scale estimates (main text, Section 2.9.3). Because in-hospital mortality exceeded 50%, odds ratios substantially overstate relative risks. To express the diabetes-stratified associations on an absolute scale, standardized predicted mortality was estimated by g-computation from the primary interaction model, setting CV-24h to 0.10, 0.20, 0.30, 0.40 and 0.50 for all patients in each stratum and averaging the predicted probabilities; marginal risk differences and risk ratios per 0.10 increase in CV were obtained in the same way, with 95% confidence intervals from 500 bootstrap resamples. Effect modification on the additive scale was assessed as the difference in marginal risk differences between strata. Risk ratios were additionally estimated by modified Poisson regression with robust standard errors.

Non-linearity (main text, Section 2.9.4). Non-linearity of the association between CV-24h and in-hospital mortality was explored with restricted cubic splines fitted separately in patients with and without diabetes and adjusted for the covariates of the primary interaction model. The primary specification used four knots placed at the 5th, 35th, 65th and 95th percentiles of the stratum-specific CV distribution; three and five knots were used as sensitivity analyses. Non-linearity was tested by the likelihood-ratio test of the spline against the linear model, and differences in shape between strata by the likelihood-ratio test of spline-by-diabetes product terms. Odds ratios along the curve were expressed relative to the stratum-specific median. Because, with two measurements, CV is a deterministic function of their difference, the spline and linear models were repeated separately in patients with exactly two and with three or more measurements within 24 h.

Informative observation and survivor bias (main text, Section 2.9.5). To characterize and address informative observation, we (i) tabulated in-hospital mortality according to the number of glucose measurements within 24 h; (ii) described the timing of the second glucose measurement and the cumulative proportion of patients with a second measurement before 6, 12, 18 and 24 h; (iii) compared the availability of CV-24h between patients who died within 24 h of admission, those who died later, and survivors; (iv) repeated the interaction and diabetes-stratified CV models in landmark cohorts restricted to patients alive and hospitalized at 24, 48 and 72 h after the first glucose measurement, including a strict version in which CV-24h was related to death only among patients alive at 48 h and at 72 h, so that the exposure was completely observed before the landmark; (v) repeated the 24 h models in patients with at least three measurements; and (vi) applied inverse-probability-of-observation weighting, in which the probability of CV-24h being available was estimated by logistic regression from diabetes status, first glucose value and the baseline covariates (and, in a second model, also mechanical ventilation, renal replacement therapy and surgical intervention), and the CV models were refitted with stabilized weights and robust standard errors. The landmark restriction was also applied to the association between diabetes and mortality. Because weighting on measured characteristics cannot correct selection through survival itself, the landmark analyses were regarded as the principal robustness test of the effect-modification finding.

Sensitivity analyses (main text, Section 2.9.7). The sensitivity analyses of the principal CV models were: (i) exclusion of the 121 patients whose first glucose measurement preceded admission by more than 24 h (main text, Section 2.5); (ii) restriction to patients with at least 3 and at least 4 measurements within 24 h and with at least 3, 4 and 5 measurements within 48 h and 72 h, with a CV-by-measurement-count interaction term added to the diabetes-stratified 24 h models; (iii) recomputation of the glycemic metrics from the individual glucose records in three windows anchored to the administrative admission timestamp (admission to 24 h after; 24 h before to 24 h after, retaining emergency-department values; admission to 48 h after); (iv) restriction to patients with any explicit sepsis code,

with a sepsis code as principal diagnosis, with a sepsis code and at least one coded acute organ dysfunction (main text, Section 2.4), or with septic shock recorded in the discharge diagnosis text, with patients coded for sepsis without organ dysfunction analyzed as a contrast group; (v) additional adjustment for the number of organ systems with coded acute dysfunction (0, 1, 2, ≥3), alone and together with in-hospital interventions; (vi) stratification by mechanical ventilation as a proxy for intensive care unit (ICU) treatment, because ICU admission was not reliably recorded in the administrative dataset, with heterogeneity assessed by a three-way interaction term; (vii) stratification by calendar period (2011-2015, 2016-2019 and 2020-2024), with heterogeneity tested by three-way interaction terms; (viii) additional adjustment for the time span of the measurements within 24 h or for the interval between the first two measurements, restriction to patients whose second measurement was obtained at least 6 h after the first, additional adjustment for hypoglycemia and hyperglycemia, and exclusion of patients with any value below 54 mg/dL or above 400 mg/dL, with any value below 70 mg/dL, or with any value above 180 mg/dL; and (ix) quantification of the glucose results reported outside the measuring range (main text, Section 2.5).

Incremental predictive performance (main text, Section 2.9.8). To assess whether CV-24h improved prediction of in-hospital mortality, a base logistic model including the Model 2 covariates and the number of glucose measurements was compared with the same model plus CV-24h, separately in patients with and without diabetes. Performance was assessed by the likelihood-ratio test, the C-statistic (apparent and optimism-corrected by 200 bootstrap resamples), the change in C-statistic with a bootstrap 95% confidence interval, the Brier score, the optimism-corrected calibration slope, observed versus predicted risk by decile with the Hosmer-Lemeshow statistic, the continuous net reclassification improvement and the integrated discrimination improvement. The comparison was repeated with base models additionally including coded acute organ dysfunction, and organ dysfunction together with in-hospital interventions.

Sepsis and Diabetes Ascertainment

Table S1. Distribution of sepsis-related ICD-10-AM codes in the study population. The total number of codes does not represent the number of individual patients, because a single patient could have more than one sepsis-related ICD-10-AM code recorded.

ICD-10-AM Code	Description	Number of Codes
A02.1	Sepsis due to *Salmonella*	4
A26.7	Sepsis due to *Erysipelothrix*	0
A32.7	Listerial sepsis	0
A40.0	Sepsis due to group A *Streptococcus*	8
A40.1	Sepsis due to group B *Streptococcus*	9
A40.2	Sepsis due to group D *Streptococcus*	7
A40.8	Other streptococcal sepsis	44
A40.9	Streptococcal sepsis, unspecified	23
A41.0	Sepsis due to *Staphylococcus aureus*	178
A41.1	Sepsis due to other specified staphylococci	94
A41.2	Sepsis due to unspecified staphylococci	31
A41.3	Sepsis due to *Haemophilus influenzae*	5
A41.4	Sepsis due to anaerobes	72
A41.50	Sepsis due to unspecified Gram-negative bacteria	56
A41.51	Sepsis due to *Escherichia coli*	248

ICD-10-AM Code	Description	Number of Codes
A41.52	Sepsis due to *Pseudomonas*	74
A41.58	Sepsis due to other Gram-negative organisms	138
A41.8	Other specified sepsis	6,534
A41.9	Sepsis, unspecified	3,127
A42.7	Actinomycotic sepsis	4
B37.7	Candidal sepsis	27
T81.42	Procedure-related sepsis	15
O88.3	Obstetric pyemic and septic embolism	0
**Total**		**10,698**

ICD-10-AM, International Statistical Classification of Diseases and Related Health Problems, Tenth Revision, Australian Modification.

Table S2. ICD-10-AM diabetes mellitus codes recorded in the study cohort and number of patients; patients could carry more than one code.

ICD-10-AM Code	Description	Number of Patients
E10.11	Type 1 diabetes mellitus with ketoacidosis, without coma	7
E10.12	Type 1 diabetes mellitus with ketoacidosis, with coma	5
E10.14	Type 1 diabetes mellitus with lactic acidosis, with coma	2
E10.15	Type 1 diabetes mellitus with ketoacidosis and lactic acidosis, without coma	2
E10.21	Type 1 diabetes mellitus with incipient diabetic nephropathy	1
E10.22	Type 1 diabetes mellitus with established diabetic nephropathy	5
E10.23	Type 1 diabetes mellitus with end-stage renal disease	8
E10.31	Type 1 diabetes mellitus with background retinopathy	3
E10.32	Type 1 diabetes mellitus with preproliferative retinopathy	2
E10.33	Type 1 diabetes mellitus with proliferative retinopathy	6
E10.34	Type 1 diabetes mellitus with other retinopathy	3
E10.35	Type 1 diabetes mellitus with advanced ophthalmic disease	1
E10.36	Type 1 diabetes mellitus with diabetic cataract	1
E10.40	Type 1 diabetes mellitus with neuropathy, unspecified	5
E10.42	Type 1 diabetes mellitus with diabetic polyneuropathy	9
E10.43	Type 1 diabetes mellitus with diabetic autonomic neuropathy	2
E10.49	Type 1 diabetes mellitus with other specified neurological complications	1
E10.51	Type 1 diabetes mellitus with peripheral angiopathy, without gangrene	2
E10.52	Type 1 diabetes mellitus with peripheral angiopathy, with gangrene	20
E10.53	Type 1 diabetes mellitus with diabetic ischemic cardiomyopathy	1
E10.61	Type 1 diabetes mellitus with specified diabetic musculoskeletal and connective tissue complications	1
E10.62	Type 1 diabetes mellitus with specified skin and subcutaneous tissue complications	4
E10.65	Type 1 diabetes mellitus with poor control	11
E10.69	Type 1 diabetes mellitus with other specified complications	18
E10.71	Type 1 diabetes mellitus with multiple microvascular complications	10

ICD-10-AM Code	Description	Number of Patients
E10.73	Type 1 diabetes mellitus with foot ulcer due to multiple causes	4
E10.8	Type 1 diabetes mellitus with unspecified complications	20
E10.9	Type 1 diabetes mellitus without complications	13
E11.01	Type 2 diabetes mellitus with hyperosmolarity, without nonketotic hyperglycemic-hyperosmolar coma [NKHHC]	22
E11.02	Type 2 diabetes mellitus with hyperosmolarity, with coma	9
E11.11	Type 2 diabetes mellitus with ketoacidosis, without coma	23
E11.12	Type 2 diabetes mellitus with ketoacidosis, with coma	11
E11.13	Type 2 diabetes mellitus with lactic acidosis, without coma	2
E11.14	Type 2 diabetes mellitus with lactic acidosis, with coma	4
E11.15	Type 2 diabetes mellitus with ketoacidosis and lactic acidosis, without coma	6
E11.16	Type 2 diabetes mellitus with ketoacidosis and lactic acidosis, with coma	8
E11.21	Type 2 diabetes mellitus with incipient diabetic nephropathy	13
E11.22	Type 2 diabetes mellitus with established diabetic nephropathy	165
E11.23	Type 2 diabetes mellitus with end-stage renal disease	30
E11.29	Type 2 diabetes mellitus with other specified renal complications	18
E11.31	Type 2 diabetes mellitus with background retinopathy	8
E11.32	Type 2 diabetes mellitus with preproliferative retinopathy	3
E11.33	Type 2 diabetes mellitus with proliferative retinopathy	15
E11.34	Type 2 diabetes mellitus with other retinopathy	9
E11.35	Type 2 diabetes mellitus with advanced ophthalmic disease	2
E11.36	Type 2 diabetes mellitus with diabetic cataract	1
E11.39	Type 2 diabetes mellitus with other specified ophthalmic complications	2
E11.40	Type 2 diabetes mellitus with neuropathy, unspecified	21
E11.41	Type 2 diabetes mellitus with diabetic mononeuropathy	1
E11.42	Type 2 diabetes mellitus with diabetic polyneuropathy	65
E11.43	Type 2 diabetes mellitus with diabetic autonomic neuropathy	5
E11.49	Type 2 diabetes mellitus with other specified neurological complications	4
E11.51	Type 2 diabetes mellitus with peripheral angiopathy, without gangrene	10
E11.52	Type 2 diabetes mellitus with peripheral angiopathy, with gangrene	108
E11.53	Type 2 diabetes mellitus with diabetic ischemic cardiomyopathy	6
E11.61	Type 2 diabetes mellitus with specified diabetic musculoskeletal and connective tissue complications	32
E11.62	Type 2 diabetes mellitus with specified skin and subcutaneous tissue complications	17
E11.63	Type 2 diabetes mellitus with specified periodontal complications	4
E11.64	Type 2 diabetes mellitus with hypoglycemia	16
E11.65	Type 2 diabetes mellitus with poor control	198
E11.69	Type 2 diabetes mellitus with other specified complications	650

ICD-10-AM Code	Description	Number of Patients
E11.71	Type 2 diabetes mellitus with multiple microvascular complications	195
E11.72	Type 2 diabetes mellitus with features of insulin resistance	73
E11.73	Type 2 diabetes mellitus with foot ulcer due to multiple causes	21
E11.8	Type 2 diabetes mellitus with unspecified complications	1,503
E11.9	Type 2 diabetes mellitus without complications	497
E13.01	Other specified diabetes mellitus with hyperosmolarity, without nonketotic hyperglycemic-hyperosmolar coma [NKHHC]	6
E13.11	Other specified diabetes mellitus with ketoacidosis, without coma	1
E13.12	Other specified diabetes mellitus with ketoacidosis, with coma	1
E13.13	Other specified diabetes mellitus with lactic acidosis, without coma	1
E13.15	Other specified diabetes mellitus with ketoacidosis and lactic acidosis, without coma	1
E13.21	Other specified diabetes mellitus with incipient diabetic nephropathy	2
E13.22	Other specified diabetes mellitus with established diabetic nephropathy	5
E13.23	Other specified diabetes mellitus with end-stage renal disease	4
E13.34	Other specified diabetes mellitus with other retinopathy	1
E13.40	Other specified diabetes mellitus with neuropathy, unspecified	1
E13.42	Other specified diabetes mellitus with diabetic polyneuropathy	3
E13.52	Other specified diabetes mellitus with peripheral angiopathy, with gangrene	8
E13.61	Other specified diabetes mellitus with specified diabetic musculoskeletal and connective tissue complications	2
E13.64	Other specified diabetes mellitus with hypoglycemia	1
E13.65	Other specified diabetes mellitus with poor control	1
E13.69	Other specified diabetes mellitus with other specified complications	2
E13.71	Other specified diabetes mellitus with multiple microvascular complications	1
E13.72	Other specified diabetes mellitus with features of insulin resistance	1
E13.73	Other specified diabetes mellitus with foot ulcer due to multiple causes	3
E13.9	Other specified diabetes mellitus without complications	1
E14.01	Unspecified diabetes mellitus with hyperosmolarity, without nonketotic hyperglycemic-hyperosmolar coma [NKHHC]	1
E14.02	Unspecified diabetes mellitus with hyperosmolarity, with coma	1
E14.12	Unspecified diabetes mellitus with ketoacidosis, with coma	1
E14.13	Unspecified diabetes mellitus with lactic acidosis, without coma	1
E14.14	Unspecified diabetes mellitus with lactic acidosis, with coma	2
E14.15	Unspecified diabetes mellitus with ketoacidosis and lactic acidosis, without coma	1

ICD-10-AM Code	Description	Number of Patients
E14.16	Unspecified diabetes mellitus with ketoacidosis and lactic acidosis, with coma	1
E14.22	Unspecified diabetes mellitus with established diabetic nephropathy	5
E14.23	Unspecified diabetes mellitus with end-stage renal disease	3
E14.31	Unspecified diabetes mellitus with background retinopathy	1
E14.34	Unspecified diabetes mellitus with other retinopathy	1
E14.40	Unspecified diabetes mellitus with neuropathy, unspecified	3
E14.42	Unspecified diabetes mellitus with diabetic polyneuropathy	3
E14.43	Unspecified diabetes mellitus with diabetic autonomic neuropathy	1
E14.52	Unspecified diabetes mellitus with peripheral angiopathy, with gangrene	10
E14.65	Unspecified diabetes mellitus with poor control	3
E14.71	Unspecified diabetes mellitus with multiple microvascular complications	1
E14.73	Unspecified diabetes mellitus with foot ulcer due to multiple causes	1
E14.8	Unspecified diabetes mellitus with unspecified complications	6
E14.9	Unspecified diabetes mellitus without complications	4
**Total**	**Patients with at least one of these codes**	**3,055**

Pre-existing diabetes was defined as any ICD-10-AM code E10-E14 among the discharge diagnoses; 3,055 patients had at least one such code. The table lists all 106 diabetes codes recorded in the cohort; no E12 code was recorded. Descriptions are English renderings of the code labels in the discharge records. ICD-10-AM, International Statistical Classification of Diseases and Related Health Problems, Tenth Revision, Australian Modification.

Early Glycemic Profile According to Diabetes Status

Table S3. Early glycemic profile according to diabetes status within the first 48 h.

Variable	Diabetes (*n* = 3,055)	No Diabetes (*n* = 7,777)	*p*
First glucose, mmol/L	9.9 (6.9-14.8)	6.4 (5.3-8.2)	<0.001
TWM-48h, mmol/L (available in 10,802)	9.7 (7.0-13.7)	6.4 (5.3-8.1)	<0.001
CV-48h (available in 7,978)	0.264 (0.148-0.422)	0.217 (0.120-0.364)	<0.001
Number of measurements, 48 h	2 (2-3)	2 (1-3)	<0.001
Mean glucose, mmol/L	9.7 (7.1-13.8)	6.4 (5.4-8.1)	<0.001
Minimum glucose, mmol/L	7.5 (5.3-10.8)	5.5 (4.5-6.7)	<0.001
Maximum glucose, mmol/L	11.8 (8.4-17.2)	7.4 (5.9-9.8)	<0.001
Hypoglycemia < 3.9 mmol/L (70 mg/dL)	337 (11.0%)	1,148 (14.8%)	<0.001
Hypoglycemia < 3.0 mmol/L (54 mg/dL)	171 (5.6%)	506 (6.5%)	0.079
Hyperglycemia > 10.0 mmol/L (180 mg/dL)	1,900 (62.2%)	1,810 (23.3%)	<0.001

Data are median (interquartile range) or *n* (%). Continuous variables were compared with the Mann-Whitney U test and categorical variables with the chi-square test. Glucose values were converted from mg/dL by dividing by 18.0. CV, coefficient of variation; TWM, time-weighted mean glucose.

Table S4. Early glycemic profile according to diabetes status within the first 72 h.

Variable	Diabetes (*n* = 3,055)	No Diabetes (*n* = 7,777)	*p*
First glucose, mmol/L	9.9 (6.9-14.8)	6.4 (5.3-8.2)	<0.001
TWM-72h, mmol/L (available in 10,802)	9.7 (7.0-13.3)	6.4 (5.3-8.0)	<0.001
CV-72h (available in 8,530)	0.281 (0.172-0.430)	0.231 (0.134-0.374)	<0.001
Number of measurements, 72 h	3 (2-4)	3 (2-4)	<0.001

Variable	Diabetes (*n* = 3,055)	No Diabetes (*n* = 7,777)	*p*
Mean glucose, mmol/L	9.8 (7.1-13.6)	6.4 (5.4-8.0)	<0.001
Minimum glucose, mmol/L	7.1 (5.1-9.9)	5.3 (4.3-6.4)	<0.001
Maximum glucose, mmol/L	12.4 (8.8-17.9)	7.6 (6.1-10.2)	<0.001
Hypoglycemia < 3.9 mmol/L (70 mg/dL)	380 (12.4%)	1,346 (17.3%)	<0.001
Hypoglycemia < 3.0 mmol/L (54 mg/dL)	189 (6.2%)	585 (7.5%)	0.015
Hyperglycemia > 10.0 mmol/L (180 mg/dL)	2,006 (65.7%)	2,023 (26.0%)	<0.001

Data are median (interquartile range) or *n* (%). Continuous variables were compared with the Mann-Whitney U test and categorical variables with the chi-square test. Glucose values were converted from mg/dL by dividing by 18.0. CV, coefficient of variation; TWM, time-weighted mean glucose.

Association Between Pre-Existing Diabetes and In-Hospital Mortality

Table S5. Full output of Model 2 (adjusted for year of admission, age, sex, malignancy, pneumonia, cirrhosis, CKD, COPD, and peritonitis; *N* = 10,832) and Model 3 (Model 2 plus hypoglycemia < 3.9 mmol/L, TWM-24h, CV-24h, and number of glucose measurements within 24 h; *N* = 6,434).

Predictor	Model 2 OR (95% CI)	*p*	Model 3 OR (95% CI)	*p*
Calendar year	0.964 (0.954-0.974)	<0.001	0.953 (0.939-0.966)	<0.001
Sex (male vs. female)	0.909 (0.837-0.986)	0.022	0.928 (0.835-1.031)	0.162
Age	1.040 (1.037-1.043)	<0.001	1.032 (1.029-1.036)	<0.001
Malignancy	1.559 (1.401-1.736)	<0.001	1.371 (1.185-1.585)	<0.001
Diabetes	0.822 (0.751-0.899)	<0.001	0.691 (0.610-0.784)	<0.001
Pneumonia	2.519 (2.262-2.806)	<0.001	2.526 (2.208-2.890)	<0.001
Cirrhosis	1.170 (0.951-1.439)	0.138	1.162 (0.895-1.510)	0.260
CKD	1.303 (1.162-1.462)	<0.001	1.287 (1.119-1.480)	<0.001
COPD	1.160 (0.975-1.380)	0.095	1.219 (0.980-1.517)	0.075
Peritonitis	0.760 (0.644-0.897)	0.001	0.922 (0.755-1.126)	0.426
Hypoglycemia < 3.9 mmol/L	—	—	1.680 (1.388-2.034)	<0.001
CV-24h (per 0.10)	—	—	1.051 (1.022-1.081)	<0.001
TWM-24h (per 1 mg/dL)	—	—	1.002 (1.001-1.003)	<0.001

Odds ratios for age and calendar year are per 1-year increment; for CV-24h, per 0.10 increase; for TWM-24h, per 1 mg/dL. Model 3 was also adjusted for the number of glucose measurements within 24 h; its coefficient is not shown. CI, confidence interval; CKD, chronic kidney disease; COPD, chronic obstructive pulmonary disease; CV, coefficient of variation; OR, odds ratio; TWM, time-weighted mean glucose.

Table S6. Association between pre-existing diabetes and in-hospital mortality across sequential logistic regression models, 48 h window.

Model	Covariate Adjustment	*N*	OR for Diabetes (95% CI)	*p*
Model 1	Unadjusted	10,832	0.866 (0.796-0.942)	<0.001
Model 2	Year of admission, age, sex, malignancy, pneumonia, cirrhosis, CKD, COPD, peritonitis	10,832	0.822 (0.751-0.899)	<0.001
Model 3	Model 2 + hypoglycemia < 3.9 mmol/L, TWM-48h, CV-48h, number of glucose measurements	7,957	0.665 (0.593-0.745)	<0.001
Model 4 (sensitivity)	Model 2 + mechanical ventilation, renal replacement therapy, surgical intervention	10,832	0.776 (0.705-0.854)	<0.001
Model 5 (sensitivity)	Model 4 + hypoglycemia < 3.9 mmol/L, TWM-48h, CV-48h, number of glucose measurements	7,957	0.649 (0.573-0.734)	<0.001

Models 1, 2, and 4 do not include glycemic variables and are therefore identical to those in Table 4 of the main text. CI, confidence interval; CKD, chronic kidney disease; COPD, chronic obstructive pulmonary disease; CV, coefficient of variation; OR, odds ratio; TWM, time-weighted mean glucose.

Table S7. Association between pre-existing diabetes and in-hospital mortality across sequential logistic regression models, 72 h window.

Model	Covariate Adjustment	*N*	OR for Diabetes (95% CI)	*p*
Model 1	Unadjusted	10,832	0.866 (0.796-0.942)	<0.001
Model 2	Year of admission, age, sex, malignancy, pneumonia, cirrhosis, CKD, COPD, peritonitis	10,832	0.822 (0.751-0.899)	<0.001
Model 3	Model 2 + hypoglycemia < 3.9 mmol/L, TWM-72h, CV-72h, number of glucose measurements	8,507	0.648 (0.580-0.724)	<0.001
Model 4 (sensitivity)	Model 2 + mechanical ventilation, renal replacement therapy, surgical intervention	10,832	0.776 (0.705-0.854)	<0.001
Model 5 (sensitivity)	Model 4 + hypoglycemia < 3.9 mmol/L, TWM-72h, CV-72h, number of glucose measurements	8,507	0.641 (0.567-0.724)	<0.001

Models 1, 2, and 4 do not include glycemic variables and are therefore identical to those in Table 4 of the main text. CI, confidence interval; CKD, chronic kidney disease; COPD, chronic obstructive pulmonary disease; CV, coefficient of variation; OR, odds ratio; TWM, time-weighted mean glucose.

Early Glycemic Parameters and In-Hospital Mortality

Table S8. Independent association of early glycemic parameters with in-hospital mortality in primary multivariable models, 24 h window. Each model included the glycemic parameter of interest, diabetes status, calendar year, sex, age, malignancy, pneumonia, cirrhosis, CKD, COPD, peritonitis, and the number of glucose measurements within the first 24 h.

Model	Glycemic Predictor	*N*	Adjusted OR (95% CI) for Predictor	*p*	Adjusted OR (95% CI) for Diabetes	*p*
A	TWM-24h (per 1 mg/dL)	10,802	1.002 (1.001-1.003)	<0.001	0.709 (0.642-0.783)	<0.001
B	CV-24h (per 0.10)	6,450	1.098 (1.072-1.125)	<0.001	0.787 (0.703-0.881)	<0.001
C	Hypoglycemia < 3.9 mmol/L	10,832	2.066 (1.792-2.382)	<0.001	0.831 (0.759-0.910)	<0.001
D	Hyperglycemia > 10.0 mmol/L	10,832	1.536 (1.392-1.695)	<0.001	0.700 (0.635-0.772)	<0.001

CV-24h per 0.10 increase; TWM-24h per 1 mg/dL. CI, confidence interval; CKD, chronic kidney disease; COPD, chronic obstructive pulmonary disease; CV, coefficient of variation; OR, odds ratio; TWM, time-weighted mean glucose.

Table S9. Missing data analysis: comparison of patients with available versus unavailable CV-24h.

Characteristic	CV-24h Available (*n* = 6,450)	CV-24h Unavailable (*n* = 4,382)	*p*
Age, years	72 (62-81)	71 (59-81)	<0.001
Male sex	3,179 (49.3%)	2,285 (52.1%)	0.003
Diabetes	1,995 (30.9%)	1,060 (24.2%)	<0.001
In-hospital mortality	3,481 (54.0%)	2,322 (53.0%)	0.316
Length of stay, days	9 (4-16)	6 (1-14)	<0.001
Malignancy	983 (15.2%)	883 (20.2%)	<0.001
CKD	1,125 (17.4%)	513 (11.7%)	<0.001
Cirrhosis	265 (4.1%)	169 (3.9%)	0.512
COPD	401 (6.2%)	225 (5.1%)	0.018

Characteristic	CV-24h Available (n = 6,450)	CV-24h Unavailable (n = 4,382)	p
Pneumonia	1,399 (21.7%)	777 (17.7%)	<0.001
Peritonitis	501 (7.8%)	241 (5.5%)	<0.001
Mechanical ventilation	1,829 (28.4%)	490 (11.2%)	<0.001
Renal replacement therapy	421 (6.5%)	261 (6.0%)	0.230
Surgical intervention	1,552 (24.1%)	1,123 (25.6%)	0.064
First glucose, mmol/L	7.2 (5.7-9.9)	6.6 (5.4-9.1)	<0.001
Number of glucose measurements, 24 h	2 (2-3)	1 (1-1)	<0.001

Data are median (interquartile range) or n (%). CV-24h requires at least two glucose measurements within the first 24 h. Availability of CV-24h depends on survival to a second measurement (Tables S23-S25 and Table 6). CKD, chronic kidney disease; COPD, chronic obstructive pulmonary disease; CV, coefficient of variation.

Table S10. Independent association of early glycemic parameters with in-hospital mortality, sensitivity analyses, 24 h window. Each model included the glycemic parameter of interest, diabetes status, calendar year, sex, age, malignancy, pneumonia, cirrhosis, CKD, COPD, peritonitis, and the number of glucose measurements within the first 24 h, additionally adjusted for mechanical ventilation, renal replacement therapy, and surgical intervention.

Model	Glycemic Predictor	N	Adjusted OR (95% CI) for Predictor	p	Adjusted OR (95% CI) for Diabetes	p
A	TWM-24h (per 1 mg/dL)	10,802	1.002 (1.002-1.003)	<0.001	0.679 (0.611-0.756)	<0.001
B	CV-24h (per 0.10)	6,450	1.088 (1.060-1.117)	<0.001	0.782 (0.692-0.883)	<0.001
C	Hypoglycemia < 3.9 mmol/L	10,832	2.059 (1.768-2.397)	<0.001	0.806 (0.731-0.888)	<0.001
D	Hyperglycemia > 10.0 mmol/L	10,832	1.514 (1.362-1.682)	<0.001	0.685 (0.617-0.760)	<0.001

CV-24h per 0.10 increase; TWM-24h per 1 mg/dL. CI, confidence interval; CKD, chronic kidney disease; COPD, chronic obstructive pulmonary disease; CV, coefficient of variation; OR, odds ratio; TWM, time-weighted mean glucose.

Table S11. Independent association of early glycemic parameters with in-hospital mortality in primary multivariable models, 48 h window. Each model included the glycemic parameter of interest, diabetes status, calendar year, sex, age, malignancy, pneumonia, cirrhosis, CKD, COPD, peritonitis, and the number of glucose measurements within the first 48 h.

Model	Glycemic Predictor	N	Adjusted OR (95% CI) for Predictor	p	Adjusted OR (95% CI) for Diabetes	p
A	TWM-48h (per 1 mg/dL)	10,802	1.003 (1.002-1.004)	<0.001	0.677 (0.613-0.748)	<0.001
B	CV-48h (per 0.10)	7,978	1.101 (1.076-1.126)	<0.001	0.789 (0.712-0.874)	<0.001
C	Hypoglycemia < 3.9 mmol/L	10,832	1.740 (1.539-1.966)	<0.001	0.845 (0.772-0.925)	<0.001
D	Hyperglycemia > 10.0 mmol/L	10,832	1.643 (1.492-1.810)	<0.001	0.688 (0.624-0.758)	<0.001

CV-48h per 0.10 increase; TWM-48h per 1 mg/dL. CI, confidence interval; CKD, chronic kidney disease; COPD, chronic obstructive pulmonary disease; CV, coefficient of variation; OR, odds ratio; TWM, time-weighted mean glucose.

Table S12. Independent association of early glycemic parameters with in-hospital mortality, sensitivity analyses, 48 h window. Each model included the glycemic parameter of interest, diabetes status, calendar year, sex, age, malignancy, pneumonia, cirrhosis, CKD, COPD, peritonitis, and the number of glucose measurements within the first 48 h, additionally adjusted for mechanical ventilation, renal replacement therapy, and surgical intervention.

Model	Glycemic Predictor	N	Adjusted OR (95% CI) for Predictor	p	Adjusted OR (95% CI) for Diabetes	p
A	TWM-48h (per 1 mg/dL)	10,802	1.003 (1.002-1.004)	<0.001	0.661 (0.594-0.736)	<0.001
B	CV-48h (per 0.10)	7,978	1.088 (1.062-1.115)	<0.001	0.780 (0.698-0.871)	<0.001

Model	Glycemic Predictor	*N*	Adjusted OR (95% CI) for Predictor	*p*	Adjusted OR (95% CI) for Diabetes	*p*
C	Hypoglycemia < 3.9 mmol/L	10,832	1.744 (1.527-1.991)	<0.001	0.834 (0.756-0.920)	<0.001
D	Hyperglycemia > 10.0 mmol/L	10,832	1.602 (1.444-1.777)	<0.001	0.687 (0.618-0.763)	<0.001

CV-48h per 0.10 increase; TWM-48h per 1 mg/dL. CI, confidence interval; CKD, chronic kidney disease; COPD, chronic obstructive pulmonary disease; CV, coefficient of variation; OR, odds ratio; TWM, time-weighted mean glucose.

Table S13. Independent association of early glycemic parameters with in-hospital mortality in primary multivariable models, 72 h window. Each model included the glycemic parameter of interest, diabetes status, calendar year, sex, age, malignancy, pneumonia, cirrhosis, CKD, COPD, peritonitis, and the number of glucose measurements within the first 72 h.

Model	Glycemic Predictor	*N*	Adjusted OR (95% CI) for Predictor	*p*	Adjusted OR (95% CI) for Diabetes	*p*
A	TWM-72h (per 1 mg/dL)	10,802	1.004 (1.003-1.004)	<0.001	0.659 (0.596-0.728)	<0.001
B	CV-72h (per 0.10)	8,530	1.115 (1.090-1.141)	<0.001	0.793 (0.718-0.876)	<0.001
C	Hypoglycemia < 3.9 mmol/L	10,832	1.659 (1.479-1.861)	<0.001	0.859 (0.785-0.941)	0.001
D	Hyperglycemia > 10.0 mmol/L	10,832	1.743 (1.584-1.918)	<0.001	0.681 (0.618-0.751)	<0.001

CV-72h per 0.10 increase; TWM-72h per 1 mg/dL. CI, confidence interval; CKD, chronic kidney disease; COPD, chronic obstructive pulmonary disease; CV, coefficient of variation; OR, odds ratio; TWM, time-weighted mean glucose.

Table S14. Independent association of early glycemic parameters with in-hospital mortality, sensitivity analyses, 72 h window. Each model included the glycemic parameter of interest, diabetes status, calendar year, sex, age, malignancy, pneumonia, cirrhosis, CKD, COPD, peritonitis, and the number of glucose measurements within the first 72 h, additionally adjusted for mechanical ventilation, renal replacement therapy, and surgical intervention.

Model	Glycemic Predictor	*N*	Adjusted OR (95% CI) for Predictor	*p*	Adjusted OR (95% CI) for Diabetes	*p*
A	TWM-72h (per 1 mg/dL)	10,802	1.004 (1.003-1.005)	<0.001	0.649 (0.582-0.723)	<0.001
B	CV-72h (per 0.10)	8,530	1.103 (1.076-1.130)	<0.001	0.793 (0.711-0.884)	<0.001
C	Hypoglycemia < 3.9 mmol/L	10,832	1.684 (1.485-1.909)	<0.001	0.855 (0.775-0.943)	0.002
D	Hyperglycemia > 10.0 mmol/L	10,832	1.689 (1.523-1.874)	<0.001	0.685 (0.616-0.762)	<0.001

CV-72h per 0.10 increase; TWM-72h per 1 mg/dL. CI, confidence interval; CKD, chronic kidney disease; COPD, chronic obstructive pulmonary disease; CV, coefficient of variation; OR, odds ratio; TWM, time-weighted mean glucose.

Effect Modification by Pre-Existing Diabetes

Table S15. Interaction between pre-existing diabetes and early glycemic parameters, sensitivity analysis, 24 h window. Models were adjusted for calendar year, sex, age, malignancy, pneumonia, cirrhosis, CKD, COPD, peritonitis, and number of glucose measurements within the first 24 h, and additionally for mechanical ventilation, renal replacement therapy, and surgical intervention. Hospital length of stay was not included because it is a post-baseline variable.

Model	Variable	*N*	OR (95% CI)	*p*	*p* for Interaction
A	TWM-24h (per 1 mg/dL)	10,802	1.003 (1.002-1.004)	<0.001	
	Diabetes		0.822 (0.660-1.025)	0.081	
	TWM-24h × diabetes		0.999 (0.998-1.000)		0.053
B	CV-24h (per 0.10)	6,450	1.115 (1.079-1.152)	<0.001	

Model	Variable	*N*	OR (95% CI)	*p*	*p* for Interaction
	Diabetes		0.933 (0.773-1.127)	0.473	
	CV-24h × diabetes		0.936 (0.887-0.987)		0.015
C	Hypoglycemia < 3.9 mmol/L	10,832	2.221 (1.858-2.655)	<0.001	
	Diabetes		0.826 (0.746-0.914)	<0.001	
	Hypoglycemia × diabetes		0.754 (0.537-1.058)		0.102
D	Hyperglycemia > 10.0 mmol/L	10,832	1.643 (1.434-1.882)	<0.001	
	Diabetes		0.745 (0.650-0.853)	<0.001	
	Hyperglycemia × diabetes		0.817 (0.662-1.008)		0.059

CV-24h and its interaction term per 0.10 increase; TWM-24h and its interaction term per 1 mg/dL. CI, confidence interval; CKD, chronic kidney disease; COPD, chronic obstructive pulmonary disease; CV, coefficient of variation; OR, odds ratio; TWM, time-weighted mean glucose.

Table S16. Interaction between pre-existing diabetes and early glycemic parameters, primary analysis, 48 h window. Models were adjusted for calendar year, sex, age, malignancy, pneumonia, cirrhosis, CKD, COPD, peritonitis, and number of glucose measurements within the first 48 h. Hospital length of stay was not included because it is a post-baseline variable.

Model	Variable	*N*	OR (95% CI)	*p*	*p* for Interaction
A	TWM-48h (per 1 mg/dL)	10,802	1.004 (1.003-1.006)	<0.001	
	Diabetes		0.973 (0.784-1.209)	0.807	
	TWM-48h × diabetes		0.998 (0.996-0.999)		<0.001
B	CV-48h (per 0.10)	7,978	1.128 (1.096-1.160)	<0.001	
	Diabetes		0.974 (0.820-1.157)	0.763	
	CV-48h × diabetes		0.930 (0.887-0.975)		0.003
C	Hypoglycemia < 3.9 mmol/L	10,832	1.790 (1.554-2.061)	<0.001	
	Diabetes		0.856 (0.778-0.942)	0.001	
	Hypoglycemia × diabetes		0.893 (0.675-1.180)		0.425
D	Hyperglycemia > 10.0 mmol/L	10,832	1.789 (1.586-2.018)	<0.001	
	Diabetes		0.767 (0.671-0.877)	<0.001	
	Hyperglycemia × diabetes		0.794 (0.655-0.963)		0.019

CV-48h and its interaction term per 0.10 increase; TWM-48h and its interaction term per 1 mg/dL. CI, confidence interval; CKD, chronic kidney disease; COPD, chronic obstructive pulmonary disease; CV, coefficient of variation; OR, odds ratio; TWM, time-weighted mean glucose.

Table S17. Interaction between pre-existing diabetes and early glycemic parameters, sensitivity analysis, 48 h window. Models were adjusted for calendar year, sex, age, malignancy, pneumonia, cirrhosis, CKD, COPD, peritonitis, and number of glucose measurements within the first 48 h, and additionally for mechanical ventilation, renal replacement therapy, and surgical intervention. Hospital length of stay was not included because it is a post-baseline variable.

Model	Variable	*N*	OR (95% CI)	*p*	*p* for Interaction
A	TWM-48h (per 1 mg/dL)	10,802	1.004 (1.003-1.005)	<0.001	
	Diabetes		0.831 (0.658-1.049)	0.119	
	TWM-48h × diabetes		0.999 (0.997-1.000)		0.031
B	CV-48h (per 0.10)	7,978	1.107 (1.074-1.140)	<0.001	
	Diabetes		0.900 (0.748-1.084)	0.266	
	CV-48h × diabetes		0.952 (0.904-1.002)		0.059
C	Hypoglycemia < 3.9 mmol/L	10,832	1.806 (1.550-2.105)	<0.001	

Model	Variable	N	OR (95% CI)	p	p for Interaction
	Diabetes		0.848 (0.764-0.940)	0.002	
	Hypoglycemia × diabetes		0.868 (0.642-1.174)		0.359
D	Hyperglycemia > 10.0 mmol/L	10,832	1.675 (1.472-1.906)	<0.001	
	Diabetes		0.728 (0.630-0.841)	<0.001	
	Hyperglycemia × diabetes		0.885 (0.718-1.090)		0.250

CV-48h and its interaction term per 0.10 increase; TWM-48h and its interaction term per 1 mg/dL. CI, confidence interval; CKD, chronic kidney disease; COPD, chronic obstructive pulmonary disease; CV, coefficient of variation; OR, odds ratio; TWM, time-weighted mean glucose.

Table S18. Interaction between pre-existing diabetes and early glycemic parameters, primary analysis, 72 h window. Models were adjusted for calendar year, sex, age, malignancy, pneumonia, cirrhosis, CKD, COPD, peritonitis, and number of glucose measurements within the first 72 h. Hospital length of stay was not included because it is a post-baseline variable.

Model	Variable	N	OR (95% CI)	p	p for Interaction
A	TWM-72h (per 1 mg/dL)	10,802	1.005 (1.004-1.006)	<0.001	
	Diabetes		0.979 (0.782-1.224)	0.850	
	TWM-72h × diabetes		0.997 (0.996-0.999)		<0.001
B	CV-72h (per 0.10)	8,530	1.141 (1.110-1.172)	<0.001	
	Diabetes		0.993 (0.832-1.186)	0.939	
	CV-72h × diabetes		0.929 (0.885-0.975)		0.003
C	Hypoglycemia < 3.9 mmol/L	10,832	1.698 (1.489-1.938)	<0.001	
	Diabetes		0.870 (0.790-0.959)	0.005	
	Hypoglycemia × diabetes		0.910 (0.700-1.182)		0.478
D	Hyperglycemia > 10.0 mmol/L	10,832	1.891 (1.683-2.125)	<0.001	
	Diabetes		0.771 (0.670-0.886)	<0.001	
	Hyperglycemia × diabetes		0.788 (0.649-0.956)		0.016

CV-72h and its interaction term per 0.10 increase; TWM-72h and its interaction term per 1 mg/dL. CI, confidence interval; CKD, chronic kidney disease; COPD, chronic obstructive pulmonary disease; CV, coefficient of variation; OR, odds ratio; TWM, time-weighted mean glucose.

Table S19. Interaction between pre-existing diabetes and early glycemic parameters, sensitivity analysis, 72 h window. Models were adjusted for calendar year, sex, age, malignancy, pneumonia, cirrhosis, CKD, COPD, peritonitis, and number of glucose measurements within the first 72 h, and additionally for mechanical ventilation, renal replacement therapy, and surgical intervention. Hospital length of stay was not included because it is a post-baseline variable.

Model	Variable	N	OR (95% CI)	p	p for Interaction
A	TWM-72h (per 1 mg/dL)	10,802	1.005 (1.004-1.006)	<0.001	
	Diabetes		0.841 (0.660-1.071)	0.160	
	TWM-72h × diabetes		0.998 (0.997-1.000)		0.019
B	CV-72h (per 0.10)	8,530	1.119 (1.087-1.152)	<0.001	
	Diabetes		0.917 (0.756-1.111)	0.375	
	CV-72h × diabetes		0.953 (0.905-1.005)		0.074
C	Hypoglycemia < 3.9 mmol/L	10,832	1.738 (1.505-2.007)	<0.001	
	Diabetes		0.869 (0.783-0.966)	0.009	
	Hypoglycemia × diabetes		0.878 (0.659-1.170)		0.376
D	Hyperglycemia > 10.0 mmol/L	10,832	1.755 (1.548-1.990)	<0.001	

Model	Variable	*N*	OR (95% CI)	*p*	*p* for Interaction
	Diabetes		0.726 (0.624-0.845)	<0.001	
	Hyperglycemia × diabetes		0.894 (0.724-1.102)		0.293

CV-72h and its interaction term per 0.10 increase; TWM-72h and its interaction term per 1 mg/dL. CI, confidence interval; CKD, chronic kidney disease; COPD, chronic obstructive pulmonary disease; CV, coefficient of variation; OR, odds ratio; TWM, time-weighted mean glucose.

Table S20. Diabetes-stratified association between early glycemic variability and in-hospital mortality, 48 h window. Primary models were adjusted for calendar year, age, sex, malignancy, pneumonia, cirrhosis, CKD, COPD, peritonitis, and the number of glucose measurements within the first 48 h; sensitivity models were additionally adjusted for mechanical ventilation, renal replacement therapy, and surgical intervention. CV-48h was modeled as a continuous variable; an OR > 1 indicates higher odds of in-hospital death with increasing early glycemic variability.

Cohort	Model	*N*	Adjusted OR (95% CI) per 0.10 Increase in CV-48h	*p*
No diabetes	Primary	5,539	1.131 (1.100-1.164)	<0.001
No diabetes	Sensitivity	5,539	1.111 (1.078-1.145)	<0.001
Diabetes	Primary	2,439	1.043 (1.003-1.085)	0.033
Diabetes	Sensitivity	2,439	1.046 (1.002-1.092)	0.038

CI, confidence interval; CKD, chronic kidney disease; COPD, chronic obstructive pulmonary disease; CV, coefficient of variation; OR, odds ratio.

Table S21. Diabetes-stratified association between early glycemic variability and in-hospital mortality, 72 h window. Primary models were adjusted for calendar year, age, sex, malignancy, pneumonia, cirrhosis, CKD, COPD, peritonitis, and the number of glucose measurements within the first 72 h; sensitivity models were additionally adjusted for mechanical ventilation, renal replacement therapy, and surgical intervention. CV-72h was modeled as a continuous variable; an OR > 1 indicates higher odds of in-hospital death with increasing early glycemic variability.

Cohort	Model	*N*	Adjusted OR (95% CI) per 0.10 Increase in CV-72h	*p*
No diabetes	Primary	5,942	1.145 (1.114-1.177)	<0.001
No diabetes	Sensitivity	5,942	1.122 (1.090-1.156)	<0.001
Diabetes	Primary	2,588	1.053 (1.011-1.097)	0.012
Diabetes	Sensitivity	2,588	1.059 (1.013-1.107)	0.012

CI, confidence interval; CKD, chronic kidney disease; COPD, chronic obstructive pulmonary disease; CV, coefficient of variation; OR, odds ratio.

Comparison of Published Meta-Analyses

Table S22. Comparison of key characteristics and findings of the 2022 (Jiang and Cheng [1]) and 2026 (Zheng et al. [2]) meta-analyses on the association between diabetes and mortality in sepsis.

Comparison Point	2022 Meta-Analysis (Jiang and Cheng) [1]	2026 Meta-Analysis (Zheng et al.) [2]
Included literature	21 studies after screening 4,108 unique citations	13 studies after screening 8,374 records
Populations and sepsis severity	Mixed: 10 studies enrolled severe sepsis and/or septic shock; 11 included sepsis of varied stages	Patients with sepsis admitted to the ICU
Mortality outcome definitions	In-hospital and latest-follow-up mortality (up to 90 days); diabetes not significantly associated with either: in-hospital RR 0.98 (0.93-1.04), I^2 = 72.1%, *N* = 18; latest follow-up RR 0.94 (0.86-1.04), I^2 = 5.4%, *N* = 6	Overall pooled mortality RR 1.25 (1.14-1.38); subgroups: in-hospital RR 1.60 (1.11-2.31), *n* = 5; 28-day RR 1.34 (0.87-2.07), *n* = 5; 90-day RR 1.10 (0.88-1.36), *n* = 5

Comparison Point	2022 Meta-Analysis (Jiang and Cheng) [1]	2026 Meta-Analysis (Zheng et al.) [2]
Heterogeneity	Moderate-to-high for in-hospital mortality (I^2 = 72.1%); low for later mortality (I^2 = 5.4%)	Extreme for overall mortality (I^2 = 98.6%, p = 0.001)
Publication bias	Egger's test not indicative (p = 0.23 in-hospital; p = 0.49 latest follow-up)	Funnel-plot asymmetry and Egger's p = 0.003; trim-and-fill applied
Geographic origin	USA (5), Greece (3), Spain (2), Taiwan (2), China (1), Israel (1), Netherlands (1), Japan (1), Singapore (1), France (1), South Korea (1), plus 2 multicenter studies	China (3), Greece (2), USA (2), Netherlands (2), Japan (1), Canada (1), France (1), Israel (1)
Diabetes vs. acute hyperglycemia	Separated diabetes from admission glucose; diabetes not associated with mortality, but high admission glucose was: RR 1.45 (1.01-2.09), I^2 = 76.5%, N = 6	Focused on diabetes as exposure; did not address admission hyperglycemia; most studies lacked detailed adjustment for glycemic control
Residual confounding/case-mix	Diabetes not associated with poor survival; more well-designed adjusted studies needed; glycemic control could not be analyzed	Most studies inadequately adjusted for glycemic control, cardiovascular/kidney disease, infection type, and severity; observational data limit causal inference

ICU, intensive care unit; RR, risk ratio.

Informative Observation and Density of Glucose Sampling

Table S23. In-hospital mortality according to the number of glucose measurements within the first 24 h.

Measurements in 24 h	*N*	Deaths	Mortality	No diabetes, mortality (deaths/*N*)	Diabetes, mortality (deaths/*N*)
1	4,377	2,320	53.0%	53.4% (1,774/3,319)	51.6% (546/1,058)
2	4,618	2,483	53.8%	55.7% (1,786/3,207)	49.4% (697/1,411)
3	1,470	810	55.1%	55.4% (563/1,017)	54.5% (247/453)
≥4	367	190	51.8%	52.1% (122/234)	51.1% (68/133)

Adjusted OR for death per additional measurement 1.038 (0.988-1.091), from a logistic model including the baseline covariates of Model 2 (calendar year, age, sex, malignancy, pneumonia, cirrhosis, CKD, COPD, peritonitis); with diabetes added as a covariate, 1.047 (0.996-1.101). CKD, chronic kidney disease; COPD, chronic obstructive pulmonary disease; OR, odds ratio.

Table S24. Timing and availability of the second glucose measurement according to diabetes status and timing of death.

Group	*N*	Second measurement <6 h	<12 h	<18 h	<24 h	Never	CV-24h available	Median measurements in 24 h
All patients	10,832	7.9%	27.0%	45.6%	59.5%	14.8%	59.5%	2
No diabetes	7,777	7.5%	26.1%	44.1%	57.2%	16.7%	57.3%	2
Diabetes	3,055	9.0%	29.5%	49.5%	65.3%	10.0%	65.3%	2
Death ≤24 h after admission	1,450	5.9%	20.6%	29.1%	31.9%	66.9%	32.1%	1
Death 24-48 h	598	11.4%	37.6%	57.2%	75.8%	14.2%	75.9%	2
Death 2-7 days	1,463	8.5%	30.3%	53.5%	70.9%	3.1%	70.9%	2
Death >7 days	2,292	8.7%	29.8%	48.7%	66.5%	0.4%	66.5%	2
Death >48 h (all)	3,755	8.7%	30.0%	50.6%	68.2%	1.5%	68.2%	2
Survivors	5,029	7.5%	25.4%	45.2%	59.0%	9.8%	59.0%	2

Times of the second measurement are measured from the first glucose measurement; timing of death is measured from the admission timestamp. Denominators are the full group. Interval from the first to the second measurement, all patients with ≥2 measurements (n = 9,229): median (IQR) 17.0 (10.3-30.1) h; among patients with a second value within 24 h (n = 6,447): median (IQR) 12.8 (8.3-17.7) h. In total, 1,289 patients (11.9%) left the hospital (1,216 by death) less than 24 h after the first measurement; 327 of them (25.4%) had a computable CV-24h. In 3 patients with a computable CV-24h, the second value within 24 h carried the same timestamp as the first and is not counted as a later measurement. CV, coefficient of variation; IQR, interquartile range.

Table S25. Diabetes-stratified association between early glycemic variability and in-hospital mortality in landmark cohorts (detail of Table 6).

Landmark cohort	Exposure	Stratum	*N*	Adjusted OR per 0.10 (95% CI)	*p*
Alive at 24 h	CV-24h	No diabetes	4,197	1.092 (1.056-1.128)	<0.001
Alive at 24 h	CV-24h	Diabetes	1,926	1.023 (0.982-1.066)	0.271
Alive at 48 h	CV-24h	No diabetes	3,769	1.049 (1.012-1.088)	0.009
Alive at 48 h	CV-24h	Diabetes	1,770	1.022 (0.979-1.067)	0.312
Alive at 72 h	CV-24h	No diabetes	3,529	1.042 (1.004-1.083)	0.031
Alive at 72 h	CV-24h	Diabetes	1,654	1.017 (0.972-1.064)	0.470
Alive at 48 h	CV-48h	No diabetes	4,812	1.046 (1.012-1.081)	0.008
Alive at 48 h	CV-48h	Diabetes	2,198	1.028 (0.985-1.073)	0.205
Alive at 72 h	CV-72h	No diabetes	4,874	1.055 (1.021-1.090)	0.001
Alive at 72 h	CV-72h	Diabetes	2,205	1.024 (0.976-1.073)	0.334

Models adjusted for calendar year, age, sex, malignancy, pneumonia, cirrhosis, CKD, COPD, peritonitis and number of glucose measurements within the exposure window, as in Table 6. Landmark cohorts comprise patients alive and hospitalized at the stated time after the first glucose measurement, with a computable CV for the stated exposure. In-hospital mortality in the landmark cohorts (all patients, irrespective of CV availability): 48.1% (4,587/9,543) at 24 h and 44.6% (3,878/8,697) at 48 h. CI, confidence interval; CKD, chronic kidney disease; COPD, chronic obstructive pulmonary disease; CV, coefficient of variation; OR, odds ratio. Of the 6,450 patients with CV-24h (3,481 deaths), 327 (310 deaths) had died or been discharged before 24 h, 911 (844 deaths) before 48 h and 1,267 (1,136 deaths) before 72 h after the first glucose measurement; of the 7,978 patients with CV-48h (4,179 deaths), 968 (883 deaths) left before 48 h, and of the 8,530 with CV-72h (4,400 deaths), 1,451 (1,267 deaths) left before 72 h.

Table S26. Sensitivity of the diabetes-by-CV interaction and of the diabetes-stratified associations to the minimum number of glucose measurements within the exposure window.

Window, minimum measurements	*N*	*N*, no diabetes / diabetes	Mortality	Interaction OR per 0.10 (95% CI)	*p*	CV OR per 0.10, no diabetes (95% CI)	*p*	CV OR per 0.10, diabetes (95% CI)	*p*
24 h, ≥2	6,450	4,455 / 1,995	54.0%	0.912 (0.868-0.959)	<0.001	1.137 (1.102-1.173)	<0.001	1.034 (0.994-1.076)	0.099
24 h, ≥3	1,837	1,251 / 586	54.4%	0.911 (0.825-1.006)	0.065	1.154 (1.086-1.226)	<0.001	1.044 (0.964-1.131)	0.294
24 h, ≥4	367	234 / 133	51.8%	0.823 (0.658-1.029)	0.087	1.232 (1.053-1.442)	0.009	1.007 (0.846-1.198)	0.941
48 h, ≥2	7,978	5,539 / 2,439	52.4%	0.930 (0.887-0.975)	0.003	1.131 (1.100-1.164)	<0.001	1.043 (1.003-1.085)	0.033
48 h, ≥3	4,665	3,153 / 1,512	52.8%	0.915 (0.858-0.976)	0.007	1.110 (1.067-1.154)	<0.001	1.012 (0.960-1.066)	0.662
48 h, ≥4	1,940	1,290 / 650	54.3%	0.933 (0.843-1.032)	0.176	1.121 (1.053-1.192)	<0.001	1.039 (0.958-1.128)	0.354
48 h, ≥5	684	443 / 241	52.9%	0.850 (0.709-1.019)	0.079	1.166 (1.043-1.304)	0.007	0.986 (0.847-1.147)	0.854
72 h, ≥2	8,530	5,942 / 2,588	51.6%	0.929 (0.885-0.975)	0.003	1.145 (1.114-1.177)	<0.001	1.053 (1.011-1.097)	0.012
72 h, ≥3	5,951	4,029 / 1,922	51.4%	0.950 (0.894-1.009)	0.096	1.122 (1.083-1.162)	<0.001	1.056 (1.004-1.111)	0.033
72 h, ≥4	3,710	2,469 / 1,241	52.1%	0.955 (0.884-1.032)	0.243	1.116 (1.066-1.168)	<0.001	1.063 (0.997-1.132)	0.061
72 h, ≥5	1,835	1,202 / 633	53.2%	0.952 (0.853-1.063)	0.381	1.092 (1.022-1.167)	0.009	1.038 (0.950-1.135)	0.410

All models adjusted for calendar year, age, sex, malignancy, pneumonia, cirrhosis, CKD, COPD, peritonitis, and number of glucose measurements within the window. Median CV-24h with ≥2, ≥3 and ≥4 measurements in 24 h: 0.193, 0.246 and 0.308 in patients without diabetes; 0.229, 0.282 and 0.331 in patients with diabetes. Among patients with computable CV-24h, 4,613 had 2 measurements, 1,470 had 3, 296 had 4 and 71 had 5 or more. CV-by-measurement-count interaction (CV per 0.10) in the 24 h stratified models: no diabetes OR 1.023 (0.972-1.077), $p = 0.389$; diabetes OR 0.988 (0.932-1.048), $p = 0.697$. CI, confidence interval; CKD, chronic kidney disease; COPD, chronic obstructive pulmonary disease; CV, coefficient of variation; OR, odds ratio.

Anchoring of the Exposure Window

Table S27. Timing of the first glucose measurement relative to hospital admission (Panel A), and sensitivity of the principal findings to the anchor of the exposure window (Panel B).

Panel A. Interval from the administrative admission timestamp to the first glucose measurement (negative values indicate measurements obtained before admission).

Group	*N*	Median (IQR), h	Before admission	*p*
All patients	10,832	−3.8 (−6.7 to −1.7)	86.6%	
Survivors	5,029	−3.6 (−6.7 to −1.0)	81.9%	<0.001
Non-survivors	5,803	−4.1 (−6.7 to −2.1)	90.7%	
Death within 24 h of admission	1,450	−4.1 (−6.3 to −2.4)	97.2%	
No diabetes	7,777	−3.7 (−6.6 to −1.5)	85.7%	<0.001
Diabetes	3,055	−4.1 (−7.0 to −2.0)	88.9%	

Distribution of the interval, all patients: more than 24 h before admission 1.1%; 6-24 h before 28.6%; 0-6 h before 56.9%; 0-6 h after 10.3%; 6-24 h after 1.7%; more than 24 h after 1.3%. *p* values from the Mann-Whitney U test (survivors vs. non-survivors; patients without vs. with diabetes).

Panel B. Principal models with the exposure window anchored to hospital admission.

Window	Patients with ≥1 value	Patients with CV	Median measurements	Interaction OR per 0.10 (95% CI), *p*	CV OR per 0.10, no diabetes (95% CI)	CV OR per 0.10, diabetes (95% CI)	Hypoglycemia OR (95% CI)	Hyperglycemia OR (95% CI)	Diabetes OR, Model 3-type (95% CI)
First glucose to +24 h (primary)	10,832	6,450	2	0.912 (0.868-0.959), <0.001	1.137 (1.102-1.173)	1.034 (0.994-1.076)	2.066 (1.792-2.382)	1.536 (1.392-1.695)	0.691 (0.610-0.784)
Admission to +24 h	7,698	2,408	1	0.918 (0.847-0.995), 0.037	1.103 (1.050-1.158)	1.017 (0.952-1.086)	1.441 (1.217-1.707)	1.651 (1.466-1.860)	0.752 (0.613-0.923)
Admission −24 h to +24 h	10,674	6,839	2	0.920 (0.877-0.966), <0.001	1.134 (1.101-1.169)	1.040 (1.000-1.081)	1.987 (1.730-2.281)	1.576 (1.428-1.739)	0.656 (0.581-0.742)
Admission to +48 h	8,636	5,084	2	0.957 (0.902-1.014), 0.137	1.099 (1.063-1.137)	1.050 (1.000-1.102)	1.365 (1.189-1.567)	1.742 (1.561-1.944)	0.728 (0.634-0.837)

Panel B: glycemic metrics were recomputed from the individual glucose records within each window. All models adjusted for calendar year, age, sex, malignancy, pneumonia, cirrhosis, CKD, COPD, peritonitis, and number of glucose measurements within the window; hypoglycemia and hyperglycemia models additionally adjusted for diabetes status and fitted in patients with at least one value. The Model 3-type diabetes OR is from a model including the Model 2 covariates, hypoglycemia, CV, measurement count and TWM-24h (primary window) or mean glucose (admission-anchored windows), fitted in patients with computable CV. In the window from admission to +24 h, 3,134 patients (28.9%) had no glucose value. Hypoglycemia < 3.9 mmol/L (70 mg/dL); hyperglycemia > 10.0 mmol/L (180 mg/dL). CI, confidence interval; CKD, chronic kidney disease; COPD, chronic obstructive pulmonary disease; CV, coefficient of variation; IQR, interquartile range; OR, odds ratio; TWM, time-weighted mean glucose.

Sepsis Ascertainment and Coded Acute Organ Dysfunction

Table S28. Sensitivity of the principal findings to the stringency of sepsis ascertainment (Panel A), and in-hospital mortality according to the route of sepsis identification (Panel B).

Panel A. Principal models in ascertainment subgroups.

Subgroup	*N*	Mortality	*N* with CV-24h (no diabetes / diabetes)	Interaction OR per 0.10 (95% CI), *p*	CV OR per 0.10, no diabetes (95% CI)	CV OR per 0.10, diabetes (95% CI)	Hypoglycemia OR (95% CI)	Hyperglycemia OR (95% CI)	Diabetes OR, Model 2 (95% CI)
Full cohort	10,832	53.6%	6,450 (4,455 / 1,995)	0.912 (0.868-0.959), <0.001	1.137 (1.102-1.173)	1.034 (0.994-1.076)	2.066 (1.792-2.382)	1.536 (1.392-1.695)	0.822 (0.751-0.899)
A. Any explicit sepsis code	10,380	53.4%	6,114 (4,229 / 1,885)	0.911 (0.865-0.959), <0.001	1.140 (1.104-1.177)	1.038 (0.996-1.082)	2.111 (1.826-2.442)	1.566 (1.416-1.733)	0.813 (0.741-0.890)
B. Sepsis code as principal diagnosis	4,360	54.7%	2,599 (1,792 / 807)	0.896 (0.830-0.967), 0.005	1.150 (1.097-1.205)	1.028 (0.967-1.093)	1.877 (1.530-2.303)	1.786 (1.536-2.078)	0.727 (0.635-0.833)
C. Sepsis code + coded organ dysfunction	6,140	69.1%	3,956 (2,773 / 1,183)	0.899 (0.843-0.959), 0.001	1.148 (1.102-1.195)	1.028 (0.977-1.082)	1.827 (1.518-2.199)	1.613 (1.409-1.848)	0.828 (0.731-0.937)
D. Septic shock in discharge text	1,154	73.1%	712 (527 / 185)	0.809 (0.684-0.957), 0.014	1.208 (1.094-1.334)	0.997 (0.866-1.149)	1.680 (1.118-2.525)	1.436 (1.022-2.017)	0.864 (0.627-1.191)

Subgroup	*N*	Mortality	*N* with CV-24h (no diabetes / diabetes)	Interaction OR per 0.10 (95% CI), *p*	CV OR per 0.10, no diabetes (95% CI)	CV OR per 0.10, diabetes (95% CI)	Hypoglycemia OR (95% CI)	Hyperglycemia OR (95% CI)	Diabetes OR, Model 2 (95% CI)
E. Sepsis code without coded organ dysfunction	4,240	30.7%	2,158 (1,456 / 702)	0.940 (0.844-1.046), 0.256	1.048 (0.982-1.119)	0.985 (0.903-1.076)	1.807 (1.369-2.387)	1.382 (1.154-1.655)	0.849 (0.723-0.997)

Subgroups A-D are the sensitivity analyses; E is a contrast group. Coded acute organ dysfunction (subgroups C and E; codes only): J96.0, J96.9, J80; R57.x; N17.x; D65, D69.5, D69.6; K72.0, K72.9, K76.7; R40.2, G93.4, F05.x; E87.2; cardiac arrest I46.x not counted. Septic shock was identified from the free-text discharge diagnosis because the code R57.2 was not recorded for any patient. Interaction and CV models adjusted as in Table 5; hypoglycemia and hyperglycemia models additionally for diabetes status; the diabetes OR is from the Model 2 covariate set. In subgroup B, peritonitis did not vary within one diabetes stratum and was omitted from that stratum-specific model.

Panel B. In-hospital mortality and prevalence of diabetes according to the route of sepsis identification.

Group	*n*	In-hospital mortality	Diabetes
Sepsis code as principal diagnosis	4,360	54.7%	29.1%
Sepsis code as secondary diagnosis only	6,020	52.5%	27.3%
No explicit sepsis code (text search or coded infection with organ dysfunction)	452	57.7%	31.9%
Septic shock documented in the discharge diagnosis	1,154	73.1%	24.4%

CI, confidence interval; CV, coefficient of variation; OR, odds ratio.

Table S29. Coded acute organ dysfunction, mortality and early glycemic variability (Panel A), and principal models with additional adjustment for coded acute organ dysfunction (Panel B).

Panel A. Number of organ systems with coded acute dysfunction.

Affected organ systems (coded)	*N*	In-hospital mortality	CV-24h available	Median CV-24h, no diabetes	Median CV-24h, diabetes
0	4,077	27.6%	51.0%	0.169	0.212
1	3,604	59.5%	62.5%	0.192	0.225
2	2,005	77.3%	67.3%	0.204	0.239
≥3	1,146	85.9%	66.8%	0.238	0.265

Organ systems: respiratory failure (*n* = 4,008), shock, including septic shock in the discharge text (*n* = 1,585), acute kidney injury (*n* = 2,773), coagulopathy (*n* = 602), acute hepatic failure (*n* = 839), coma, encephalopathy or delirium (*n* = 328), acidosis (*n* = 1,295). Codes: J96.0, J96.9, J80 (respiratory); R57.x or septic shock in the discharge text (shock); N17.x (renal); D65, D69.5, D69.6 (coagulation); K72.0, K72.9, K76.7 (hepatic); R40.2, G93.4, F05.x (neurological); E87.2 (acidosis); cardiac arrest I46.x not counted.

Panel B. Principal models with additional adjustment (patients with computable CV-24h for CV models; full cohort for hypoglycemia, hyperglycemia and diabetes models).

Additional adjustment	Interaction OR per 0.10 (95% CI), *p*	CV OR per 0.10, no diabetes (95% CI)	CV OR per 0.10, diabetes (95% CI)	Hypoglycemia OR (95% CI)	Hyperglycemia OR (95% CI)	Diabetes OR (95% CI)
None (primary)	0.912 (0.868-0.959), <0.001	1.137 (1.102-1.173)	1.034 (0.994-1.076)	2.066 (1.792-2.382)	1.536 (1.392-1.695)	0.822 (0.751-0.899)
Number of coded organ systems (0, 1, 2, ≥3)	0.924 (0.876-0.975), 0.004	1.095 (1.059-1.132)	1.006 (0.963-1.050)	1.634 (1.399-1.908)	1.481 (1.329-1.651)	0.839 (0.760-0.926)

Additional adjustment	Interaction OR per 0.10 (95% CI), *p*	CV OR per 0.10, no diabetes (95% CI)	CV OR per 0.10, diabetes (95% CI)	Hypoglycemia OR (95% CI)	Hyperglycemia OR (95% CI)	Diabetes OR (95% CI)
Number of coded organ systems + MV, RRT, surgery	0.944 (0.893-0.999), 0.045	1.083 (1.046-1.121)	1.014 (0.968-1.061)	1.691 (1.438-1.989)	1.461 (1.305-1.636)	0.798 (0.720-0.884)

Panel B: all models include the Model 2 covariates and, for glycemic predictors, the number of glucose measurements within 24 h; hypoglycemia and hyperglycemia models also include diabetes status; the diabetes OR is from the Model 2 covariate set plus the additional adjustment. Number of coded organ systems entered as categories (0, 1, 2, ≥3). Codes for organ dysfunction are assigned at discharge without timing and may reflect dysfunction arising after the exposure window; the analyses are therefore sensitivity analyses and the variable is not a substitute for physiological severity scores. CI, confidence interval; CV, coefficient of variation; MV, mechanical ventilation; OR, odds ratio; RRT, renal replacement therapy.

Pre-Existing Diabetes and Timing of Death

Table S30. Association between pre-existing diabetes and in-hospital mortality by timing of death and in landmark cohorts.

Panel A. Coded comorbidities and number of coded secondary diagnoses according to timing of death.

Group	*N*	Diabetes	CKD	COPD	Malignancy	Coded secondary diagnoses, median (IQR)
Death within 24 h of admission	1,450	22.3%	14.3%	5.0%	18.7%	8 (6-11)
Death 24-48 h	598	25.6%	16.6%	8.5%	16.6%	9 (6-12)
Death 2-7 days	1,463	30.7%	18.9%	5.9%	19.5%	9 (7-13)
Death after 7 days	2,292	27.6%	18.7%	7.1%	20.6%	11 (8-16)
Survivors	5,029	29.8%	12.4%	5.0%	14.7%	8 (5-12)

Timing of death measured from the admission timestamp. Coded secondary diagnoses: number of distinct ICD-10-AM codes among the secondary discharge diagnoses. Diabetes prevalence across the five groups, chi-square test $p < 0.001$; number of coded secondary diagnoses across the four non-survivor groups, Kruskal-Wallis test $p < 0.001$.

Panel B. Odds ratio for in-hospital death, patients with versus without pre-existing diabetes.

Cohort	*N*	Deaths	Mortality, diabetes / no diabetes	Crude OR (95% CI)	*p*	Adjusted OR (95% CI)	*p*
Full cohort	10,832	5,803	51.0% / 54.6%	0.866 (0.796-0.942)	<0.001	0.822 (0.751-0.899)	<0.001
Excluding deaths within 24 h of admission	9,382	4,353	45.2% / 46.9%	0.935 (0.855-1.022)	0.138	0.899 (0.816-0.990)	0.030
Excluding deaths within 48 h of admission	8,784	3,755	42.0% / 43.1%	0.955 (0.870-1.048)	0.332	0.921 (0.833-1.018)	0.105
Excluding deaths within 7 days of admission	7,321	2,292	29.7% / 32.0%	0.900 (0.807-1.005)	0.060	0.862 (0.766-0.970)	0.014
Alive 24 h after first glucose	9,543	4,587	46.5% / 48.7%	0.917 (0.840-1.002)	0.056	0.879 (0.799-0.966)	0.008
Alive 48 h after first glucose	8,697	3,878	43.3% / 45.1%	0.931 (0.848-1.022)	0.132	0.900 (0.814-0.994)	0.038
Alive 72 h after first glucose	8,123	3,468	41.0% / 43.4%	0.904 (0.820-0.995)	0.040	0.873 (0.787-0.969)	0.011
Alive 7 days after first glucose	5,943	2,331	35.9% / 40.7%	0.815 (0.727-0.914)	<0.001	0.796 (0.704-0.900)	<0.001

Panel B: crude ORs from logistic regression with diabetes status as the only covariate; adjusted ORs from logistic regression with the Model 2 covariates (calendar year, age, sex, malignancy, pneumonia, cirrhosis, CKD, COPD, peritonitis). Deaths within 24 h of admission are timed from the admission timestamp; the cohorts alive at 24 h, 48 h, 72 h and 7 days comprise patients alive and hospitalized at that time after the first glucose measurement. In the full cohort, additional adjustment of the Model 2 covariates for the number of coded secondary diagnoses gave an adjusted OR of 0.721 (95% CI 0.658-0.791).

CI, confidence interval; CKD, chronic kidney disease; COPD, chronic obstructive pulmonary disease; ICD-10-AM, International Statistical Classification of Diseases and Related Health Problems, Tenth Revision, Australian Modification; IQR, interquartile range; OR, odds ratio.

Absolute Measures, Non-Linearity and Multiplicity

Table S31. Absolute measures of the association between early glycemic variability and in-hospital mortality according to diabetes status (patients with computable CV-24h, *n* = 6,450).

Panel A. Standardized predicted mortality and marginal effects.

Measure	No diabetes (*n* = 4,455)	Diabetes (*n* = 1,995)	Difference between strata
Standardized mortality, CV-24h 0.10	51.3% (49.6-53.0)	49.2% (46.3-52.0)	
Standardized mortality, CV-24h 0.20	54.2% (52.7-55.7)	50.0% (47.6-52.3)	
Standardized mortality, CV-24h 0.30	57.0% (55.5-58.4)	50.8% (48.7-52.8)	
Standardized mortality, CV-24h 0.40	59.8% (58.1-61.5)	51.7% (49.3-53.9)	
Standardized mortality, CV-24h 0.50	62.5% (60.3-64.8)	52.5% (49.5-55.3)	
Risk difference, 0.40 vs. 0.10	8.5 pp (6.3 to 10.7)	2.5 pp (−0.4 to 5.4)	
Marginal risk difference per 0.10 increase	2.79 pp (2.09 to 3.49)	0.82 pp (−0.13 to 1.77)	1.97 pp (0.83 to 3.08)
Marginal risk ratio per 0.10 increase	1.050 (1.038-1.063)	1.016 (0.997-1.035)	
Risk ratio per 0.10 increase, modified Poisson	1.047 (1.037-1.057)	1.015 (0.997-1.032)	0.970 (0.952-0.989), p = 0.002
Odds ratio per 0.10 increase, logistic	1.137 (1.102-1.173)	1.034 (0.994-1.076)	ratio 0.912 (0.868-0.959), p < 0.001

Standardized estimates by g-computation from the primary interaction model (adjusted for calendar year, age, sex, malignancy, pneumonia, cirrhosis, CKD, COPD, peritonitis and number of glucose measurements within 24 h); 95% CIs from 500 bootstrap resamples. Percentiles of CV-24h in the analyzed patients: 10th 0.040, 25th 0.100, 50th 0.203, 75th 0.357, 90th 0.554. Risk ratios from modified Poisson regression with robust standard errors.

Panel B. Observed in-hospital mortality by quartile of CV-24h (cut-points 0.100, 0.203 and 0.357).

Quartile of CV-24h	No diabetes, *n*	No diabetes, mortality	Diabetes, *n*	Diabetes, mortality
Q1	1,166	53.0%	447	52.8%
Q2	1,174	50.7%	438	43.6%
Q3	1,090	54.6%	522	50.4%
Q4	1,025	64.5%	588	54.8%

CKD, chronic kidney disease; COPD, chronic obstructive pulmonary disease; CV, coefficient of variation; pp, percentage points.

Table S32. Exploratory restricted cubic spline analysis of CV-24h and in-hospital mortality.

Panel A. Distribution and specification.

	No diabetes (*n* = 4,455)	Diabetes (*n* = 1,995)
CV-24h <0.05, n (%)	610 (13.7%)	216 (10.8%)
CV-24h <0.10, n	1,167	447
CV-24h >0.80, n	130	86
Median CV-24h (reference)	0.193	0.229
Knots (4; 5th, 35th, 65th, 95th percentiles)	0.018, 0.133, 0.269, 0.677	0.025, 0.158, 0.316, 0.764
Overall CV association p, 3 / 4 / 5 knots	<0.001 / <0.001 / <0.001	0.045 / 0.034 / 0.032
Non-linearity p, 3 / 4 / 5 knots	0.0001 / 0.0003 / 0.0004	0.063 / 0.052 / 0.049
AIC, linear vs. 4-knot spline	5,635.5 vs. 5,623.3	2,640.9 vs. 2,639.0

	No diabetes (n = 4,455)	Diabetes (n = 1,995)
Non-linearity p (3 knots), two measurements	<0.001 (n = 3,204)	0.022 (n = 1,409)
Non-linearity p (3 knots), ≥3 measurements	0.393 (n = 1,251)	0.771 (n = 586)
Linear OR per 0.10, two measurements	1.132 (1.091-1.173)	1.030 (0.984-1.078)
Linear OR per 0.10, ≥3 measurements	1.154 (1.086-1.226)	1.044 (0.964-1.131)

Spline-by-diabetes interaction (four knots at pooled percentiles): likelihood-ratio $p < 0.001$. Median interval between the first two measurements: 14.5 h for CV-24h <0.05 and 12.5-13.3 h for CV-24h 0.05-0.45; second value within 1 h in ≤0.73% of patients in every CV band. Non-linearity tested by the likelihood-ratio test of the spline against the linear model. In patients without diabetes with three or more measurements, the non-linearity p was 0.393 with three knots and 0.701 with four knots. Among patients without diabetes with two measurements, hypoglycemia was present in 43.2% of those with CV-24h > 0.45 and in 9.2% of those with CV-24h 0.05-0.45.

Panel B. Odds ratio relative to the stratum-specific median (four-knot model).

CV-24h	No diabetes, OR (95% CI)	Diabetes, OR (95% CI)
1st percentile (0.005 / 0.006)	1.151 (0.904-1.464)	1.427 (0.995-2.047)
10th percentile (0.038 / 0.046)	1.093 (0.920-1.297)	1.271 (0.987-1.637)
0.10	1.007 (0.941-1.079)	1.105 (0.968-1.262)
0.15	0.983 (0.953-1.014)	1.012 (0.944-1.085)
0.20	1.005 (0.999-1.012)	0.988 (0.959-1.019)
0.30	1.139 (1.026-1.264)	1.074 (0.975-1.183)
0.40	1.342 (1.151-1.565)	1.193 (0.981-1.450)
0.50	1.622 (1.372-1.917)	1.290 (1.022-1.629)
95th percentile (0.677 / 0.764)	2.338 (1.915-2.855)	1.476 (1.122-1.942)
99th percentile (1.007 / 1.085)	4.707 (3.164-7.002)	1.691 (1.011-2.829)

Models adjusted for calendar year, age, sex, malignancy, pneumonia, cirrhosis, CKD, COPD, peritonitis and number of glucose measurements within 24 h (omitted in the subgroup with exactly two measurements). Percentile values are given as no diabetes / diabetes. AIC, Akaike information criterion; CI, confidence interval; CKD, chronic kidney disease; COPD, chronic obstructive pulmonary disease; CV, coefficient of variation; OR, odds ratio.

Table S33. Adjustment for multiple testing across the diabetes-by-glycemic-parameter interaction tests (primary models).

Window	Glycemic parameter	Unadjusted p	Holm-adjusted p (12 tests)	Benjamini-Hochberg adjusted p (12 tests)	Holm-adjusted p (4 tests, 24 h only)
24 h	CV	0.0003	0.003	0.001	0.001
24 h	Hyperglycemia	0.001	0.009	0.003	0.002
24 h	TWM	0.0006	0.005	0.002	0.002
24 h	Hypoglycemia	0.099	0.298	0.119	0.099
48 h	CV	0.003	0.019	0.005	—
48 h	Hyperglycemia	0.019	0.078	0.026	—
48 h	TWM	0.0002	0.003	0.001	—
48 h	Hypoglycemia	0.425	0.850	0.463	—
72 h	CV	0.003	0.019	0.005	—
72 h	Hyperglycemia	0.016	0.078	0.023	—
72 h	TWM	0.0001	0.001	0.001	—
72 h	Hypoglycemia	0.478	0.850	0.478	—

Interaction terms from logistic models adjusted for calendar year, age, sex, malignancy, pneumonia, cirrhosis, CKD, COPD, peritonitis and number of glucose measurements within the window (Table 5, Panel A, and Tables S16 and S18). CKD, chronic kidney disease; COPD, chronic obstructive pulmonary disease; CV, coefficient of variation; TWM, time-weighted mean glucose.

Adjustment Strategy and Predictive Performance

Table S34. Sensitivity of the principal estimates to the adjustment set derived from the conceptual causal model (main text, Figure 2).

Adjustment set	Diabetes OR, full cohort (95% CI)	Diabetes OR, alive 24 h after first glucose (95% CI)	CV OR per 0.10, no diabetes (95% CI)	CV OR per 0.10, diabetes (95% CI)	Interaction OR per 0.10 (95% CI)
A. Confounders only: age, sex, calendar year	0.813 (0.746-0.887)	0.869 (0.792-0.952)	1.130 (1.096-1.166)	1.029 (0.990-1.070)	0.912 (0.868-0.957)
B. A + malignancy, cirrhosis, COPD	0.827 (0.758-0.902)	0.880 (0.803-0.966)	1.130 (1.096-1.165)	1.030 (0.991-1.071)	0.914 (0.870-0.959)
C. B + chronic kidney disease (possible mediator)	0.808 (0.740-0.883)	0.859 (0.783-0.942)	1.130 (1.096-1.165)	1.031 (0.991-1.072)	0.914 (0.871-0.960)
D. C + infection focus (possible mediator) = Model 2	0.822 (0.751-0.899)	0.879 (0.799-0.966)	1.137 (1.102-1.173)	1.034 (0.994-1.076)	0.912 (0.868-0.959)

Adjustment sets derived from the conceptual causal model (main text, Figure 2). CV models additionally adjusted for the number of glucose measurements within 24 h; with set A and without this covariate, the interaction OR was 0.912 (0.868-0.958). CI, confidence interval; COPD, chronic obstructive pulmonary disease; CV, coefficient of variation; OR, odds ratio.

Table S35. Incremental predictive performance of CV-24h beyond baseline clinical variables, according to diabetes status.

Measure	No diabetes (*n* = 4,455)	Diabetes (*n* = 1,995)
Likelihood-ratio test for adding CV-24h	$\chi^2 = 69.75$, p < 0.001	$\chi^2 = 2.74$, p = 0.098
C-statistic, base → + CV-24h (apparent)	0.672 → 0.685	0.651 → 0.653
C-statistic, optimism-corrected	0.669 → 0.682	0.642 → 0.643
Change in C-statistic (95% CI)	0.013 (0.008 to 0.019)	0.002 (−0.000 to 0.006)
Brier score, base → + CV-24h	0.2238 → 0.2203	0.2323 → 0.2320
Change in Brier score (95% CI)	−0.0035 (−0.0051 to −0.0023)	−0.0003 (−0.0015 to −0.0000)
Calibration slope, optimism-corrected, base / + CV	0.980 / 0.980	0.941 / 0.926
Hosmer-Lemeshow, + CV model	$\chi^2 = 12.35$, p = 0.136	$\chi^2 = 9.62$, p = 0.293
Continuous NRI (95% CI)	0.172 (0.120 to 0.229)	0.043 (−0.033 to 0.136)
NRI, events / non-events	−0.144 / 0.316	−0.194 / 0.237
IDI (95% CI)	0.014 (0.009 to 0.021)	0.001 (0.000 to 0.006)
Change in C-statistic over base + coded organ dysfunction	0.823 → 0.825 (Δ 0.001, 95% CI 0.000 to 0.003); LR p < 0.001	0.828 → 0.828 (Δ 0.000, 95% CI −0.000 to 0.002); LR p = 0.52
Change in C-statistic over base + organ dysfunction + MV, RRT, surgery	0.844 → 0.845 (Δ 0.001, 95% CI 0.000 to 0.002); LR p < 0.001	0.850 → 0.850 (Δ 0.000, 95% CI −0.000 to 0.002); LR p = 0.32

Base model: calendar year, age, sex, malignancy, pneumonia, cirrhosis, CKD, COPD, peritonitis and number of glucose measurements within 24 h. CV-24h entered per 0.10 increase. Confidence intervals and optimism correction from 200 bootstrap resamples. The last two rows use base models additionally including indicators for the seven organ systems with coded acute dysfunction (Table S29), alone and with

mechanical ventilation, renal replacement therapy and surgical intervention. CI, confidence interval; CKD, chronic kidney disease; COPD, chronic obstructive pulmonary disease; CV, coefficient of variation; IDI, integrated discrimination improvement; LR, likelihood ratio; MV, mechanical ventilation; NRI, net reclassification improvement; RRT, renal replacement therapy.

Analytic Populations and Additional Sensitivity Analyses

Table S36. Reconciliation of the analytic populations (Panel A) and number of patients and deaths in each principal model (Panel B).

Panel A. Reconciliation of the analytic populations.

Analysis	*N*	Difference from the cohort and reason
Cohort; Models 1, 2, 4; hypoglycemia and hyperglycemia models	10,832	—
TWM-24h single-parameter and interaction models	10,802	−30: TWM-24h missing
CV-24h computable	6,450	−4,382: 4,377 with one measurement in 24 h; 5 with ≥2 measurements, one outside the measuring range
No diabetes / diabetes	4,455 / 1,995	
Models 3 and 5, 24 h	6,434	−16 from CV-24h: TWM-24h missing
CV-48h computable / Models 3 and 5, 48 h	7,978 / 7,957	−21 from CV-48h: TWM-48h missing
CV-72h computable / Models 3 and 5, 72 h	8,530 / 8,507	−23 from CV-72h: TWM-72h missing
Landmark cohort, alive 24 h after first glucose, with CV-24h	6,123	−327 from CV-24h: died or discharged within 24 h
≥3 measurements in 24 h, with CV-24h	1,837	restriction (Table S26)

Panel B. Number of patients and deaths in each principal model.

Analysis	N	Deaths	In-hospital mortality
Models 1, 2 and 4; hypoglycemia and hyperglycemia models (full cohort)	10,832	5,803	53.6%
TWM-24h models	10,802	5,781	53.5%
CV-24h models (interaction model)	6,450	3,481	54.0%
CV-24h, no diabetes (stratified model)	4,455	2,469	55.4%
CV-24h, diabetes (stratified model)	1,995	1,012	50.7%
Models 3 and 5, 24 h	6,434	3,466	53.9%
CV-48h models	7,978	4,179	52.4%
Models 3 and 5, 48 h	7,957	4,162	52.3%
CV-72h models	8,530	4,400	51.6%
Models 3 and 5, 72 h	8,507	4,382	51.5%
>=3 measurements within 24 h	1,837	1,000	54.4%

TWM could not be computed in 30 patients because one glucose result (31 results in total: 28 below and 3 above the measuring range) had been reported by the laboratory as outside the analyzer's measuring range rather than as a number; these results were counted in the number of measurements but treated as missing for the other glycemic metrics. In a sensitivity analysis in which values below the lower limit were set to half that limit and values above the upper limit to the limit itself (n with CV-24h = 6,455), the diabetes-by-CV-24h interaction OR per 0.10 was 0.912 (0.868-0.958); CV-24h OR per 0.10 1.136 (1.101-1.171) without and 1.033 (0.993-1.074) with diabetes; hypoglycemia OR 2.080 (1.805-2.397); hyperglycemia OR 1.533 (1.389-1.691); hypoglycemic patients 1,090 vs 1,083 (quantified vs. primary). CV, coefficient of variation; OR, odds ratio; TWM, time-weighted mean glucose. Panel B: landmark cohorts comprise patients alive and hospitalized at the stated time after the first glucose measurement, with a computable CV for the stated exposure.

Table S37. Diabetes-by-CV interaction and diabetes-stratified CV associations according to mechanical ventilation.

Stratum	*N* (no diabetes / diabetes)	In-hospital mortality	Interaction OR per 0.10 (95% CI), *p*	CV OR per 0.10, no diabetes (95% CI)	CV OR per 0.10, diabetes (95% CI)
Mechanical ventilation	1,829 (1,261 / 568)	79.7%	0.877 (0.778-0.988), 0.031	1.136 (1.056-1.222)	0.996 (0.903-1.098)
No mechanical ventilation	4,621 (3,194 / 1,427)	43.8%	0.947 (0.892-1.004), 0.070	1.120 (1.080-1.161)	1.051 (1.002-1.103)

Patients with computable CV-24h. Models adjusted for calendar year, age, sex, malignancy, pneumonia, cirrhosis, CKD, COPD, peritonitis and number of glucose measurements within 24 h. Three-way interaction (CV × diabetes × mechanical ventilation), Wald $p = 0.27$. ICU admission was not reliably recorded in the administrative dataset; mechanical ventilation, a post-baseline variable, was used as a proxy and the stratification is descriptive. CI, confidence interval; CKD, chronic kidney disease; COPD, chronic obstructive pulmonary disease; CV, coefficient of variation; OR, odds ratio.

Table S38. Cohort characteristics and principal CV estimates according to calendar period.

Period	*n*	Mortality	Sepsis as principal diagnosis	Coded acute organ dysfunction	Diabetes	*n* with CV-24h	Interaction OR per 0.10 (95% CI)	*p*	CV OR per 0.10, no diabetes (95% CI)	CV OR per 0.10, diabetes (95% CI)
2011-2015	2,458	54.5%	29.3%	52.4%	21.8%	1,314	0.980 (0.862-1.114)	0.760	1.100 (1.028-1.177)	1.079 (0.960-1.213)
2016-2019	3,205	54.1%	49.2%	58.8%	27.2%	1,802	0.914 (0.832-1.005)	0.062	1.101 (1.039-1.166)	1.011 (0.936-1.092)
2020-2024	5,169	52.8%	39.9%	69.3%	31.9%	3,334	0.890 (0.832-0.952)	<0.001	1.168 (1.117-1.222)	1.032 (0.980-1.086)

Models adjusted as in Table 5. Coded acute organ dysfunction as defined in Table S29 (including septic shock in the discharge text). Heterogeneity of the diabetes × CV interaction across periods: likelihood-ratio test of the two three-way interaction terms (2 df), $p = 0.420$; linear calendar-year trend of the interaction (Wald test), $p = 0.229$. CI, confidence interval; CV, coefficient of variation; OR, odds ratio.

Table S39. Sensitivity of the principal CV estimates to extreme values, sampling intervals and hyperglycemia.

Analysis	*n* (no diabetes / diabetes)	Mortality	Interaction OR per 0.10 (95% CI), *p*	CV OR per 0.10, no diabetes (95% CI)	CV OR per 0.10, diabetes (95% CI)
Primary	6,450 (4,455 / 1,995)	54.0%	0.912 (0.868-0.959), <0.001	1.137 (1.102-1.173)	1.034 (0.994-1.076)
Adjusted for time span of measurements	6,450 (4,455 / 1,995)	54.0%	0.914 (0.870-0.961), <0.001	1.136 (1.101-1.172)	1.035 (0.994-1.076)
Adjusted for interval between first two values	6,450 (4,455 / 1,995)	54.0%	0.914 (0.870-0.961), <0.001	1.136 (1.101-1.172)	1.034 (0.994-1.076)
Second value ≥6 h after the first	5,589 (3,869 / 1,720)	53.6%	0.917 (0.868-0.967), 0.002	1.139 (1.101-1.178)	1.040 (0.997-1.085)
Adjusted for hypoglycemia and hyperglycemia	6,450 (4,455 / 1,995)	54.0%	0.930 (0.885-0.978), 0.005	1.064 (1.023-1.106)	0.988 (0.942-1.037)

Analysis	*n* (no diabetes / diabetes)	Mortality	Interaction OR per 0.10 (95% CI), *p*	CV OR per 0.10, no diabetes (95% CI)	CV OR per 0.10, diabetes (95% CI)
Excluding any value <54 or >400 mg/dL	5,710 (4,110 / 1,600)	52.6%	0.930 (0.868-0.997), 0.040	1.083 (1.041-1.127)	1.004 (0.948-1.064)
Excluding any value <70 mg/dL	5,621 (3,830 / 1,791)	52.6%	0.952 (0.893-1.015), 0.134	1.072 (1.030-1.116)	1.022 (0.971-1.076)
All values ≤180 mg/dL	4,031 (3,283 / 748)	51.3%	0.921 (0.847-1.001), 0.053	1.127 (1.078-1.178)	1.037 (0.965-1.114)

Patients with computable CV-24h. Models adjusted for calendar year, age, sex, malignancy, pneumonia, cirrhosis, CKD, COPD, peritonitis and number of glucose measurements within 24 h. Median time span of the measurements within 24 h, 16.2 h; median interval between the first two values, 12.8 h. CI, confidence interval; CKD, chronic kidney disease; COPD, chronic obstructive pulmonary disease; CV, coefficient of variation; OR, odds ratio.

Table S40. Standardized in-hospital mortality, risk difference and risk ratio for pre-existing diabetes in the full cohort and in landmark cohorts.

Cohort	N	Deaths	OR, Model 2 (95% CI)	Standardized mortality, no diabetes, % (95% CI)	Standardized mortality, diabetes, % (95% CI)	Risk difference, pp (95% CI)	Risk ratio, g-computation (95% CI)	Risk ratio, modified Poisson (95% CI)	p (modified Poisson)
Full cohort	10,832	5,803	0.822 (0.751-0.899)	54.8 (53.8-56.0)	50.5 (48.9-52.3)	-4.32 (-6.31 to -2.28)	0.921 (0.887-0.958)	0.935 (0.900-0.973)	<0.001
Alive 24 h after first glucose	9,543	4,587	0.879 (0.799-0.966)	48.9 (47.8-50.1)	46.1 (44.3-47.8)	-2.83 (-4.89 to -0.58)	0.942 (0.901-0.988)	0.956 (0.914-1.000)	0.051
Alive 48 h after first glucose	8,697	3,878	0.900 (0.814-0.994)	45.3 (44.1-46.5)	43.0 (41.2-44.8)	-2.28 (-4.51 to -0.14)	0.950 (0.902-0.997)	0.963 (0.916-1.013)	0.144

Standardized mortality was obtained by g-computation from Model 2 (covariates: calendar year, age, sex, malignancy, pneumonia, cirrhosis, CKD, COPD, peritonitis), setting diabetes status to present or absent for every patient and averaging the predicted risks; 95% confidence intervals from 500 bootstrap resamples (percentile method). The modified Poisson risk ratio was estimated with Poisson regression with robust (HC0) standard errors and the same covariates. Landmark cohorts comprise all patients alive and hospitalized at the stated time after the first glucose measurement, irrespective of CV availability. CI, confidence interval; CKD, chronic kidney disease; COPD, chronic obstructive pulmonary disease; OR, odds ratio; pp, percentage points.

References

1. Jiang, L.; Cheng, M. Impact of diabetes mellitus on outcomes of patients with sepsis: an updated systematic review and meta-analysis. *Diabetol. Metab. Syndr.* **2022**, *14*, 39. https://doi.org/10.1186/s13098-022-00803-2.
2. Zheng, H.; Yu, F.; Bei, C.; Zhou, Y.; Mo, Z.; Wei, B. Diabetes mellitus and mortality in patients admitted to ICU with sepsis: a meta-analysis. *Front. Med.* **2026**, *13*, 1743706. https://doi.org/10.3389/fmed.2026.1743706.
