# Supplementary material for: Early Glycemic Variability from Routine Glucose Measurements, Diabetes Status, and In-Hospital Mortality in Sepsis: A 14-Year Retrospective Cohort Study
Source: J Clin Med. 2026 Nov 6;15(21):8649. doi: 10.3390/jcm15218649 (PMC13764494; doi:10.3390/jcm15218649)
Supplement: Supplementary file 1 [file jcm-15-08649-s001.zip › STROBE_checklist_JCM_R2.pdf]

STROBE Statement-checklist of items that should be included in reports of observational studies

Manuscript: Early Glycemic Variability from Routine Glucose Measurements, Diabetes Status, and In-Hospital Mortality in Sepsis: A 14-Year Retrospective Cohort Study (Journal of Clinical Medicine, second revised version). Study design: cohort study. Locations refer to section, table, and figure numbers of the revised manuscript and Supplementary Materials.

	Item No	Recommendation	Reported in manuscript (section / table / figure)
**Title and abstract**	1	(*a*) Indicate the study's design with a commonly used term in the title or the abstract	Title ("Retrospective Cohort Study"); Abstract (Methods: "Single-center retrospective cohort")
		(*b*) Provide in the abstract an informative and balanced summary of what was done and what was found	Abstract (structured: Background/Objectives, Methods, Results, Conclusions; includes the main limitations of the CV analyses)
**Introduction**			
Background/rationale	2	Explain the scientific background and rationale for the investigation being reported	Section 1 (Introduction)
Objectives	3	State specific objectives, including any prespecified hypotheses	Section 1, final paragraph (primary objective, principal hypothesis, secondary objectives); hierarchy of analyses in Section 2.9
**Methods**			
Study design	4	Present key elements of study design early in the paper	Title; Abstract; Section 2.1
Setting	5	Describe the setting, locations, and relevant dates, including periods of recruitment, exposure, follow-up, and data collection	Section 2.1 (University Emergency Hospital of Bucharest; 1 January 2011-31 December 2024; EHR data); Section 2.3 (follow-up until hospital discharge); Section 2.5 (exposure windows)
Participants	6	(*a*) *Cohort study*-Give the eligibility criteria, and the sources and methods of selection of participants. Describe methods of follow-up<br>*Case-control study*-Give the eligibility criteria, and the sources and methods of case ascertainment and control selection. Give the rationale for the choice of cases and controls<br>*Cross-sectional study*-Give the eligibility criteria, and the sources and methods of selection of participants	Section 2.2 and Figure 1 (eligibility, multistep case-finding with the list of sepsis codes, exclusion criteria; code frequencies in Table S1); Section 2.3 (follow-up during the index hospitalization)
		(*b*) *Cohort study*-For matched studies, give matching criteria and number of exposed and unexposed	Not applicable (unmatched cohort)

		Case-control study-For matched studies, give matching criteria and the number of controls per case	
Variables	7	Clearly define all outcomes, exposures, predictors, potential confounders, and effect modifiers. Give diagnostic criteria, if applicable	Outcome: Section 2.3. Exposures: Sections 2.4-2.6. Confounders and effect modifier: Section 2.4 and Figure 2 (conceptual causal model); Section 2.9.3. Diagnostic criteria: Section 2.2 and Table S1 (sepsis), Section 2.4 and Table S2 (diabetes), Section 2.4 (coded acute organ dysfunction), Section 2.6 (glycemic thresholds)
Data sources/ measurement	8*	For each variable of interest, give sources of data and details of methods of assessment (measurement). Describe comparability of assessment methods if there is more than one group	Sections 2.1, 2.2 and 2.4-2.6 (laboratory glucose measurements, Section 2.5); Supplementary Methods S1 (time-weighted mean glucose). The same EHR sources and definitions were applied to patients with and without diabetes
Bias	9	Describe any efforts to address potential sources of bias	Section 2.7; Section 2.4 and Figure 2 (adjustment set; in-hospital variables with uncertain timing confined to sensitivity analyses); Section 2.9.5 (informative observation and survivor bias: landmark analyses, inverse-probability-of-observation weighting); Section 2.9.7 (sensitivity to case ascertainment, window anchoring and glucose sampling)
Study size	10	Explain how the study size was arrived at	Section 2.8 (all eligible consecutive admissions; no formal sample size calculation); Figure 1; Table S36
Quantitative variables	11	Explain how quantitative variables were handled in the analyses. If applicable, describe which groupings were chosen and why	Section 2.6 (units; CV expressed as a proportion, odds ratios per 0.10 increase; glycemic thresholds); Section 2.9.2 (age and calendar year as continuous variables); Section 2.9.4 (restricted cubic splines); Supplementary Methods S2
Statistical methods	12	(*a*) Describe all statistical methods, including those used to control for confounding	Sections 2.9 and 2.9.2 (hierarchy of analyses; sequential logistic regression models and covariate sets); Section 2.9.1 (descriptive tests, multiplicity adjustment, software); Supplementary Methods S2 (specification of all analyses)
		(*b*) Describe any methods used to examine subgroups and interactions	Section 2.9.3 (multiplicative interaction terms, diabetes-stratified models, additive-scale estimates by g-computation for diabetes status and for CV-24h); Section 2.9.4 (splines by diabetes status); Supplementary Methods S2

		(*c*) Explain how missing data were addressed	Section 2.9.6 (complete-case analysis for each model; CV-24h unavailable in 4,382 and TWM-24h in 30 patients); Section 2.9.5 (availability of CV treated as informative: landmark analyses and inverse-probability-of-observation weighting); Table S9 (patients with and without CV-24h); Table S36
		(*d*) *Cohort study*-If applicable, explain how loss to follow-up was addressed<br>*Case-control study*-If applicable, explain how matching of cases and controls was addressed<br>*Cross-sectional study*-If applicable, describe analytical methods taking account of sampling strategy	Not applicable to the outcome (in-hospital mortality, ascertained for all patients until discharge). Post-discharge vital status was not available (Section 2.3)
		(*e*) Describe any sensitivity analyses	Section 2.9.7 (items i-ix) and Section 2.9.5; Section 2.9.2 (Models 4 and 5; alternative adjustment sets, Table S34); 48 h and 72 h exposure windows; Supplementary Methods S2
**Results**			
Participants	13*	(a) Report numbers of individuals at each stage of study-eg numbers potentially eligible, examined for eligibility, confirmed eligible, included in the study, completing follow-up, and analysed	Section 2.2 and Figure 1 (665,023 admissions screened; 10,914 hospitalizations identified; 82 excluded; 10,832 analyzed; analytic populations); N for each model in Tables 4-6; reconciliation of all analytic populations in Table S36
		(b) Give reasons for non-participation at each stage	Section 2.2 and Figure 1 (exclusion criteria; criteria not mutually exclusive; total n = 82)
		(c) Consider use of a flow diagram	Figure 1
Descriptive data	14*	(a) Give characteristics of study participants (eg demographic, clinical, social) and information on exposures and potential confounders	Section 3.1 and Tables 1 and 2 (by diabetes status and by survival status); Section 3.2, Table 3 and Tables S3-S4 (early glycemic profile)
		(b) Indicate number of participants with missing data for each variable of interest	Section 2.9.6; Tables 2 and 3 (availability of CV-24h and TWM-24h); Tables S3-S4 (48 h and 72 h windows); Tables S9 and S36
		(c) *Cohort study*-Summarise follow-up time (eg, average and total amount)	Follow-up limited to the index hospitalization; length of stay in Tables 1 and 2
Outcome data	15*	*Cohort study*-Report numbers of outcome events or summary measures	Section 3.1 and Tables 1 and 2 (in-hospital deaths, 5,803/10,832,

		over time	overall and by diabetes status); Section 2.8 and Table S36, Panel B (number of deaths in each principal model); mortality by sepsis identification route and by coded organ dysfunction (Section 3.7; Tables S28 and S29)
		Case-control study-Report numbers in each exposure category, or summary measures of exposure	Not applicable
		Cross-sectional study-Report numbers of outcome events or summary measures	Not applicable
Main results	16	(*a*) Give unadjusted estimates and, if applicable, confounder-adjusted estimates and their precision (eg, 95% confidence interval). Make clear which confounders were adjusted for and why they were included	Unadjusted and adjusted ORs with 95% CIs: Section 3.3 and Table 4 (Models 1-5; full output in Table S5; alternative adjustment sets in Table S34; timing of death in Table S30); Section 3.4, Figure 3 and Table S8. Confounders and rationale for inclusion: Section 2.4 and Figure 2
		(*b*) Report category boundaries when continuous variables were categorized	Section 2.6 (hypoglycemia < 3.9 and < 3.0 mmol/L; hyperglycemia > 10.0 mmol/L); Section 2.9.7 and Supplementary Methods S2 (number of organ systems with coded acute dysfunction: 0, 1, 2, ≥3; calendar periods 2011-2015, 2016-2019 and 2020-2024)
		(*c*) If relevant, consider translating estimates of relative risk into absolute risk for a meaningful time period	Absolute in-hospital mortality by group (Tables 1 and 2); standardized predicted mortality, risk differences and risk ratios by g-computation for diabetes status (Section 3.3; Table S40) and for CV-24h (Section 3.5; Table 5, Panel C; Table S31)
Other analyses	17	Report other analyses done-eg analyses of subgroups and interactions, and sensitivity analyses	Effect modification and stratified analyses (Section 3.5, Table 5, Tables S15-S21; multiplicity in Table S33); spline analyses (Section 3.6, Figure 4, Table S32); informative observation and robustness (Section 3.7, Table 6, Tables S23-S30 and S37-S39; diabetes and mortality in landmark cohorts, Table S40); incremental predictive performance (Section 3.8, Table S35); 48 h and 72 h windows (Tables S3, S4, S6, S7 and S10-S21)
**Discussion**			
Key results	18	Summarise key results with reference to study objectives	Section 4, first paragraph
Limitations	19	Discuss limitations of the study, taking into account sources of potential	Section 4.6 (absence of severity scores and residual confounding;

		bias or imprecision. Discuss both direction and magnitude of any potential bias	absence of treatment data; clinically driven glucose sampling and two-point CV; informative observation and survivor bias, with the patients and deaths excluded at each landmark; sepsis ascertainment from discharge documentation; misclassification of diabetes and its likely direction; limited power in the diabetes stratum and multiplicity; single-center routine data)
Interpretation	20	Give a cautious overall interpretation of results considering objectives, limitations, multiplicity of analyses, results from similar studies, and other relevant evidence	Sections 4.1-4.5 and 5; multiplicity addressed in Section 2.9.1, Table 5 (Panel A) and Table S33; comparison with previous studies and meta-analyses in Sections 4.1-4.3 and Table S22
Generalisability	21	Discuss the generalisability (external validity) of the study results	Sections 4.4 and 4.6
**Other information**			
Funding	22	Give the source of funding and the role of the funders for the present study and, if applicable, for the original study on which the present article is based	Funding statement (no external funding); Acknowledgments (institutional publication support)

*Give information separately for cases and controls in case-control studies and, if applicable, for exposed and unexposed groups in cohort and cross-sectional studies.
